# 13th C1-inhibitor deficiency and angioedema workshop—2023: Edited by: Róbert Hohol; Lectored by: Henriette Farkas, Lilian Varga; Cover artwork: Gergely Hosszú

**DOI:** 10.1186/s13223-023-00845-9

**Published:** 2023-12-20

In 2023, the 13th edition of the biennial international scientific conference on C1-inhibitor deficiency and other bradykinin-mediated angioedema took place from 4 to 7 May. The 13th C1-inhibitor Deficiency and Angioedema Workshop, chaired by Professor Henriette Farkas, was again held in Budapest. Nearly 400 participants from 50 countries from all over the world came to Hungary. Since 1999, when the Workshop was held for the first time, the composition of the registrants has been unique, with active and equal participation in the scientific discussions from allergists, immunologists, dermatologists, otorhinolaryngologists, internists, paediatricians and other specialists, as well as from research biologists, biochemists and pharmaceutical researchers, and representatives of the patient organisations involved in the disease. This year 93 speakers delivered presentations in 10 oral and in 2 poster sessions Professor A. P Kaplan summarised the key findings of this year’s conference.

Written by Dr. Allen P. Kaplan

Professor of Medicine

The Medical University of South Carolina

Charleston, SC, USA.

Our 3-day meeting reflects tremendous progress being made in the pathogenesis and treatment of all forms of Hereditary Angioedema (HAE) as well as acquired C1-inhibitor Deficiency. We have begun an era of genetic approaches to therapy. Among these are CRISPR knockout of the prekallikrein (PK) gene with excellent preliminary results—many participants were attack-free at 16 weeks with a PK reduction of 92%. Adenovirus-dependent insertion of C1 INH into hepatocytes has been achieved with steroid enhancement of both uptake and gene expression as well as reduction of inflammation-related side effects including transaminitis. C1 INH synthesis and secretion into the plasma was achieved; the duration of effect is not yet clear. A supporting lecture on CRISPR methodology explained the need for a guide RNA to localize the site of cleavage and insertion of the gene. Another unique approach with donidalorsen digests PK mRNA, decreases blood levels by 70%, and attack rate by 90%. There are also new therapies employing proteins or peptides. A new monoclonal antibody directed to factor XIIa has excellent efficacy and 56% of study patients were free of attacks. While Berotralstat is quite effective for prophylaxis, even in adolescents, a new oral agent sebetralstat at 3 doses/day can be used for short term prophylaxis e.g. for surgery or dental work. Preliminary data of an oral B-2 receptor antagonist with a deuterium atom incorporated has a long half-life of 10 h., inhibits intravenous bradykinin effects including decreased blood pressure within 15 min. and lasts 8 h. In a phase 2, dose ranging study, HAE attacks were successfully treated. In one STAR 0215 study a modified IgG1 monoclonal antibody to kallikrein has a half-life of 117 days and can be given every 3 months.

We heard about varying treatment options in different countries. Oral agents are generally favored by patients unless there is a large increase in efficacy with parenteral choices. Quality of life (QOL) increases with use of subcutaneous (SC) C1 INH or Lanadelumab in most studies. But there are issues of drug availability in some countries, costs of therapy, or even appropriate use of the drugs we do have, and then QOL decreases as the therapy varies from what seems to be optimal. In all studies there are patients whose response remains suboptimal, emphasizing that there is still a need for new, better agents. Patient anxiety relates most to the unpredictability of the disease and its chronicity; for some fear of asphyxia and shame are separate issues. Surprisingly it has been shown that there are those who do not carry their “on-demand” therapy with them when traveling, preferring treatment at home, or finding it inconvenient, or just forgetting, that requires attention.

Many reports dealt with acquired C1-inhibitor Deficiency. While rituximab is frequently employed for associated lymphoproliferative disease, there can be continued angioedema attacks. In one study, employing a new oral, bio available B-2 receptor antagonist given daily, symptoms were controlled for 8 weeks. Anti C1 INH can be present in many antibody classes and an unusually high incidence of erythema marginatum was reported which is more typically seen with hereditary rather than acquired disease. Patients with Monoclonal antibodies of unknown significance (MUGUS) had a 70% incidence of anti C1 INH. Tranexamic acid and lanadelumab were both effective treatment agents and evolution to lymphoma was 4% per patient-year. Absence of anti C1 INH can actually be due to all of it being incorporated into immune complexes, so it’s best to measure both free and bound antibody.

There was also a good representation of basic studies. Dr. Bork spoke to the bypass of factor XII and prekallikrein in HAE with mutated plasminogen or plasmin. The mutated protein directly cleaves kininogens to release bradykinin. Based on the literature it is likely that bradykinin is derived from LK as well as HK and with a 3:1 molar ratio favoring LK, LK may be the major source of bradykinin—a first in any HAE disorder. We learned that lactoferrin released from neutrophils is cleaved in the stomach to a smaller very positively charged peptide. It is antifibrinolytic and interferes with plasminogen and urokinase binding to cell receptors. It boosts IFNɤ production and affects T and B cell immunity to viruses including protection against COVID entrance into cells. C1 INH deficiency is associated with procoagulant measurements in plasma such as D-dimer, thrombin-antithrombin complexes (TAT), and prothrombin fragment 1 + 2. An increase in venous thrombosis is seen clinically, although the percentage is small. Patients have decreased numbers of NK cells, and the T cells are polarized toward Th2. C3 cleavage products are found indicating that complement activation goes beyond C1, C4, and C2. There is monocyte hyperactivity and increased B2 receptor expression on endothelial cells, but an elevated WBC seems to be due to decreased neutrophil adhesion to endothelial cells. Moringa Oleifara seed and extracts from it boosts Hep G 2 cells’ synthesis of C1 INH. An unusual type II mutation of C1 INH circulates disulfide linked to albumin which may relate to increased antigenic C1 INH seen in some with type II HAE. The vasodilatation due to bradykinin is in part due to modulation of claudin 5 and has no effect the glycocalyx. A positive response to lanadelumab reversed abnormalities of cleaved HK, and plasma levels of C4 and C1 INH. A need for a protease inhibitor cocktail and storage of plasma at -80o C was emphasized when drawing blood for bradykinin quantitation. Then it survives freeze-thawing. All components of the plasma contact activation bradykinin-forming cascade were shown to be activated by aggregated Aβ of Alzheimer’s disease; initiation requires factor XII activation and 25 µM zinc is required. The same abnormalities have, in the past few years, been observed in patients with some suggestion that the extent of activation is proportional to memory loss which suggests a trial of agents that block the bradykinin-forming cascade as therapy.

Finally there was attention to classification of the many types of angioedema. We learned about assisted diagnosis by artificial intelligence, and there is on-going updating of the International Consensus Document regarding the role of genetics in the diagnosis and management of the many new types of HAE. ACARE centers facilitate education and communication world-wide, and registries for rare disease help collect data regarding patients within individual countries.

## I-01 Lactoferrin—the alarmin which knows when is a time to kill and a time to heal

### Vladimir Leksa

#### Institute of Molecular Biology SAS, Bratislava, Slovakia

*Allergy, Asthma & Clinical Immunology* 2023, **19**(Suppl 1):I-01

Lactoferrin, a Member of the Lactotransferrin Family of Iron-Binding Glycoproteins, Is Present in Most Human Exocrine Fluids, Particularly Mother Milk. Both Human and Bovine Lactoferrin Exhibit a Plethora of Biological Activities, Including an Iron Sequestration, a Blockade of Proteases, Or a Direct Modulation of Immune Cells. Via These Activities Lactoferrin Plays Manifold Roles in Antimicrobial Host Defense. Furthermore, Antitumor Functions Have Also Been Attributed to Lactoferrin. The Ingested Lactoferrin Is Cleaved Upon Digestion in the Gastrointestinal Tract, Yielding Bioactive Peptides Called Lactoferricins and Lactoferrampins, Which Preserve and Even Augment Some Activities of the Intact Protein. Altogether, These Properties Make Lactoferrin a Cheap and Widely Available Candidate for Supplementary Therapy in Management of Infectious Diseases, Including COVID-19. Here, I Will Focus On the Role of Lactoferrin in Regulation of Pericellular Proteolysis and Discuss Its Possible Implication in Pathogenesis of Hereditary Angioedema.

## I-02 Hereditary Angioedema with the plasminogen gene mutation K330E and other types of Hereditary Angioedema with normal C1-INH

### Konrad Bork

#### Department of Dermatology, University Medical Center, Mainz, Germany

*Allergy, Asthma & Clinical Immunology* 2023, **19**(Suppl 1):I-02

Types of HAE include classical HAE due to the deficiency of functional C1-inhibitor (HAE-C1-INH), and various new types of HAE with normal activity of C1-INH (HAE with normal C1-INH, HAEnCI, HAE type III). By using next-generation sequencing techniques in more-generation families with patients with HAEnCI, various HAE-linked gene mutations were identified. Currently, 6 types of HAEnCI are recognized, based on underlying mutations of factor XII (HAE-FXII), angiopoietin-1 (HAE-ANGPT1), plasminogen (HAE-PLG, Hereditary Angioedema with the plasminogen gene mutation K330E), kininogen 1 (HAE-KNG1), myoferlin (HAE-MYOF), and heparan sulfate-glucosamine 3-O-sulfotransferase 6 (HAE-HS3ST6). In some of the families with HAEnCI patients, the genetic cause of HAEnCI is still unknown. The detection of the different genetic types of HAEnCI provides a more comprehensive insight into clinical and pathogenetic aspects of HAEnCI. Within the last years, a number of clinical differentiators were identified. They include a high frequency of tongue swellings in HAE-PLG which occur more frequently than in other types of HAEnCI. In some patients tongue swellings were the only type of swelling with no other clinical manifestation. Another differentiator is the higher prevalence of female compared to male mutation carriers in the most common types of HAEnCI. In HAE, less male and more female offspring of mutation carriers than expected for autosomal dominant inheritance inherited the familial mutation. In addition, there were less male offspring than expected in HAEnCI indicating selective effects during early embryonic development. In HAEnCI, the various mutation-induced protein changes may affect the kallikrein-kinin system (KKS), fibrinolytic system and other components of the complex mechanisms leading to angioedema formation. In HAE-PLG, there was a clinical response to a bradykinin B2 receptor antagonist as an indirect sign for bradykinin involvement. It was unknown whether the KKS pathway or another mechanism is linking the mutant protein to bradykinin overproduction. A recent study showed that plasminogen can directly release bradykinin from kininogens, and more efficiently if carrying the HAE mutation. In a multi-generation HAE-PLG family an additional F12 mutation, resulting in the loss of one F12 allele was identified. There were no differences in the clinical presentation between HAE-PLG patients with and without the additional F12 mutation. It was concluded that the KKS is bypassed in HAE-PLG.

## I-03 CRISPR opens the way to genetic therapy of angioedema

### Despina Sanoudou^1,2^

#### ^1^4th Department of Internal Medicine, “Attikon Hospital”, Medical School, National and Kapodistrian University of Athens, Greece; ^2^Biomedical Research Foundation of the Academy of Athens, Greece

*Allergy, Asthma & Clinical Immunology* 2023, **19**(Suppl 1):I-03

The vision of modifying the human genome to cure disease has been pursued since the 1980s. It is only recently however, that the fruits of these efforts started to reach clinical practice and transform patient lives. Among the few gene therapies that have obtained FDA approval are HEMGENIX for the treatment of adults with congenital Factor IX deficiency (Hemophilia B), LUXTURNA for the treatment of adults with retinal dystrophy caused by biallelic RPE65 mutations, ZYNGEGLO for the treatment of adults with beta-thalasemia, and ZOLGENSMA for treatment of children less than 2 years with spinal muscular atrophy caused by biallelic SMN1 mutations. These gene therapy approaches are largely based on reengineered viruses and have been focusing mostly on gene replacement.

The advent of the Clustered Regularly Interspaced Short Palindromic Repeats (CRISPR)/CRISPR-associated protein 9 (Cas9) genome editing technology has given the gene therapy field a major boost forward. CRISPR/Cas9 allows the accurate targeting of almost any desired genomic locus for the purpose of correcting disease-causing mutations or silencing genes associated with disease onset, promising safer and more effective therapies in the near future. Tens of thousands of CRISPR-related articles have been published, and the 2020 Nobel Prize in Chemistry was awarded to those who developed the CRISPR/Cas9 genome editing approach. Importantly, several CRISPR-based clinical trials are ongoing with highly encouraging results to date. Among them, the NTLA-2002 treatment against Hereditary Angioedema that is being developed by Intellia Therapeutics was approved by FDA for a Phase 2 clinical trial.

NTLA-2002 is designed to knock out the target gene kallikrein B1 (KLKB1) in hepatocytes. KLKB1 encodes prekallikrein, a precursor of plasma kallikrein, and therefore its knockout permanently reduces plasma kallikrein activity and halts the production of bradykinin to prevent HAE attacks. Preclinical and early clinical studies performed to date have demonstrated significant and sustained reduction in plasma kallikrein levels, as well as significant reduction or complete elimination of HAE attacks. Kallikrein inhibition is a clinically validated strategy for the preventive treatment of HAE attacks. However, current treatment options often include life-long therapies, which may require chronic intravenous (IV) or subcutaneous (SC) administration as often as twice per week, or daily oral administration to ensure constant pathway suppression for disease control. NTLA-2002 is the first single-dose investigational treatment in clinical trials for the potential to continuously reduce kallikrein activity and prevent attacks.

Notably, NTLA-2002 has been granted orphan drug designation for the treatment of Hereditary Angioedema (HAE). This paradigm-shifting treatment approach opens the way to a new era of Hereditary Angioedema therapies offering new hope to patients and their families.

## O-01 The Bradykinin cascade is activated in patients with Alzheimer’s disease and is activated in vitro by aggregated Aβ protein

### Allen P. Kaplan^1^, Berhane Ghebrehiwet^2^

#### ^1^The Medical University of South Carolina, Charleston, SC, USA; ^2^Department of Medicine, Stony Brook University, New York, NY, USA

*Allergy, Asthma & Clinical Immunology* 2023, **19**(Suppl 1):O-01

Alzheimer’s is a severe, common, intractable form of dementia for which adequate treatment is unavailable. Both anticholinergics and the recent monoclonal antibodies to the Plaque constituent Aβ protein have minimal effect on disease progression. We have demonstrated that while Aβ monomer is inactive, zinc-dependent aggregation produces a “surface” upon which autoactivation of factor XII can proceed followed by conversion of prekallikrein to kallikrein, and cleavage of HK to produce bradykinin. Aβ 1-42 > Aβ 1-40 > Aβ 1-39, in decreasing aggregability and potency. While reported in 1999, application of those results to patients has only recently been studied. Factor XIIa levels in blood and CSF elevate progressively as the disease worsens, cleaved HK levels correlate with clinical dementia and neuritic plaque scores, and an antibody to HK prevents its cleavage by Aβ, while an antibody that blocks binding of prekallikrein and factor XI to HK inhibits intrinsic coagulation induced by Aβ. Further plasma bradykinin levels in Alzheimer’s disease patients are elevated while reduced in CSF but binds to CSF Aβ40/ Aβ42 and co-localized with Aβ plaques in post-mortem brains. While similar findings have been reported in a mouse model of Alzheimer’s Disease. It is generally assumed that such activation is pathogenic for the disease and that its blockade would be beneficial. We have the drugs needed. Conversely one report of transgenic Alzheimer’s Disease mouse model found that B2 receptor agonists preserved memory and decreased plaque deposition. Nevertheless it is time to assess possible therapeutic agents for Alzheimer’s Disease that target individual steps of the intrinsic coagulation, bradykinin-forming cascade.

## O-02 C1-inhibitor deficiency is associated with a procoagulant phenotype in both humans and mice

### Steven P. Grover^1,*^, Tomohiro Kawano^1^, Jun Wan^1^, Rohan R. Kasthuri^1^, Sophia Dhrolia^1^, Zsofia Polai^2^, Omri Snir^3^, Sigrid Brækkan^3^, John-Bjarne Hansen^3^, Henriette Farkas^2^, Nigel Mackman^1^

#### ^1^UNC Blood Research Center, Division of Hematology, Department of Medicine, University of North Carolina at Chapel Hill, Chapel Hill, North Carolina, USA; ^2^Hungarian Angioedema Center of Reference and Excellence, Department of Internal Medicine and Haematology, Semmelweis University, Budapest, Hungary; ^3^Thrombosis Research Center, Department of Clinical Medicine, UiT—The Arctic University of Norway, Tromsø, Norway

##### ^*^Correspondence: steven_grover@med.unc.edu

*Allergy, Asthma & Clinical Immunology* 2023, **19**(Suppl 1):O-02

C1-inhibitor (C1-INH) represents the primary endogenous negative regulator of plasma kallikrein, activated factor XII and activated factor XI of the contact pathway of coagulation. C1-INH deficiency results in the rare episodic swelling disorder Hereditary Angioedema (HAE) that is driven by excessive kallikrein-mediated bradykinin generation. Patients with C1-INH deficiency associated Hereditary Angioedema (C1-INH-HAE) also have evidence of systemic activation of coagulation and a modest but significantly increased risk of venous thromboembolism.

We sought to evaluate the effect of C1-INH deficiency on coagulation in samples from patients with C1-INH-HAE and C1-INH deficient mice that model key aspects of HAE pathology.

Plasma from patients with a confirmed laboratory diagnosis of C1-INH-HAE had significantly increased contact pathway-initiated, but not extrinsic pathway-initiated, thrombin generation compared to matched controls (P < 0.05). This phenotype appeared to be primarily driven by enhanced contact pathway-initiated thrombin generation in plasmas from patients with severe C1-INH deficiency (< 25% of normal). C1-INH deficient mice (*C1-INH*^*−/−*^) had significantly increased plasma levels of coagulation markers prothrombin fragment 1 + 2 (P < 0.01) and thrombin antithrombin complexes (P < 0.001) compared to wildtype (*C1-INH*^+*/*+^) littermate controls. *C1-INH*^*−/−*^ mice also demonstrated significantly increased contact pathway-initiated whole blood thrombin generation (P < 0.05) compared to wildtype littermate controls. Further, *C1-INH*^*−/−*^ mice had significantly increased venous thrombosis in an inferior vena cava stenosis model (P < 0.05), but not arterial thrombosis in a carotid artery ferric chloride injury model, compared to wildtype littermate controls. Critically, enhanced whole blood thrombin generation and venous thrombosis in *C1-INH*^*−/−*^ mice was effectively reversed by intravenous administration of human purified C1-INH.

These findings indicate that C1-INH deficiency selectively enhances contact pathway-mediated activation of coagulation in both humans and mice. The enhanced venous thrombosis observed in C1-INH deficient mice complements the recently described phenotype in C1-INH-HAE. Further, C1-INH rescue experiments in mice highlight potential added benefits of C1-INH replacement therapy beyond management of swelling episodes.

## O-03 Th2 predominance and decreased NK cells in patients with Hereditary Angioedema—a connection with autoimmune disease?

### Linda Sundler Björkman^1,*^, Evelina Elmér^2^, Arne Egesten^1^, Lillemor Skattum^3^

#### ^1^Respiratory Medicine, Allergology & Palliative Medicine, Department of Clinical Sciences Lund, Lund University and Skåne University Hospital, Lund, Sweden; ^2^Department of Laboratory Medicine, Hematology and Transfusion Medicine, Lund University and Clinical Immunology and Transfusion Medicine, Region Skåne, Lund, Sweden; ^3^Department of Laboratory Medicine, Section of Microbiology, Immunology and Glycobiology, Lund University and Clinical Immunology and Transfusion Medicine, Region Skåne, Lund, Sweden 

##### ^*^Correspondence: linda.sundler_bjorkman@med.lu.se

*Allergy, Asthma & Clinical Immunology* 2023, **19**(Suppl 1):O-03

**Background:** Hereditary angioedema (HAE) is caused by mutations in the *SERPING1* gene, which lead to decreased levels or defective function of the C1 inhibitor (C1-INH). HAE patients display chronic activation of the classical pathway of complement, with low C4 levels. An increased risk of autoimmune disorders, particularly SLE, has been reported in HAE. This suggests that complement consumption affects adaptive immunity [1–3].

**Objective:** To investigate the distribution of lymphocyte subpopulations in relation to disease activity and degree of complement activation in a cohort of HAE patients (n = 16) compared to controls matched for age and sex (n = 16).

**Methods:** Lymphocyte populations of peripheral blood were characterized by flow cytometry. In addition, complement and complement fragments were measured.

**Results:** C4 and C1INH were lower in HAE patients than in controls. C4 was lower in HAE patients with high disease activity (HAE high, n = 7)) compared to HAE patients with low disease activity (HAE low, n = 9). C3d was higher among individuals with HAE compared to controls (*P* = 0.003), indicating increased activity of C3 convertases in HAE patients. C3d was also higher in HAE high compared to HAE low (*P* = 0.03). iC3b was higher in HAE patients compared to controls (*P* = 0.007).

NK cell counts were lower in patients compared to controls (*P* = 0.009). HAE high had a lower proportion of NK cells both compared to controls (*P* = 0.03) and compared to HAE low (*P* = 0.03). In patients, there was a correlation between the proportion of NK cells and C1INH levels (*P* = 0.03). In patients, the T helper cell balance was skewed towards more Th2 cells and less Th1 cells in comparison to controls. The proportion of Th2 effector memory T cells was higher in patients (*P* = 0.03) and the proportion of Th1 central memory T cells was lower in patients compared to controls (*P* = 0.03).

For the B cell subsets we found a higher proportion of transitional naïve B cells among HAE high compared to controls (*P* = 0.02).

**Conclusions:** In this study, HAE patients had lower NK cell counts and frequencies compared to controls. Low NK cells have previously been reported in several autoimmune diseases. In addition, the finding of a Th2-skewed T helper cell balance is interesting considering the increased risk of both autoimmunity and allergy associated with HAE.

**References**

1. Sundler Björkman L, Persson B, Aronsson D, Skattum L, Nordenfelt P, Egesten A. Comorbidities in Hereditary Angioedema-A population-based cohort study. Clin Transl Allergy. 2022 Mar;12(3):e12135.
2. Kessel A, Peri R, Perricone R, Guarino MD, Vadasz Z, Novak R, Haj T, Kivity S, Toubi E. The autoreactivity of B cells in Hereditary Angioedema due to C1-inhibitor deficiency. Clin Exp Immunol. 2012 Mar;167(3):422-8.
3. Triggianese P, Chimenti MS, Toubi E, Ballanti E, Guarino MD, Perricone C, Perricone R. The autoimmune side of Hereditary Angioedema: insights on the pathogenesis. Autoimmun Rev. 2015 Aug;14(8):665-9.

## O-04 Determining the effects of *Moringa Oleifera* on hepatic and monocytic cell lines in C1 esterase inhibitor production

### Asia Begum, Martin Gonzo*

#### University of Greenwich at Medway, Chatham Maritime, Kent, UK

##### ^*^Correspondence: martingnz@yahoo.co.uk

*Allergy, Asthma & Clinical Immunology* 2023, **19**(Suppl 1):O-04

Hereditary Angioedema (HAE) is an autosomal dominant disorder, caused by a C1 esterase inhibitor (C1-INH) deficiency or dysfunction. This rare disorder contributes to the reduced quality of life, considerable pain and debilitation and can be potentially fatal. Despite several therapeutic strategies for HAE, it remains a disease with substantial physical and economic burden for patients, with no actual cure and several adverse side effects posed from the current treatment options. The current treatments mainly focus on restoring the C1-INH but not upregulating its production. Therefore, the main aim of this study was to maximise the release of C1-INH by exposing the cells responsible for C1-INH secretion to *Moringa Oleifera* in attempt to upregulate its production and subsequent secretion to the extracellular space.

Initial studies investigated the level of secreted C1-INH by ELISA assay. HepG2 and THP-1 cells were stimulated by the addition to the culture medium of varying amounts of *Moringa Oleifera*. Culture supernatant samples were removed and the level of secreted C1-INH was determined. Crude *Moringa Oleifera* seed was extracted using the solvent extraction method. Cell viability was determined for HepG2 and THP-1 cells before and after treatment using the trypan blue exclusion method and MTT assay. The amount and location of C1-INH in HepG2 in the presence and absence of treatment was observed using florescence imaging. In this study, there was a significant difference observed after treatment with 1000 mg/L of *Moringa Oleifera* on HepG2 cells. There was a significant difference shown for both HepG2 and THP-1 cells per day compared with the control irrespective of the concentrations. MTT data confirmed that the HepG2 has a hepatoprotective role after exposure to *Moringa Oleifera*. Trypan blue exclusion method, showed a significant difference in cell viability for both cell lines after treatment with *Moringa Oleifera* compared with control. Overall, it can be concluded that HepG2 secretes a greater amount of C1-INH and for the first time *Moringa Oleifera* showed the maximum C1-INH secretion with maximum concentrations.

**Keywords**

Hereditary Angioedema; C1 esterase inhibitor; Moringa Oleifera; hepatic cells, monocytic cells

**Abbreviations**

American Type Culture Collection (ATCC); C1 esterase inhibitor (C1-INH); 4′,6-diamidino-2-phenylindole (DAPI); Dimethyl sulfoxide (DMSO); Eagle's Minimum Essential Medium (EMEM); Enzyme linked immunosorbent (ELISA); Ethylenediaminetetraacetic acid (EDTA); Fetal bovine serum (FBS); Hereditary Angioedema (HAE); Horseradish Peroxidase (HRP); Non-essential amino acids (NEAA); 3-(4,5-Dimethylthiazol-2-yl)-2,5-Diphenyltetrazolium Bromide (MTT); Phosphate-buffered saline (PBS); Streptavidin Conjugate (SABC); Serpin family G member 1 (SERPING1); Tetramethylbenzidine (TMB); Optical densities (OD)

## O-05 Purification and characterization of the C1-inhibitor R444C variant causing type 2 Hereditary Angioedema; covalent binding to human serum albumin and consequences

### Bence Farkas^1^, Péter Gál^1^, Lilian Varga^2^, Henriette Farkas^2^, József Dobó^1^

#### ^1^Institute of Enzymology, Research Centre for Natural Sciences, Budapest, Hungary; ^2^Hungarian Angioedema Center of Reference and Excellence, Department of Internal Medicine and Haematology, Semmelweis University, Budapest, Hungary

*Allergy, Asthma & Clinical Immunology* 2023, **19**(Suppl 1):O-05

Hereditary Angioedema (HAE) with C1-inhibitor (C1-INH) deficiency is caused by heterozygous mutations in the SERPING1 gene encoding C1-INH. HAE type I is characterized by low antigenic and low functional C1-INH levels, whereas HAE type II patients have often elevated antigenic, but still low functional C1-INH. A common variant causing HAE type II is the one bearing an R444C mutation (or R466C with precursor numbering). We have purified this variant from plasma of a patient by sequential chromatographic steps, and found that it co-purifies with a 66 kDa protein. On non-reducing gels a ~ 170 kDa band appears suggesting that C1-INH-R444C is covalently bound by disulfide bridging to albumin, which also contains an unpaired Cys residue. The ~ 170 kDa C1-inh-R444C-albumin complex seems to be the predominant variant in this patient, as in early chromatographic fractions, which do not discriminate between mutant and wild-type C1-inhibitor, more than 80% complex was observed. Further characterization and quantification of the variant form is underway. Nevertheless, complex formation with albumin might immediately suggest that the elevated level of antigenic C1-INH in patients carrying this mutation is caused by FcRn receptor mediated recycling.

## O-06 Cell–cell contacts and the glycocalyx are relevant structures in bradykinin-mediated endothelial barrier injury

### Robin Lochbaum, Angelina Gierke, Nevena Dimitrova, Anna Reich, Caroline Zimmermann, Thomas K. Hoffmann, Janina Hahn, Jens Greve

#### Department of Oto-Rhino-Laryngology, Head and Neck Surgery, Ulm University Medical Center, Ulm, Germany

*Allergy, Asthma & Clinical Immunology* 2023, **19**(Suppl 1):O-06

**Introduction:** In Hereditary Angioedema, a defect of the *SERPING1* gene results in reduced or defective formation of the C1 esterase inhibitor. This causes an increased formation of the tissue hormone bradykinin, which in turn leads to an increased water flow from the intravascular space into the interstitium. The exact underlying mechanism is not fully understood. However, the barrier function of the endothelium seems to play an important role. This is largely provided by the cell–cell contacts tight and adherens junctions, as well as the glycocalyx, a thin carbohydrate layer on the apical side of the endothelium. This project investigates the influence of bradykinin on these structures of the endothelial barrier.

**Material and methods:** We cultured human umbilical vein endothelial cells (HUVEC) as a commonly accepted endothelial cell model on transwell filters. Bradykinin was added and the changes on endothelial barrier function were investigated. For this purpose, transendothelial electrical resistance (TEER) and apparented permeability factor were determined, transendothelial water flux was measured using the D2O dilution method. Changes in the expression of genes and proteins of the tight and adherens junctions, as well as the glycocalyx, were examined by RT-PCR, western blot and immunocytochemistry. The thickness of the glycocalyx was determined by wheat germ agglutinin assay. To investigate the role of the glycocalyx on the effect of bradykinin in detail, it was previously enzymatically degraded in one group.

**Results:** Addition of bradykinin resulted in a decrease in TEER and an increase in permeability, consistent with disruption of the endothelial barrier. This was accompanied by increased transendothelial water flow. RT-PCR screening experiments showed modulation of cell–cell contact genes, particularly the tight junction protein claudin 5. In contrast, bradykinin itself had no significant effect on glycocalyx thickness in the wheat germ agglutinin assay. However, previous degradation of the glycocalyx resulted in an enhanced effect of bradykinin on the endothelial barrier.

**Discussion:** We demonstrated that bradykinin significantly damages the endothelial barrier. This was accompanied by a decreased expression of the tight junction protein claudin 5, which might explain the observed barrier damage. Furthermore, we demonstrated for the first time that the glycocalyx is protective against bradykinin-mediated barrier damage. This could provide further approaches for the understanding of angioedema.

## O-07 Monocytes hyperactivity and endothelial dysfunction in Hereditary Angioedema: the MONOBRAD study

### Nicolas Ozanne^1^, Jeremy Bellien^2,3^, Michèle Iacob^2^, Sylvanie Renet^3^, Sylvain Fraineau^3^, Nicolas Perzo^3^, Guillaume Armengol^1^

#### ^1^Department of Internal Medicine, CHU Rouen, Rouen, France; ^2^Department of Pharmacology, CHU Rouen, Rouen, France; ^3^University of Rouen Normandie, INSERM EnVI UMR 1096, Rouen, France

*Allergy, Asthma & Clinical Immunology* 2023, **19**(Suppl 1):O-07

**Introduction:** Hereditary Angioedema is a genetic disorder resulting in an accumulation of bradykinin causing attacks of angioedema, which can be life-threatening when involving upper airways. Some data suggested an endothelial dysfunction in this pathology that could contribute to increase cardiovascular risk, without identifying the physiopathological process involved.

**Objective:** This study aimed to assess whether endothelial dysfunction is present and whether the activity of bradykinin receptors on monocytes is modified apart from acute phases in patients with Hereditary Angioedema.

**Materials and methods:** Fifteen patients and fifteen control subjects matched for age, sex and cardiovascular risk factors were included in this transversal study. Peripheral and central blood pressure, carotid-to-femoral pulse wave velocity (PWV), carotid artery dilatolic diameter, intima-media thickness and distensibility, brachial artery endothelium-dependent flow- mediated dilatation (FMD) and glyceryl trinitrate (GTN)-induced endothelium-independent dilatation were evaluated. Blood samples were collected for determination of plasma proinflammatory cytokines (IL1, IL6 and TNFα) and oxidative stress (TBARs) and monocytes were isolated by a negative immunomagnetic selection for determination of mRNA and protein expression of bradykinin receptors B1 and B2 before and after stimulation with specific agonists of each receptor.

**Results:** Brachial FMD was reduced in patients (mean ± SD: 5.9 ± 2.2 vs. 7.4 ± 1.8, p = 0.04) without difference in GTN-induced dilatation, blood pressures, PWV, carotid artery parameters and plasma biomarkers. Basal mRNA expression was not different between groups but protein expression of B2 receptor was increased in patients compared with controls (median [IQR]: 0.033 [0.022; 0.057] vs 0.017 [0.008; 0.024] A.U., p < 0.01). In addition, change in B2 receptor expression induced by B1 activation (21.7 [2.6; 26.3] vs -6.9 [-36.3; 1.7] A.U., p = 0.026) was increased in patients and this increase was inversely correlated with the decrease in FMD (r^2^ = 0.77, p < 0.001).

**Conclusion:** Patients with Hereditary Angioedema display an increased B2 receptor expression and hyperactivity that could contribute to endothelial dysfunction. Whether these alterations contribute to increase cardiovascular risk remain has to be determined but these data suggest that blocking B2 receptors even apart from the acute phase of the disease may be beneficial.

## O-08 Biological pathway analyses of plasma proteomics in Hereditary Angioedema due to C1-inhibitor deficiency following lanadelumab treatment

### Dan Sexton^1^, Bin Li^1^, Dave Yeung^1^, Salomé Juethner^2^, Amanda MacDonald^1^, Anton Kichev^3^, Ezequiel Anokian^3^

#### ^1^Takeda Development Center Americas, Inc., Cambridge, MA, USA; ^2^Takeda Pharmaceuticals USA, Inc., Lexington, MA, USA; ^3^Clarivate, Barcelona, Spain

*Allergy, Asthma & Clinical Immunology* 2023, **19**(Suppl 1):O-08

**Rationale:** Comparison of plasma proteomic between healthy controls and patients with Hereditary Angioedema due to C1-inhibitor deficiency (HAE-C1-INH) may lead to identification of novel disease state biomarkers and provide additional insight into the mechanism of action of lanadelumab.

**Methods:** Proteomic analyses were performed using plasma from healthy controls (n = 30) and patients with HAE-C1-INH before (baseline, n = 125) and after 6 months of treatment with lanadelumab (300 mg every 2 weeks, n = 112) using a multiplex approach capable of comparing relative levels of > 7000 proteins using a technology based on DNA aptamers specific for each protein. Plasma samples for patients with HAE-C1-INH were collected in the phase III HELP study (NCT02586805) and from non-rollovers in the HELP open label extension study (NCT02741596).

**Results:** Relative plasma levels for several proteins were found to significantly differ between healthy controls and patients with HAE-C1-INH, and between matched baseline and post lanadelumab treatment in patients with HAE-C1-INH. As expected, plasma C1-inhibitor and complement C4 were significantly (P < 1.10e−39 false discovery rate [fdr], P < 6.6e−25 fdr, respectively) lower in patients with HAE-C1-INH at baseline than in healthy controls. Proteins associated with excess activation of the kallikrein-kinin system (KKS), including cleaved high molecular weight kininogen (cHMWK) were significantly higher in patients with HAE-C1-INH at baseline versus healthy controls (P < 6.7e−6 fdr). Furthermore, cHMWK levels were significantly lower in patients with HAE-C1-INH after receiving lanadelumab and not significantly different from those of healthy controls. Out of 1041 identified proteins that differed significantly in plasma from healthy controls and patients with HAE-C1-INH at baseline, 120 proteins were no longer different between healthy controls and patients with HAE-C1-INH after 6 months of treatment with lanadelumab. Cannonical pathway and local network analyses, conducted by comparing plasma protein levels in healthy control with that of patients with HAE-C1-INH before and after lanadelumab treatment, identifed potential disease state pathways and interconnected local networks.

**Conclusions:** Proteomic analyses of plasma from patients with HAE-C1-INH before and after treatment with lanadelumab compared with healthy controls may lead to discovery of novel protein biomarkers beyond KKS, provide insights on disease pathophysiology, and increase our understanding of lanadelumab mechanism of action.

This work was funded by Takeda Development Center Americas, Inc.

## O-09 An updated and comprehensive classification and terminology of angioedema

### Avner Reshef^1^, Thomas Buttgereit^2, 3^, Markus Magerl^2,3^, Marcus Maurer^2,3^ and the expert panel members of the DANCE initiative

#### ^1^Angioedema Research Center, Barzilai University Medical Center, Ashkelon, Israel; ^2^Angioedema Center of Reference and Excellence (ACARE), Institute of Allergology, Charité–Universitätsmedizin Berlin, Corporate Member of Freie Universität Berlin and Humboldt-Universität zu Berlin, Berlin, Germany; ^3^Fraunhofer Institute for Translational Medicine and Pharmacology ITMP, Immunology and Allergology, Berlin, Germany

##### *Correspondence: aresh@netvision.net.il

*Allergy, Asthma & Clinical Immunology* 2023, **19**(Suppl 1):O-09

**Background:** Angioedema (AE) manifests as transient tissue swelling due to increased vascular permeability [1]. It is linked to diverse entities, hereditary or acquired, with variable clinical manifestations and associated with different chemical mediators and vascular mechanisms [2]. Numerous classifications and terminologies are currently used, complicating the reporting of clinical studies, research collaborations, accurate diagnosis, and patient care. Recently, novel pathogenetic mechanisms and AE-driving mutations have been described [3], and certain medications in everyday use were also implicated with AE. Therefore, a modernized classification of AE is an unmet need. A new taxonomic system should profile all AE types and subtypes and make them suitable for future personalized medicine. For this purpose, a global initiative was conceived to reach a broad consensus on the definition, acronyms, nomenclature, and classification of AE (DANCE).

**Materials and Methods:** We performed a focused online literature search for definitions, acronyms, and classifications of AE. A steering committee (n = 13) debated and agreed on the updated classification and terminology's aims, rationale, and principles. Consented statements and a vocabulary of acronyms were presented to a large group of international AE experts, all accredited and experienced in allergy, immunology, and dermatology. Voting was performed by an online DELPHI process, in which a consensus was defined by reaching ≥ 75% agreement [4]. The DANCE initiative is supported and endorsed by several global organizations and professional societies.

**Results:** The global poll included 92 experts from 35 countries. It took five rounds of debates to reach a consensus on the statements and terminology by the steering committee members. The online DELPHI voting process by the global experts required three rounds over 16 months (June 2021 to November 2022). The agreement rate across all 19 statements ranged from 83 to 100%. The new classification comprises five subtypes of AE (namely: mast cell, bradykinin, vascular endothelium, drug-induced and unknown). Proposed endotypes had to combine clinical phenotypes with pathophysiology and recognized genetic mutations. The new proposal also includes a revised list of acronyms covering all AEs.

**Conclusions:** The global initiative resulted in an international consensus on the classification and terminology of a wide range of AE entities. The new taxonomy is meant to harmonize and facilitate AE research, accurate diagnosis, and better patient care.

We are indebted to all the international voting experts who shared their suggestions with us and for the excellent secretarial work of Ms. Rebekka Locke, the ACARE coordinator.

**References**

1. Claesson-Welsh L, Dejana E, McDonald DM. Permeability of the Endothelial Barrier: Identifying and Reconciling Controversies. Trends Mol Med. 2021;27(4):314-331
2. Maurer M, Magerl M. Differences and Similarities in the Mechanisms and Clinical Expression of Bradykinin-Mediated vs. Mast Cell-Mediated Angioedema. Clin Rev Allergy Immunol. 2021;61(1):40-49
3. Veronez CL, Csuka D, Sheikh FR, Zuraw BL, Farkas H, Bork K.The Expanding Spectrum of Mutations in Hereditary Angioedema. J Allergy Clin Immunol Pract. 2021;9(6):2229-2234
4. Diamond IR, Grant RC, Feldman BM, Pencharz PB, Ling SC, Moore AM, et al. Defining consensus: A systematic review recommends methodologic criteria for reporting of Delphi studies. J Clin Epidemiol. 2014;67:401-409.

## O-10 Detection of Bradykinin and its Major Metabolites by Liquid Chromatography Tandem Mass Spectrometry (LC–MS/MS)

### Yunkou Wu^1^, Lili Wan^1,2^, Kusumam Joseph^1^, Joseph Chiao^1^, H. Henry Li^1,2,^*

#### ^1^Virant Diagnostics, Wheaton, MD, USA; ^2^Institute for Asthma and Allergy, Wheaton, MD, USA

##### *Correspondence: henryli@allergyasthma.us

*Allergy, Asthma & Clinical Immunology* 2023, **19**(Suppl 1):O-10

**Background:** The nine amino acid peptide Bradykinin (BK)1–9, generated from cleavage from High-molecular-weight kininogen (HMWK) upon activation of contact system, is considered the key mediator in the pathogenesis of Hereditary Angioedema (HAE) and a significant proportion of idiopathic angioedema. BK1-9 is metabolized by angiotensin converting enzyme (ACE) and other peptidases to generate its metabolites, BK1-8, 1–7, 1–5, and 2–9. Accurate measurement of bradykinin and its major metabolites can significantly improve the clinical assessment of angioedema, from diagnosis to therapeutic response.

**Materials and methods:** An LC–MS/MS method for quantification of BK1-9, BK1-8, BK1-7, BK1-5, and BK2-9 concentration was established with LLOQ of 0.1 ng/mL for all analytes. Blood samples were collected prospectively from 36 subjects with or without the diagnosis of HAE were obtained from the Institute for Asthma and Allergy using a clinical study protocol approved by a central IRB. To prevent the ex vivo production of bradykinin 1–9 and its metabolites, blood samples were immediately transferred to chilled ethanol or proteinase inhibitor cocktail containing tubes. Non-treated serum and plasma samples were used as controls. The LC–MS samples were prepared with techniques of protein precipitation and then solid phase extraction.

**Results:** BK1-5 and BK1-8 were two of the most abundant metabolites and were quantifiable in all 36 subjects. BK1-9 levels are low in normal subjects and in HAE patients although the levels increase considerably during an attack. Both ethanol and proteinase inhibitor treated samples demonstrate dramatically lower levels of detectable bradykinin and its metabolites. Using ethanol or proteinase inhibitors during blood collection are critical to prevent the ex vivo generation of bradykinin. Plasma samples had lower levels of bradykinin than those of serum samples. Importantly, HAE patients on long term prophylaxis have much lower baseline bradykinin levels than patients on acute treatment only. Baseline levels of bradykinin metabolites in many HAE patients who are on long term prophylaxis are close to normal subjects.

**Conclusion:** Accurate determination of physiologically relevant levels of BK and its metabolites can be achieved by proper sample handling. The finding of this research offers a better understanding of metabolic profile of bradykinin in HAE patients as well as in healthy subjects, indicating LC–MS/MS would be a very useful tool for diagnosis and for monitoring therapeutic response.

## O-11 Diagnosis of angioedema by Artificial Intelligence

### Felix Aulenbacher^1,2^, Henriette Farkas^3^, Kinga Viktória Kőhalmi^3^, Emek Kocatürk^1,2,4^, Emel Aygören-Pürsün^5^, Ludovic Martin^6^, Hilary Longhurst^7^, Petra Staubach^8^, Andrea Zanichelli^9^, Werner Aberer^10^, Anette Bygum^11^, Mignon van den Elzen^12^, Janne Björkander^13^, Marcus Maurer^1,2^, Markus Magerl^1,2^

#### ^1^Angioedema Center of Reference and Excellence (ACARE), Institute of Allergology, Charité—Universitätsmedizin Berlin, corporate member of Freie Universität Berlin and Humboldt-Universität zu Berlin, Berlin, Germany; ^2^Fraunhofer Institute for Translational Medicine and Pharmacology ITMP, Allergology and Immunology, Berlin, Germany; ^3^Hungarian Angioedema Center of Reference and Excellence, Department of Internal Medicine and Haematology, Semmelweis University, Budapest, Hungary; ^4^Koç University School of Medicine, Department of Dermatology, Istanbul, Turkey; ^5^Angioedema Center, Pediatric Clinic, University Hospital Frankfurt, Frankfurt, Germany; ^6^Department of Dermatology, Angers University Hospital, Angers, France; ^7^Department of Immunology, Auckland City Hospital and Department of Medicine, University of Auckland, Auckland, New Zealand; ^8^Department of Dermatology, University Medical Center Mainz, Mainz, Germany; ^9^Department of Biomedical and Clinical Sciences “Luigi Sacco,” Università degli Studi di Milano, Milan, Italy; ^10^Department of Dermatology, Medical University of Graz, Graz, Austria; ^11^Clinical Institute, University of Southern Denmark, Odense, Denmark; ^12^Department of Dermatology/Allergology, University Medical Center Utrecht, Utrecht University, Utrecht, The Netherlands; ^13^Wetterhälsan Outpatient Clinic, Jönköping and Futurum, Academy of Health and Care, Jönköping, Sweden

*Allergy, Asthma & Clinical Immunology* 2023, **19**(Suppl 1):O-11

**Background:** The correct diagnosis of recurrent angioedema (RAE) is a major challenge, mainly because there are no reliable and readily accessible biomarkers for most types. Often, the diagnosis is based on the medical history, clinical presentation, response or non-response to various drugs, in combination with other features such as family history. Here, we used machine learning (ML) to diagnose RAE types and subtypes.

**Materials and methods:** A comprehensive literature search was performed to identify clinical features that are typical or atypical for six different RAE types, e.g. mast cell-mediated RAE/chronic urticaria (RAE-CU), bradykinin-mediated RAE including HAE with and without deficiency of C1-inhibitor, RAE due to acquired C1-inhibitor deficiency, ACE inhibitor-induced RAE, and idiopathic RAE. From this, questions were developed and included in a questionnaire that was distributed to patients treated at angioedema clinics in Angers, Berlin, Budapest, Istanbul, London and Mainz. The information obtained from individual patients (n = 342) was matched with the diagnosis established by their treating physician.

An R-script was developed to test various ML-models on patient data using the package “caret”. To enable accurate validation, the patient data were divided into training and test datasets, and the model results were verified through the medical diagnosis. The model with the highest accuracy and Kappa value (random forest—RF) was subsequently optimized through hyperparameter tuning to further improve prediction accuracy.

**Results:** Density plots were used to weigh the importance of answers across ten questions asked. The final modified ML RF model demonstrated a high degree of agreement between the disease types diagnosed by physicians and ML. Across the six RAE types, an accuracy of 89.2% and a Kappa value of 81.8% were achieved. A very high sensitivity (96% & 100%) and specificity (89% & 94%) were obtained for RAE/CU and HAE type 1&2, respectively. The sensitivity of ML-based diagnosis was linked to the number of patients per RAE type, i.e. it was lower for types with fewer patients affected.

**Conclusions:** Based on the answers of RAE patients to 10 questions, ML succeeded in assigning signature patterns to 6 types of angioedema and diagnosed RAE types with high agreement to the diagnoses made by experts. Optimization by self-learning improved accuracy. Further training of the ML algorithm can be expected to further increase diagnostic accuracy for less frequent types of RAE.

## O-12 *SERPING1* splicing-affecting variants highly represented in the Czech cohort of HAE-1/HAE-2 patients

### Hana Grombirikova^1,2^, Viktor Bily^1,2^, Premysl Soucek^1,2^, Michal Kramarek^1,2^, Roman Hakl^2,3^, Lucie Ballonova^1,2^, Dita Ricna^1^, Marta Sobotkova^4^, Radana Zachova^4^, Pavel Kuklinek^2,3^, Pavlina Kralickova^5^, Irena Krcmova^5^, Jana Hanzliková^6^, Martina Vachova^6,7^, Olga Krystufkova^8^, Eva Dankova^9^, Miloš Ješenak^10^, Jiří Litzman^2,3^, Tomas Freiberger^1,2^

#### ^1^Centre for Cardiovascular Surgery and Transplantation, Brno, Czech Republic; ^2^Faculty of Medicine, Masaryk University, Brno, Czech Republic; ^3^Department of Allergology and Clinical Immunology, St. Anne’s University Hospital in Brno, Czech Republic; ^4^Department of Immunology, 2^nd^ Medical School Charles University and University Hospital Motol, Prague, Czech Republic; ^5^Institute of Clinical Immunology and Allergy, University Hospital Hradec Kralove, Charles University, Faculty of Medicine in Hradec Kralove, Hradec Kralove, Czech Republic; ^6^Department of Immunology and Allergology, University Hospital Pilsen, Czech Republic; ^7^Department of Immunology and Allergology, Faculty of Medicine in Pilsen, Charles University, Czech Republic; ^8^Institute of Rheumatology and Department of Rheumatology, 1^st^ Faculty of Medicine, Charles University, Prague, Czech Republic; ^9^Immunia, Prague, Czech Republic; ^10^National Centre for Hereditary Angioedema, Department of Pediatrics, Department of Pulmonology and Pathophysiology, Department of Clinical Immunology and Allergology, Comenius University in Bratislava, Jessenius Faculty of Medicine, University Teaching Hospital in Martin, Slovakia

*Allergy, Asthma & Clinical Immunology* 2023, **19**(Suppl 1):O-12

In general, splicing-affecting variants are responsible for 15–50% of Mendelian disorders. Variants located in the acceptor or donor splice site regions mostly impair pre-mRNA splicing when disturbing directly the conserved dinucleotide sequence AG or GT. The effect of other sequence alterations (e.g. those situated in the polypyrimidine tract, inside exons or deeply in introns) is much more difficult to assess. Multiple factors influence determination of the splice site strength, such as intronic AG-dependence, the quality of polypyrimidine tract or the presence of splicing regulatory elements in the neighbouring sequences. This kind of complex regulation makes the predictions of splicing affection very difficult and testing patient’s mRNA expression and/or functional analyses are often necessary to prove suspected variant’s pathogenicity.

In the Czech national HAE cohort comprising 88 families, we were able to detect a causal *SERPING1* defect in all but one. Splicing-affecting *SERPING1* variants accounted for 28% of all detected 58 unique variants, which is a much higher proportion than reported from the LOVD database (14%). Out of 16 splicing variants, only 7 affected canonical splice sites directly; more specifically there were 4 single nucleotide substitutions, 1 single nucleotide deletion and 2 large deletions (44 bp and 412 bp). Remaining 9 variants included 2 substitutions of the last exonic nucleotide, 5 single nucleotide intronic substitutions located at positions -12, -7, + 3, and + 5 (2x), 1 deletion spreading from -19 to -4 position, and 1 deep intronic variant at position + 384.

Most of the molecular genetic causes of HAE are being determined by routinely used approaches such as direct sequencing of *SERPING1* exons, exon/intron boundaries, as well as determining the *SERPING1* exon copy number variation, frequently using targeted NGS panels nowadays. In this study, we demonstrated that a combined strategy of sequencing, extended to UTR and deep intronic regions, using advanced in silico prediction tools, assessing patients’ mRNA and applying functional minigene assays might considerably increase our capacity to identify and characterise disease causing *SERPING1* splicing-affecting variants.

The study was supported by grant number NV18-05–00330 from the Ministry of Health of the Czech Republic, and Specific University Research Grant number MUNI/A/1244/2021 provided by the Ministry of Education, Youth and Sports of the Czech Republic.

## O-13 HAErmony-1: Clinical study of adeno associated virus vector-mediated gene therapy of human C1-inhibitor in Hereditary Angioedema Type I and II

### Marc Riedl^1,^*, Jonathan A. Bernstein^2^, Thomas Machnig^3^, Jack Brownrigg^3^, Iris Chen^4^, L. Mason Shih^4^, M. Benjamin Hock^4^, H. James Wedner^5^

#### ^1^Division of Allergy & Immunology, University of California, San Diego, CA, USA; ^2^Division of Allergy & Immunology, University of Cincinnati, Cincinnati, OH, USA; ^3^BioMarin (UK) Ltd., London, UK; ^4^BioMarin Pharmaceutical Inc., Novato, CA, USA; ^5^Division of Allergy & Immunology, Washington University, St. Louis, MO, USA

##### *Correspondence: mriedl@health.ucsd.edu

*Allergy, Asthma & Clinical Immunology* 2023, **19**(Suppl 1):O-13

**Introduction:** Hereditary Angioedema (HAE) type I and II are caused by mutations in *the SERPING1* gene that lead to low functional levels of the highly expressed serum protein C1 esterase inhibitor (C1-INH). Current approaches to prevent HAE attacks include long-term prophylaxis using agents that chronically inhibit uncontrolled plasma kallikrein generation or continuously replace the missing or dysfunctional C1-INH protein. BioMarin has developed BMN 331, an adeno-associated virus (AAV) serotype 5 vector containing an expression cassette that encodes for the human C1-INH protein. We are investigating whether a single infusion of BMN 331 can result in expression of functional C1-INH protein and provide long-term correction of C1-INH deficiency.

**Methods:** HAErmony-1 (331-201) is a first-in-human, phase I/II, open-label, dose-escalation study to determine the safety, tolerability, and preliminary short- and long-term efficacy of a single IV infusion of BMN 331 in people with HAE due to C1-INH deficiency (Type I or II). A study schema is shown in Fig. 1. An independent Data Monitoring Committee (DMC) will assess safety, oversee study conduct, and provide recommendations on dose-escalation and expansion for each of the planned dose cohorts based on safety evaluations in conjunction with C1-INH functional plasma protein levels and HAE attack occurrences. The study is currently enrolling in the USA, and additional study sites are planned in the EU and Australia.

**Figure 1 (abstract O-13):**
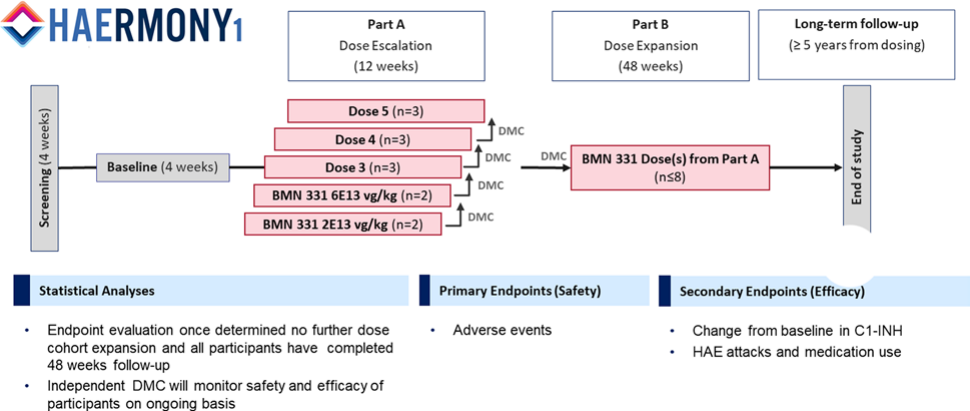
Study schema

**Results:** The first study participant received BMN 331 in April 2022 at Washington University in St. Louis, MO, USA. As of December 2022, a total of three participants have received BMN 331: two at a dose of 2E13 vg/kg and one at a dose of 6E13 vg/kg. Infusions of BMN 331 were well tolerated, and no serious or severe (Grade ≥ 3) adverse events were observed following treatment. An increase in antigenic C1-INH protein levels was observed starting 4 weeks after the infusion of the 6E13 vg/kg dose, and C1-INH levels approached the lower limit of normal (normal range of C1-INH antigen: 19–39 mg/dL) at 8 weeks post infusion.

**Conclusions:** HAErmony-1 is the first gene transfer study in people with HAE. The study will evaluate the potential of BMN 331 to provide safe and durable therapeutic benefits by restoring C1-INH levels, preventing HAE attacks and thereby reducing treatment burden on patients. For more information about this ongoing study, please see https://haegenestudy.com/haermony-1-study/.

**Ethics Approval:** The study was approved by local/ central IRB at all enrolling study sites.

**Trial registration**

Current controlled trials (NCT 05121376, A Gene Therapy Study of BMN 331 in Subjects With Hereditary Angioedema—Full Text View—ClinicalTrials.gov)

## O-14 Hereditary Angioedema variant curation using a ClinGen framework

### Matija Rijavec^1^, Roshini S. Abraham^2^, Marwa Elnagheeb^3^, Jerneja Debeljak^1^, Hana Grombirikova^4^, Dhanya Lakshmi Narayanan^5,6^, Madelynne Manansala^7^, Shruthi Mohan^3^, Teagan Nunnery^3^, Amber Stafford^3^, Dorottya Csuka^8^, Alberto López-Lera^9^, Tomáš Freiberger^4^, Anastasios E. Germenis^10,11^, Gaelle Hardy^12^, Camila L. Veronez^13^, João Bosco Pesquero^14^, Marc Riedl^15^, Ágnes Szilágyi^8^, Raffi Tachdjian^16^, Bruce Zuraw^17^, Christian Drouet^18^

#### ^1^University Clinic of Respiratory and Allergic Diseases Golnik, Golnik, Slovenia; ^2^Department of Pathology and Laboratory Medicine, Nationwide Children's Hospital, Columbus, OH, USA; ^3^University of North Carolina, Chapel Hill, NC, USA; ^4^Molecular Genetics Laboratory, Centre for Cardiovascular Surgery and Transplantation, Brno and Medical Faculty, Masaryk University, Brno, Czech Republic; ^5^Department of Medical Genetics, Kasturba Medical College, Manipal, Manipal Academy of Higher Education, Manipal, India; ^6^DBT Wellcome Trust India Alliance, Early Career Clinical and Public Health Research Fellow; ^7^Invitae, San Francisco, CA, USA; ^8^Department of Internal Medicine and Haematology, Hungarian Angioedema Center of Reference and Excellence, Semmelweis University, Budapest, Hungary; ^9^CIBERER U-754, Hospital La Paz Institute for Health Research (IdiPAZ), Madrid, Spain; ^10^CeMIA SA, Larissa, Greece; ^11^School of Health Sciences, Faculty of Medicine, University of Thessaly, Larissa, Greece; ^12^Molecular Genetics Laboratory, Grenoble Alpes University Hospital, Grenoble, France; ^13^Sanofi, São Paulo, Brazil; ^14^Centre for Research and Genetic Diagnosis of Genetic Diseases - Department of Biophysics, Federal University of São Paolo, São Paolo, Brazil; ^15^Division of Rheumatology, Allergy and Immunology, University of California San Diego, La Jolla, CA, USA; ^16^Division of Allergy, Immunology and Rheumatology, David Geffen School of Medicine, University of California Los Angeles, Los Angeles, CA, USA; ^17^University of California, San Diego, San Diego, CA, USA; ^18^Institut Cochin, INSERM UMR1016, Université Paris Cité, Paris, France

*Allergy, Asthma & Clinical Immunology* 2023, **19**(Suppl 1):O-14

The Hereditary Angioedema Variant Curation Expert Panel (HAE VCEP) was assembled in 2022, and affiliated to the Immunology Clinical Domain Working Group, as part of the Clinical Genome Resource (ClinGen), a NIH-funded program to create a publicly available resource that defines the relevance of genes and variants for use in precision medicine available for laboratories, clinicians, and scientists. Furthermore, the identification of a variant in families makes easier its distribution within family members and detection of rare conditions, e.g. de novo situation, parental disomy.

The objective of HAE VCEP, composed of an international, multidisciplinary group of experts and curators, is to evaluate and classify the pathogenicity of variants in genes responsible for Hereditary Angioedema (HAE). The VCEP proposes to develop HAE-specific classification rules based on gene- and disease-specific modifications of the ACMG/AMP standards and guidelines for interpreting sequence variants [1, 2]. In the next step, specific rules will be validated and, if needed, refined. Before final VCEP approval, plans for ongoing variant review, reanalysis and discrepancy resolution will be defined.

The *SERPING1* gene-disease relationship was curated by the Antibody Deficiencies GCEP (*SERPING1* curation results (*clinicalgenome.org*)) prior to the initiation of the HAE VCEP’s variant curation. The HAE VCEP will start with an extension of the curation of variants in C1-INH (*SERPING1*), the gene responsible for Hereditary Angioedema due to C1-inhibitor deficiency (C1-INH-HAE; OMIM: 106100). More than 800 variants in the *SERPING1* gene have been found in the literature [3], but less than half are reported in the ClinVar.

The group will then develop pathogenicity classification rules to curate variants in genes responsible for Hereditary Angioedema with normal C1-INH (nl-C1-INH-HAE), such as *F12*, *PLG*, *ANGPT1*, *KNG1*, *MYOF*, and *HS3ST6* (OMIM: 610618, 619360, 619361, 619363, 619366, 619367). Only genes with strong or definitive evidence that variation causes Hereditary Angioedema will be selected. Variant interpretations will be publicly available in the ClinGen Evidence Repository and in ClinVar.

**References**

1. Richards S, et al. Standards and guidelines for the interpretation of sequence variants: a joint consensus recommendation of the American College of Medical Genetics and Genomics and the Association for Molecular Pathology. Genet Med. 2015; 17:405-424.
2. Germenis AE, et al. International Consensus on the Use of Genetics in the Management of Hereditary Angioedema. J Allergy Clin Immunol Pract. 2020; 8:901-911.
3. Drouet C, et al. SERPING1 Variants and C1-INH Biological Function: A Close Relationship With C1-INH-HAE. Front Allergy. 2022; 3:835503.

## RT-1 International consensus on the use of genetics in the management of Hereditary Angioedema—The 2023 revision and update

### Anastasios E. Germenis^1,^*, João Bosco Pesquero^2^, Sven Cichon^3^, Dorottya Csuka^4^, Christian Drouet^5^, Henriette Farkas^4^, Tomáš Freiberger^6^, Stephen Jolles^7^, Camila Lopes Veronez^8^, Alberto López Lera^9^, Margarita López Trascasa^9^, Maurizio Margaglione^10^, Matija Rijavec^11^, Ágnes Szilágyi^4^, Maria Zamanakou^12^ on behalf of the Hereditary Angioedema International Working Group (HAWK)

#### ^1^Department of Immunology & Histocompatibility, School of Health Sciences, Faculty of Medicine, University of Thessaly, Larissa, Greece; ^2^Department of Biophysics, Universidade Federal de São Paulo, São Paulo, Brazil; ^3^Department of Biomedicine, Institute of Medical Genetics and Pathology, University Hospital Basel, University of Basel, Basel, Switzerland; ^4^Hungarian Angioedema Center, 3^rd^ Department of Internal Medicine, Semmelweis University, Budapest, Hungary; ^5^Institut Cochin, INSERM UMR1016, Université Paris Cité, Paris, France; ^6^Molecular Genetics Laboratory, Centre for Cardiovascular Surgery and Transplantation, Brno and Medical Faculty, Masaryk University, Brno, Czech Republic; ^7^Immunodeficiency Centre for Wales, University Hospital of Wales, Cardiff, UK; ^8^Sanofi, São Paulo, Brazil; ^9^Hospital La Paz Health Research Institute -IdiPAZ, Departamento de Medicina, Universidad Autónoma de Madrid, Madrid, Spain; ^10^Medical Genetics, Department of Clinical and Experimental Medicine, University of Foggia, Foggia, Italy; ^11^Laboratory for Clinical Immunology and Molecular Genetics, University Clinic of Respiratory and Allergic Diseases Golnick, Golnik, Slovenia; ^12^CeMIA SA, Larissa, Greece

##### *Correspondence: agermen@med.uth.gr

All authors equally contributed to this work.

*Allergy, Asthma & Clinical Immunology* 2023, **19**(Suppl 1):RT-1

During the 11th C1-inhibitor Deficiency and Angioedema Workshop held in Budapest, in May 2019, an open meeting took place and, using a modified Delphi survey, a consensus was developed on the use of genetics in the management of Hereditary Angioedema (HAE) [1]. Since then, a great progress of special interest for the clinical management of the disease has been made in angioedema genetics. The main discoveries refer to the detection of a lot of new *SERPING1* variants (including deep intronic ones) associated with HAE due to C1-inhibitor (C1-INH) deficiency as well as of a series of new genes, variants of which are responsible for HAE with normal C1-INH. These findings necessitated an organized effort towards the evaluation and classification of the pathogenicity of variants in genes responsible for HAE. As a result, a Hereditary Angioedema Variant Curation Expert Panel (HAE VCEP) has been assembled as part of the publicly available Clinical Genome Resource (ClinGen), a NIH-funded project. These advances must be considered under the light of the expanding use of genomic technologies and the promising perspectives of gene therapy.

To this end, the same as in 2019 international multidisciplinary group of experts was convened expanded with some new members, with the objective to revise the statements of the 2019 consensus. The revised statements were distributed to all HAWK members as well as to the colleagues who were the co-authors of all publications of the last 3 years on angioedema genetics, for their feedback. The returned comments will be presented and discussed with the participants of the 13th C1-inhibitor Deficiency and Angioedema Workshop in order a revised consensus to be developed using again a modified Delphi survey via voting. The revised statements are expected both to guide clinicians and to serve as a framework for future educational and further genetic testing developments as the field of angioedema genetics continues to evolve rapidly.

**Reference**

1. Germenis AE, et al. International consensus on the use of genetics in the management of hereditary angioedema. J. Allergy Clin Immunol Pract 2020;8:901-911

## O-15 Attack-free status across subgroups of patients with Hereditary Angioedema (HAE) after 96 weeks of berotralstat treatment: results from the APeX-S trial

### Avner Reshef^1^, Heidi Zafra^2^, Douglas T. Johnston^3^, Dianne Tomita^3^, Bhavisha Desai^3^, Emel Aygören-Pürsün^4^

#### ^1^Allergy, Immunology and Angioedema Center, Barzilai University Hospital, Ashkelon, Israel; ^2^Division of Allergy/Clinical Immunology, Medical College of Wisconsin, Milwaukee, WI, USA; ^3^BioCryst Pharmaceuticals, Inc., Durham, NC, USA; ^4^University Hospital Frankfurt, Goethe University, Frankfurt, Germany

*Allergy, Asthma & Clinical Immunology* 2023, **19(Suppl 1): **O-15

**Background:** The goal of long-term Hereditary Angioedema (HAE) prophylaxis is to reduce overall burden of disease by lowering the frequency and severity of attacks [1]. Berotralstat is a first-line, once daily (QD) oral plasma kallikrein inhibitor, indicated for prophylactic treatment for HAE. Long-term safety and effectiveness of berotralstat through 96 weeks was previously reported for all patients receiving 150 mg in APeX-S study [2]. Here we report the number of attack-free days through 96 weeks, in patients receiving berotralstat 150 mg in the APeX-S trial, stratified by baseline age, gender, and prior prophylaxis.

**Materials and methods:** In the APeX-S trial (NCT03472040), eligible patients with Type 1 or 2 HAE were allocated to open-label berotralstat 110 mg or 150 mg QD until superior efficacy at 150 mg was demonstrated in the APeX-2 trial (NCT03485911). This analysis evaluated attack-free status in patients receiving berotralstat 150 mg in APeX-S, an open-label study assessing the long-term safety (primary objective) and effectiveness (secondary objective) of berotralstat. Patients were stratified by baseline age, gender, and prior prophylaxis. Attack-free days were calculated by subtracting the number of days with angioedema symptoms from the duration of the reporting period of interest for each patient.

**Results:** Overall, 287 patients received berotralstat 150 mg for the study duration. Patients who received berotralstat 150 mg in an open-label fashion remained attack-free a total of 94% of days (100,161/106,926) during the 96-week period. When stratified by baseline characteristics, attack-free status was consistently high regardless of age (12–17 years, 97% [7,885/8,166]; 18–64 years, 93% [88,184/94,573]; ≥ 65 years, 98% [4,092/4,187]) and gender (female, 94% [60,973/65,169]; male, 94% [39,188/41,757]). Similar results were seen regardless of prior HAE prophylaxis treatment (prior androgens, 92% [52,209/56,611]; prior C1-inhibitor, 92% [31,512/34,425]). The most common treatment-emergent adverse events (≥ 10% of patients receiving berotralstat 150 mg) were nasopharyngitis (20.6%), diarrhoea (14.6%), upper respiratory tract infection (12.5%), headache (11.8%), and abdominal pain (10.1%), which are consistent with previous reports.

**Conclusions:** The percentage of attack-free days remained consistently high with berotralstat monotherapy through 96 weeks, irrespective of baseline characteristics, suggesting a durable treatment effect and sustained reduction in disease burden.

**References**

1. Craig T, et al. Long-term prophylaxis therapy in patients with Hereditary Angioedema with C1-inhibitor deficiency. Ann Allergy Asthma Immunol. 2018 Dec;121(6):673-679.
2. Aygören-Pürsün E, et al. Long-term HAE Prophylaxis with Berotralstat is Well Tolerated and Effective: Analysis for the APeX-S Study. Presented at The American Academy of Allergy, Asthma & Immunology; February 24-27, 2023; San Antonio, TX.

## O-16 Rationale for the short-term prophylaxis regimen with sebetralstat in KONFIDENT-S

### Matthew Iverson, Edward Duckworth, Erik Hansen, Sally L. Hampton, Michael D. Smith, Paul K. Audhya, Christopher M. Yea

#### KalVista Pharmaceuticals, Salisbury, UK, and Cambridge, MA, USA

*Allergy, Asthma & Clinical Immunology* 2023, **19**(Suppl 1):O-16

**Background:** For patients with Hereditary Angioedema (HAE), guidelines recommend short-term prophylaxis (STP) before medical or dental procedures. However, recommended STP treatments require parenteral administration, which presents challenges with preparation, venous access, and injection-site pain. Sebetralstat is an investigational oral plasma kallikrein (PKa) inhibitor for the on-demand treatment of HAE attacks. To support the rationale for the STP regimen in KONFIDENT-S, we report pharmacokinetic (PK), pharmacodynamic (PD), and safety data from a phase 1 trial which evaluated 3 doses of sebetralstat every 8 h (q8h) compared with dosing every 2 h (q2h) or 4 h (q4h).

**Methods:** Healthy volunteers were assigned to 3 cohorts with q8h, q4h, or q2h dosing schedules and then randomised to receive 3 × 600 mg sebetralstat or placebo while fasting. Venous blood was collected for PK measurements at prespecified intervals following the first and third doses, up to 40 h post-dose. In an exploratory PD analysis, PKa enzyme activity was assayed ex vivo to measure inhibition of exogenously activated enzyme. Safety was assessed. Results were analysed descriptively.

**Results:** The geometric mean C_max_ of dose 1 and dose 3 was 3916 ng/mL and 8838 ng/mL, respectively, in the q8h cohort (n = 6), 4412 ng/mL and 7136 ng/mL, respectively, in the q4h cohort (n = 6), and 5035 ng/mL and 15,627 ng/mL, respectively, in the q2h cohort (n = 18). The lowest arithmetic mean plasma concentrations in the q8h cohort were 758.5 ng/mL at 8 h, 749.8 ng/mL at 28 h, and thereafter; for q4h and q2h schedules, plasma sebetralstat remained > 1000 ng/mL between first and third doses. A geometric mean PKa inhibition of > 90% was achieved within 30 min of dose 1 (all cohorts). For the q8h cohort, geometric mean PKa inhibition was > 90% for 6 h, then 84% at 8 h (before dose 2); at 16 h (before dose 3), mean inhibition was > 90%; after dose 3, mean inhibition was maintained at > 90% through 24 h, then > 80% through 28 h. Adverse events were mild and comparable between sebetralstat dosing regimens and placebo.

**Conclusions:** Three doses of sebetralstat within 24 h were well tolerated and led to drug accumulation. Geometric mean PKa inhibition of > 80% was maintained for 28 h when dosing sebetralstat q8h. We designed a 2-year, open-label, phase 3 extension trial (KONFIDENT-S, NCT05505916) which will evaluate the safety of sebetralstat while prospectively evaluating the effectiveness and safety of 600 mg sebetralstat approximately every 6 h in the periprocedural STP setting.

## O-17 One-year results from an open-label study of donidalorsen in patients with Hereditary Angioedema

### Laura Bordone^1^, Kenneth B. Newman^1^, Yiwen Deng^1^, Veronica J. Alexander^1^, Marc A. Riedl^2^, Eugene Schneider^1^, Danny M. Cohn^3^

#### ^1^Ionis Pharmaceuticals, Inc., Carlsbad, CA, USA; ^2^University of California San Diego, San Diego, La Jolla, CA, USA; ^3^Department of Vascular Medicine, Amsterdam Cardiovascular Sciences, Amsterdam University Medical Center, Amsterdam, The Netherlands

*Allergy, Asthma & Clinical Immunology* 2023, **19**(Suppl 1):O-17

**Introduction:** Hereditary Angioedema (HAE) is a potentially fatal disease characterised by unpredictable, recurrent, often disabling swelling. In a randomised phase 2 study (ISIS 721744-CS2, NCT04030598), patients with Type I and II HAE who were treated with donidalorsen reported a 90% reduction in HAE attacks and lower adverse event (AE) rates compared with placebo (71% vs 83%). We report data from the year 1 interim analysis of the open-label extension (OLE) study (ISIS 721744-CS3, NCT04307381) including quality-of-life (QoL), 8-week dosing, and pharmacodynamic data.

**Methods:** Patients who completed the randomised ISIS-721744-CS2 study through Week 17 were eligible for enrolment. The on-treatment period consisted of 2 periods: fixed (Weeks 1–13, donidalorsen 80 mg subcutaneously every 4 weeks [Q4W]) and flexible (Weeks 17–53) treatment. In the flexible period, patients continued 80 mg Q4W or switched to 80 mg every 8 weeks (Q8W) or 100 mg Q4W. Endpoints included incidence and severity of treatment-emergent adverse events (TEAEs), monthly HAE attack rate, Angioedema Quality-of-Life Questionnaire (AE-QoL) score, and effects on plasma prekallikrein (PKK) levels.

**Results:** Seventeen patients with HAE-1/HAE-2 (mean age 39 years) were enrolled. No serious AEs or patient discontinuation due to AEs were reported. Across all groups there was a 94.6% mean (100% median) reduction in HAE attacks, with a mean monthly attack rate of 0.08/month. During the flexible period, 8 patients switched to Q8W; 6 remained attack-free and stayed on this regimen; 2 experienced attacks and returned to Q4W. HAE attack rate decreased by a mean of 75.6% (median of 100%) across all 8 patients in the Q8W dosing group, with a mean monthly attack rate of 0.28. Overall AE-QoL total score improved by a mean of 24 points from baseline to Week 53 of treatment. The mean change in AE-QoL total score from baseline to Week 53 improved by 27 points and 20 points among all patients in the Q8W and Q4W dosing groups, respectively. Improvements were observed in all domains. Overall, mean plasma PKK levels decreased 56.9% from baseline to Week 53 (69.8% median). At Week 53, the mean PKK level in patients using donidalorsen Q8W was 6.0 mg/L higher than that in patients dosed Q4W.

**Conclusion:** No safety signals were identified during the 1-year OLE. Sustained reductions in HAE attack rate and improved QoL were observed. Donidalorsen Q8W also was well tolerated and effective in reducing HAE attack rates. These results confirm prior phase 2 study findings and support continued development.

## O-18 The EC_85_ derived from the oral bradykinin B2 receptor antagonist PHA121 against bradykinin effects in healthy volunteers predicts the onset and duration of its clinical effects in Hereditary Angioedema

### Marcus Maurer^1,^*, Anne Lesage^2^, Raf Crabbé^3^, Peng Lu^4^, Kees Groen^5^, Monica Rodriguez^6^, Jochen Knolle^7^, Hartmut Derendorf^8^†, Marc Riedl^9^

#### ^1^Institute of Allergology, Charité—Universitätsmedizin Berlin, Corporate Member of Freie Universität Berlin and Humboldt-Universität zu Berlin, and Fraunhofer Institute for Translational Medicine and Pharmacology ITMP, Allergology and Immunology, Berlin, Germany; ^2^GrayMatters Consulting, Schilde, Belgium; ^3^RC Consultancy, Bassins, Switzerland; ^4^Pharvaris Inc., Lexington, MA, USA; ^5^DGr Pharma, Oudenbosch, The Netherlands; ^6^Dynakin, S.L., Derio, Spain; ^7^JCK Consult, Frankfurt, Germany; ^8^University of Florida, Gainesville, FL, USA; ^9^Division of Rheumatology, Allergy and Immunology, University of California San Diego, La Jolla, CA, USA

##### *Correspondence: marcus.maurer@charite.de

*Allergy, Asthma & Clinical Immunology* 2023, **19**(Suppl 1):O-18

**Background:** PHA121 is a bradykinin B2 receptor antagonist under development for treatment (PHVS416 softgel capsule formulation) and prevention (PHVS719 extended-release tablet) of Hereditary Angioedema (HAE) attacks. A bradykinin challenge model developed in healthy volunteers was employed to determine the plasma effective threshold for bradykinin-antagonistic properties of PHA121 in HAE and to predict duration of PHA121 clinical effects.

**Materials and methods:** Proof-of-mechanism was established through PHA121-mediated inhibition of intravenous bradykinin-induced changes in blood pressure and heart rate in non-human primates (NHPs) and humans (bradykinin-challenge studies). Pharmacokinetics of PHA121 in solution and of PHVS416 and PHVS719 formulations were assessed in Phase 1 studies. Pharmacokinetics, efficacy and safety of PHVS416 for treatment of HAE attacks were evaluated in the RAPIDe-1 Phase 2 trial.

**Results:** In the NHP and human bradykinin-challenge studies, pre-treatment with PHA121 (0.1–10 mg/kg; 12 and 22 mg, respectively) inhibited bradykinin-induced hemodynamic changes with maximal efficacy reached by the first time point assessed (1 h). In the human study, the composite EC_85_ for bradykinin antagonistic effects of PHA121, as surrogate for therapeutic levels, was estimated around 13.8 ng/mL. The pharmacokinetic profile of PHA121 in healthy volunteers showed rapid absorption and plasma levels > EC_85_ within 15 min. Based on duration of PHA121 concentrations > EC_85_, the single dose of 22 mg PHA121 was predicted to maintain bradykinin-antagonistic effects for approx. 10 h, similar to 2 consecutive doses of icatibant administered 6 h apart. In the RAPIDe-1 trial, PHVS416 levels > EC_85_ were reached within 15–30 min after a single dose of PHVS416 10, 20 or 30 mg in HAE patients and remained > EC_85_ for approx. 8 (10 and 20 mg) and > 10 (30 mg) hours. In the same study, rapid onset of PHVS416 clinical effects was observed, with clinical meaningful improvements within 4 h for all doses. Rescue medication was used for a lower proportion of PHVS416- (18.9%, 10.7%, 6.5%, for 10, 20, 30 mg, respectively, at 12 h after study drug administration) vs. placebo-treated (60.8%) attacks.

**Conclusions:** In vivo bradykinin-challenge studies confirmed the bradykinin-antagonistic properties of PHA121 and allowed to determine the EC_85_ of 13.8 ng/mL as surrogate threshold for therapeutic levels. Results of Phase 1 and Phase 2 clinical studies of PHVS416 softgel capsule 10–30 mg confirmed that levels of PHA121 > EC_85_ are achieved within 15–30 min in HAE patients and are maintained for ≥ 8 h, consistent with the observed onset and duration of clinical effects for treatment of attacks.

**Trial registration**

Phase 2 RAPIDe-1 trial: ClinicalTrials.gov Identifier: NCT04618211. EudraCT Number: 2020–003445-11.

## O-19 Efficacy and safety of the oral bradykinin B2 receptor antagonist PHVS416 in treatment of Hereditary Angioedema attacks: topline results of RAPIDe-1 phase 2 trial

### Henriette Farkas^1^, John Anderson^2^, Emel Aygören-Pürsün^3^, Maria Luisa Baeza^4^, Laurence Bouillet^5^, Hugo Chapdelaine^6^, Danny M. Cohn^7^, Aurélie Du-Thanh^8^, Olivier Fain^9^, Jens Greve^10^, Mar Guilarte^11^, David Hagin^12^, Roman Hakl^13^, Joshua S. Jacobs^14^, Aharon Kessel^15^, Sorena Kiani-Alikhan^16^, Pavlína Králíčková^17^, Huamin Henry Li^18^, Ramon Lleonart^19^, Markus Magerl^20^, Michael E. Manning^21^, Avner Reshef^22^, Marc A. Riedl^23^, Bruce Ritchie^24^, Giuseppe Spadaro^25^, Maria Staevska^26^, Petra Staubach^27^, Marcin Stobiecki^28^, Gordon L. Sussman^29^, Michael D. Tarzi^30^, Anna Valerieva^26^, William H. Yang^31^, Marie-Helene Jouvin^32^, Rafael Crabbé^33^, Simone van Leeuwen^34^, Huaihou Chen^32^, Li Zhu^32^, Jochen Knolle^35^, Anne Lesage^36^, Peng Lu^32^, Marcus Maurer^20^

#### ^1^Department of Internal Medicine and Haematology, Hungarian Angioedema Center of Reference and Excellence, Semmelweis University, Budapest, Hungary; ^2^Clinical Research Center of Alabama, AllerVie Health Birmingham, AL, USA; ^3^Department for Children and Adolescents, University Hospital Frankfurt, Goethe University Frankfurt, Frankfurt, Germany; ^4^Allergy Department, Hospital General Universitario Gregorio Marañón, Madrid, Spain; ^5^National Reference Center for Angioedema (CREAK), Department of Internal Medicine, Grenoble Alpes University, Laboratoire T-RAIG, UMR 5525 TIMC-IMAG (UGA-CNRS), Grenoble, France; ^6^CHU de Montréal, Université de Montréal, Montréal, Canada; ^7^Department of Vascular Medicine, Amsterdam Cardiovascular Sciences, Amsterdam UMC, University of Amsterdam, Amsterdam, The Netherlands; ^8^Department of Dermatology, University Montpellier, Montpellier, France; ^9^Department of Internal Medicine, Sorbonne University, AP-HP, Saint Antoine Hospital, Paris, France; ^10^Department of Otorhinolaryngology, Head and Neck Surgery, Ulm University Medical Center, Ulm, Germany; ^11^Allergy Section, Internal Medicine Department, Hospital Universitari Vall d'Hebron, Barcelona, Spain; ^12^Allergy and Clinical Immunology Unit, Department of Medicine, Tel Aviv Sourasky Medical Center and Sackler Faculty of Medicine, University of Tel Aviv, Tel Aviv, Israel; ^13^Department of Clinical Immunology and Allergology, St. Anne's University Hospital in Brno and Faculty of Medicine, Masaryk University, Brno, Czech Republic; ^14^Allergy and Asthma Clinical Research, Walnut Creek, CA, USA; ^15^Bnai Zion Medical Center, Technion-Israel Institute of Technology, Haifa, Israel; ^16^Department of Immunology, Royal Free London NHS Foundation Trust, London, UK; ^17^Institute of Clinical Immunology and Allergy, University Hospital Hradec Kralove, Charles University, Faculty of Medicine in Hradec Kralove, Hradec Kralove, Czech Republic; ^18^Institute for Asthma and Allergy, Chevy Chase, MD, USA; ^19^Allergology Service, Bellvitge University Hospital, L'Hospitalet de Llobregat, Barcelona, Spain; ^20^Institute of Allergology, Charité - Universitätsmedizin Berlin, Corporate Member of Freie Universität Berlin and Humboldt-Universität zu Berlin, and Fraunhofer Institute for Translational Medicine and Pharmacology ITMP, Allergology and Immunology, Berlin, Germany; ^21^Allergy, Asthma and Immunology Associates, Ltd., Scottsdale, AZ, USA; ^22^Allergy, Immunology and Angioedema Center, Barzilai University Hospital, Ashkelon, Israel; ^23^Division of Rheumatology, Allergy and Immunology, University of California San Diego, La Jolla, CA, USA; ^24^Division of Hematology, Department of Medicine, University of Alberta, Edmonton, AB, Canada; ^25^Department of Translational Medical Sciences and Center for Basic and Clinical Immunology Research (CISI), University of Naples Federico II, Napoli, Italy; ^26^Department of Allergology, Clinic of Allergology, University Hospital "Alexandrovska", Medical University of Sofia, Sofia, Bulgaria; ^27^Department of Dermatology, University Medicine Mainz, Mainz, Germany; ^28^Department of Clinical and Environmental Allergology, Jagiellonian University Medical College, Krakow, Poland; ^29^Gordon Sussman Clinical Research Inc, Toronto, ON, Canada; ^30^Department of Medicine, Brighton and Sussex Medical School, Brighton, UK; ^31^Ottawa Allergy Research Corporation, Department of Medicine, University of Ottawa, Ottawa, ON, Canada; ^32^Pharvaris Inc., Lexington, MA, USA; ^33^RC Consultancy, Bassins, Switzerland; ^34^SLC Consultancy, Woerden, The Netherlands; ^35^JCK Consult, Frankfurt, Germany; ^36^GrayMatters Consulting, Schilde, Belgium

##### *Correspondence: farkas.henriette@med.semmelweis-univ.hu

*Allergy, Asthma & Clinical Immunology* 2023, **19**(Suppl 1):O-19

**Background:** Swelling attacks of Hereditary Angioedema (HAE) are caused by an excess of bradykinin that activates bradykinin B2 receptors. PHA121 is a potent and selective bradykinin B2 receptor antagonist under development for on-demand and prophylactic treatment of HAE.

**Materials and Methods:** RAPIDe-1 was a phase 2, double-blind, placebo-controlled, cross-over, dose-ranging trial of PHVS416, oral softgel capsule formulation of PHA121, for treatment of attacks in HAE due to C1-INH deficiency. Seventy-four participants aged ≥ 18 and ≤ 75 years, diagnosed with HAE-1/-2, with ≥ 3 attacks in the last 4 months or ≥ 2 attacks in the last 2 months prior to screening, were enrolled from 31 Sites in Canada, Europe, Israel, the United Kingdom, and the United States.

**Results:** The primary analysis included 147 qualifying HAE attacks treated by 62 participants with double-blinded study drug (placebo or PHVS416 10, 20, or 30 mg). Analysis of the primary endpoint showed that PHVS416 significantly reduced attack symptoms measured as change in the mean 3-symptom composite (skin pain, skin swelling, abdominal pain) visual analogue scale (VAS-3) score during HAE attacks: at 4 h, the least squares mean difference of VAS-3 change for PHVS416 10, 20 and 30 mg were − 16.75, − 15.02, and − 16.28 compared to placebo, respectively (Table 1). All secondary endpoints were met with PHVS416 significantly reducing time to onset of symptom relief [≥ 30% reduction in VAS-3; median (hours): 2.1–2.7 for PHVS416 at 3 doses vs. 8.0 for placebo], time to ≥ 50% reduction in VAS-3 [median (hours): 3.3–4.0 for PHVS416 at 3 doses vs. 22.8 for placebo], and time to almost complete or complete resolution of symptoms [all 3 VAS item scores ≤ 10; median (hours): 5.8–20.0 for PHVS416 at 3 doses vs. 42.0 for placebo] (Table 1). Treatment with PHVS416 improved Mean Symptom Severity Score (MSCS) and Treatment Outcome Score (TOS) vs. placebo (Table 1). PHVS416 also substantially reduced the use of rescue medication vs. placebo: 18.9%, 10.7%, and 6.5% in attacks treated with PHVS416 10, 20 and 30 mg, respectively, vs. 60.8% in attacks treated with placebo, within 12 h. PHVS416 was generally well tolerated with 3 treatment-related adverse events (TRAEs) reported for 1 PHVS416 30 mg-treated attack (2.8%) and 1 TRAE reported for 1 placebo-treated attack (1.9%).

**Conclusions:** The consistent results of the primary analysis of the RAPIDe-1 trial across all endpoints provide evidence in support of efficacy and safety of PHVS416 in treating HAE attacks and support its further development as a potential on-demand therapy for HAE.

**Trial registration**

ClinicalTrials.gov Identifier: NCT04618211. EudraCT Number: 2020-003445-11.

**Table 1 (abstract O-19):** Result summary of RAPIDe-1 primary and key secondary efficacy endpoints

	Placebo N = 51	PHVS416 10 mg N = 37	PHVS416 20 mg N = 28	PHVS416 30 mg N = 31	Combined PHVS416* N = 96
Mean VAS-3 at pre-treatment	27.76	26.16	25.46	29.73	27.11
Change in VAS-3 at 4 h					
Least-squares mean difference:					
PHVS416—placebo		− 16.75	− 15.02	− 16.28	− 16.08
p-value		< 0.0001	< 0.0001	< 0.0001	
Time to onset of symptom relief by VAS-3 ≥ 30% reduction^a^					
Median time in hours (95% CI)	8.0 (7.6, 46.9)	2.1 (1.5, 2.9)	2.7 (1.9, 3.5)	2.5 (1.9, 3.8)	2.4 (2.0, 2.9)
Hazard ratio		3.81	3.08	3.61	
p-value		< 0.0001	0.0021	< 0.0001	
Time to VAS-3 ≥ 50% reduction^a^					
Median time in hours (95% CI)	22.8 (20.0, 24.1)	3.3 (2.4, 3.9)	4.0 (2.9, 6.0)	4.0 (3.3, 5.8)	3.9 (3.0, 4.8)
Hazard ratio		4.55	3.65	3.87	
p-value		< 0.0001	0.0003	< 0.0001	
Time to almost complete or complete symptom relief by VAS-3^a^					
Median time in hours (95% CI)	42.0 (22.0, 48.1)	5.8 (3.6, 7.5)	20.0 (4.5, 20.0)	20.0 (6.0, 20.1)	7.5 (5.9, 20.0)
Hazard ratio		5.09	2.25	2.65	
p-value		< 0.0001	0.0127	0.0001	
Change in MSCS score at 4 hours^b^					
Least-squares mean difference:					
PHVS416—placebo		− 0.79	− 0.61	− 0.39	− 0.61
p-value		< 0.0001	0.0008	0.0291	
TOS at 4 hours^b^					
Least-squares mean difference:					
PHVS416—placebo		64.13	62.69	71.06	66.05
p-value		< 0.0001	< 0.0001	< 0.0001	

N = The number of attacks included in the mITT Analysis Set. p-values for PHVS416 20mg and PHVS416 30mg are based on statistical tests in the pre-specified multiple comparison procedure, other p-values are nominal.

^a^Hazard ratios and p-values are based on marginal Cox proportional hazards models.

^b^p-values are based on mixed-effects models for repeated measures.

*The combined PHVS416 results are based on post-hoc analyses to provide a reference of the result by pooling all three active doses.

## O-20 Design of ALPHA-STAR, a phase 1b/2 proof-of-concept trial of STAR-0215 as a long-active preventative therapy in patients with Hereditary Angioedema (HAE) types I or II

### Christopher Morabito^1^, Chris Stevens^1^, Kristine Bernard^1^, Marianne Magill^1^, Joan Kelly^1^, Jou-Ku Chung^1^, Michele Gunsior^1^, Claire VanEenwyck^2^, Marcus Mauer^3^

#### ^1^Astria Therapeutics, Boston, USA; ^2^Parexel International, Durham, USA; ^3^Institute of Allergology, Charité - Universitätsmedizin Berlin, Corporate Member of Freie Universität Berlin and Humboldt-Universität zu Berlin, and Fraunhofer Institute for Translational Medicine and Pharmacology ITMP, Allergology and Immunology, Berlin, Germany

*Allergy, Asthma & Clinical Immunology* 2023, **19**(Suppl 1):O-20

**Rationale:** HAE caused by C1-INH deficiency results in uncontrolled activation of plasma kallikrein that initiates potentially life-threatening HAE attacks. STAR-0215 is an investigational humanized YTE-modified IgG1kappa monoclonal antibody with an estimated half-life of 117 days and potent and durable (at least 84 days) reduction of plasma kallikrein activity demonstrated in healthy adult subjects. ALPHA-STAR is the first in-patient trial of STAR-0215.

**Methods:** ALPHA-STAR (Astria Long-Acting Prophylaxis for Hereditary Angioedema-STAR-0215) is a global, multi-center Phase 1b/2 open-label POC trial in people with C1-INH HAE (types I or II). HAE patients with ≥ 4 HAE attacks in the prior 12 months and not receiving preventative therapy at the time of screening may enter an 8-week run-in period that will establish baseline HAE attack rate. Participants who have ≥ 2 attacks in the run-in may receive one dose (450 mg subcutaneously (SC), n = 4) or two doses (600 mg SC followed by 300 mg 3 months later, n ≤ 14) of STAR-0215. Participants will be followed for changes in safety, HAE attack rates, pharmacokinetics, pharmacodynamics (PD), and Angioedema-Quality of Life for 6 months (168 days) after the last dose. PD will be evaluated by measuring changes in cleaved high molecular weight kininogen.

**Results:** Results from this POC trial will determine whether STAR-0215 may reduce HAE attacks for at least 3 months (84 days) with a favorable safety profile. Single doses of 450 mg and 600 mg are expected to produce mean concentrations at least 1.5 × above the threshold predicted for HAE attack suppression through 84 days and result in robust plasma kallikrein inhibition. In addition, this trial will inform dose selection for Phase 3, including the loading dose, and will assess the effects of 300 mg as a potential maintenance dose. Durability of effects will also be assessed out to 6 months after the last dose.

**Conclusions:** Results from this trial will be used to determine the potential safety, tolerability, and efficacy of STAR-0215 as a long-acting preventative therapy for HAE and to plan for future clinical development.

## O-21 Transient exposure to NTLA-2002, an investigational CRISPR/Cas9-based gene editing therapy, leads to durable pharmacodynamic responses and attack control in patients with Hereditary Angioedema

### Hilary Longhurst^1^, Padmalal Gurugama^2^, Carri Boiselle^3^, Stacey Shea^3^, Christina Picornell^3^, Ahmed Abdelhady^3^, Adele Golden^3^, Mrinal Y. Shah^3,*^, David Maag^3^, Danny M. Cohn^4^

#### ^1^Te Whatu Ora/Te Toka Tumai, Auckland, New Zealand; ^2^Cambridge University Hospitals, NHS Foundation Trust, Cambridge, UK; ^3^Intellia Therapeutics, Cambridge, MA, USA; ^4^Amsterdam University Medical Centers, Amsterdam, The Netherlands

##### *Correspondence: mrinal.shah@intelliatx.com

*Allergy, Asthma & Clinical Immunology* 2023, **19**(Suppl 1):O-21

**Introduction:** Hereditary Angioedema (HAE) is a rare, autosomal dominant genetic disease associated with frequent, severe, and unpredictable attacks of swelling due to dysregulated bradykinin production. Treatments targeting kallikrein, a protease encoded by the *KLKB1* gene, significantly reduce attack frequency and improve patient quality of life. NTLA-2002 is an investigational CRISPR/Cas9-based in vivo gene editing therapy targeting *KLKB1* in the liver, with the goal of achieving lifelong control of HAE attacks after a single dose.

**Materials and Methods:** A first-in-human, phase 1/2 study of NTLA-2002 in adults with HAE is ongoing, with enrollment complete in the phase 1 dose-escalation portion. The primary endpoint is safety/tolerability; secondary and exploratory endpoints include pharmacokinetics, pharmacodynamics, and clinical efficacy. Plasma kallikrein protein was determined by immunoassay; kallikrein activity was determined by plasma kallikrein enzymatic assay. NTLA-2002 pharmacokinetics were characterized by quantifying 2 lipid and 2 RNA components of the drug product by liquid chromatography-mass spectrometry and quantitative reverse transcription PCR, respectively.

**Results:** Three dose cohorts (25 mg, n = 3; 50 mg, n = 4; 75 mg, n = 3) were enrolled. At the time of this interim analysis, median duration of follow-up was 3.8 months (range, 1.0–9.6). Across all dose levels, the most frequent adverse events (AEs) were infusion-related reactions and fatigue. All treatment-emergent AEs were Grade 1 or 2 only. No clinically significant laboratory findings, or treatment-emergent serious AEs, were observed. All patients in the 25 mg and 75 mg cohorts completed the 16-week primary observation period and had an ongoing attack-free interval of 2.3–10.6 months, with a mean reduction in monthly attack rate of 89% from weeks 5–16 vs baseline. Steady-state plasma kallikrein protein reduction was observed in a dose-dependent manner, with mean reductions from baseline of 65%, 81%, and 92% at the 25 mg, 50 mg, and 75 mg doses, respectively. A similar trend in reduction was observed for kallikrein activity. Interim pharmacokinetic data from the 25 mg and 75 mg cohorts demonstrated that ionizable lipid exhibited a rapid decline from peak levels, followed by a secondary peak, and then a log-linear elimination phase. All lipid components declined below the limit of quantitation after day 15 post-infusion in all patients.

**Conclusions:** A single dose of NTLA-2002 was well-tolerated, and led to rapid, robust, dose-dependent, and durable reductions in total plasma kallikrein protein and activity after transient exposure, with clinically significant reduction in attack rate observed in the cohorts analyzed thus far (25 mg and 75 mg). Additional measures of pharmacodynamic responses and dose–response analyses will be presented.

## O-22 Prototype of a mobile application to record prodromes and attacks of Hereditary Angioedema

### Iris Leibovich-Nassi^1,2,*^, Avner Reshef^1^, Erez Shalom^3^, Irina Komarova^1^, Yuval Shahar^3^

#### ^1^Angioedema Research Center, Barzilai University Medical Center, Ashkelon, Israel; ^2^Barzilai Academic Nursing School, Ashkelon, Israel; ^3^Department of Information Systems Engineering, Ben Gurion University, Beer Sheva, Israel

##### *Correspondence: irisl@bmc.gov.il

*Allergy, Asthma & Clinical Immunology* 2023, **19**(Suppl 1):O-22

**Background:** Personal mobile applications (Apps) are already integrated into clinical practice and have become a useful clinical and educational instrument for health management [1]. Recognizing early signs and symptoms preceding Hereditary Angioedema (HAE) attacks (prodromes) may help patients to anticipate impending attacks and prepare for self-treatment that may shorten the attacks [2]. A new instrument (HAE-EPA), based on Patient-reported Outcome Measures (PROMs), has been recently developed to assess prodromes and attacks [3]. The tool has shown its robustness and content validity in evaluating prodromes and their usefulness in predicting attacks. It can alert the patients and help them to deploy therapeutic strategies to preempt the attacks. In the modern digital environment, a personal App on a personal cellular phone can become a handy tool, helping patients evaluate a developing attack in real-time. The App should be able to record early prodromal signs and symptoms, monitor an evolving attack, help make clinical decisions, and transmit data to physicians and disease registries.

**Materials and methods:** We designed, developed, and tested a prototype of a mobile application (iHAE-EPA) capable of storing individual patient reports, analysis of data, and monitoring clinical parameters that assist in managing HAE. The App was developed in a "Flutter" cross-platform technology that operates on Android and Apple iOS operating systems. It resides on a network server, is accessible through an HTTPS protocol, and can code data "parcels" between the clients and the server. Personal and health information are securely protected. Each patient was allotted an ID during the registration, and the data was transmitted to a secure MongoDB database. When signs or symptoms of HAE approached, the users could navigate through the app pages, quickly fill out the questionnaires, connect to their physician, and send the coded data for storage on the server.

**Results:** A group of 10 experienced HAE patients tested the App by sending real-time data to the server and their physician. Patients filled out a utility test rating: connectivity (100%), accessibility (97%), ease-of-use (97%), content visibility (97%), messaging clarity (93%), and general satisfaction from the App (95%). Two HAE expert physicians confirmed that the App helped manage and communicate with patients. Most patients affirmed that the App could help them better manage their disease and enhance communication with physicians.

**Conclusions:** We developed a cellular-based disease-specific PROM instrument and tested a prototype, demonstrating its high practicality in managing HAE prodromes and attacks.

**Acknowledgment**

Students of the Dpt. of Information Systems Engineering, Ben Gurion University, Beer Sheva.

**References**

1. Timmers T, Janssen L, Kool RB, Kremer JA. Educating Patients by Providing Timely Information Using Smartphone and Tablet Apps: Systematic Review. J Med Internet Res. 2020;22(4): e17342.
2. [1]Timmers T, Janssen L, Kool RB, Kremer JA. Educating Patients by Providing Timely Information Using Smartphone and Tablet Apps: Systematic Review. J Med Internet Res. 2020;22(4): e17342.
3. [3]Leibovich-Nassi I, Golander H, Somech R, Har-Even D, Reshef A. New Instrument for the Evaluation of Prodromes and Attacks of Hereditary Angioedema (HAE-EPA). Clin Rev Allergy Immunol. 2021;61(1):29-39

## O-23 Garadacimab for Hereditary Angioedema prophylaxis: long term efficacy and safety from the Phase 3 VANGUARD trial and first interim analysis of the open-label extension trial

### Avner Reshef^1,^*, Markus Magerl^2^, Inmaculada Martinez Saguer^3^, Jonathan A. Bernstein^4^, Henriette Farkas^5^, William H. Yang^6^, Joshua S. Jacobs^7^, Philip Hei Li^8^, William R. Lumry^9^, Emel Aygören-Pürsün^10^, Isao Ohsawa^11^, Constance Katelaris^12^, Hilary Longhurst^13^, Roman Hakl^14^, Mar Guilarte^15^, Erik S.G. Stroes^16^, Henrike Feuersenger^17^, Mihai Alexandru Bica^17^, Iris Jacobs^18^, Timothy J. Craig^19^

#### ^1^Allergy, Immunology & Angioedema Center, Barzilai University Hospital, Ashkelon, Israel; ^2^Institute of Allergology, Charité–Universitätsmedizin Berlin, Corporate Member of Freie Universität Berlin and Humboldt-Universität zu Berlin, Berlin, and Frauhofer Institute for Translational Medicine and Pharmacology ITMP, Immunology and Allergology, Berlin, Germany; ^3^HZRM Haemophilia Center Rhein Main, Mörfelden-Walldorf, Germany; ^4^Department of Internal Medicine Division of Rheumatology, Allergy and Immunology and the Bernstein Clinical Research Center Cincinnati, University of Cincinnati, Cincinnati, OH, USA; ^5^Department of Internal Medicine and Haematology, Hungarian Angioedema Center of Reference and Excellence, Semmelweis University, Budapest, Hungary; ^6^Ottawa Allergy Research Corporation, Department of Medicine, University of Ottawa, Ottawa, ON, Canada; ^7^Allergy & Asthma Clinical Research, Walnut Creek, CA, USA; ^8^Department of Medicine, School of Clinical Medicine, University of Hong Kong, Hong Kong; ^9^AARA Research Center, Dallas, TX, USA; ^10^Klinikum der Johann Wolfgang-Goethe Universität, Klinik für Kinder- und Jugendmedizin, Frankfurt, Germany; ^11^Department of Nephrology, Saiyu Soka Hospital, Saitama, Japan; ^12^Allergy and Immunology Services, Campbelltown Hospital, Sydney, Australia; ^13^Department of Medicine, University of Auckland, New Zealand and Department of Immunology, Auckland City Hospital, Auckland, New Zealand; ^14^Department of Clinical Immunology and Allergology, St. Anne´s University Hospital Brno, and Faculty of Medicine, Masaryk University, Brno, Czech Republic; ^15^Unitat d'Al·lèrgia, Hospital Universitari Vall d'Hebron, Barcelona, Spain; ^16^Division of Vascular Medicine, Amsterdam UMC, Amsterdam, The Netherlands; ^17^CSL Behring Innovation GmbH, Marburg, Germany; ^18^CSL Behring, King of Prussia, PA, USA; ^19^Allergy, Asthma and Immunology, Department of Medicine and Pediatrics, Penn State University, Hershey, PA, USA

##### *Correspondence: aresh@netvision.net.il

*Allergy, Asthma & Clinical Immunology* 2023, **19**(Suppl 1):O-23

**Background:** Once-monthly subcutaneous (SC) garadacimab prophylaxis demonstrated an 87% mean reduction in monthly Hereditary Angioedema (HAE) attacks vs placebo, with a favourable safety profile in the pivotal Phase 3 VANGUARD study [1]. Long-term evaluation is ongoing in an open-label extension (OLE) (NCT04739059).

**Methods:** The OLE is evaluating once-monthly SC garadacimab 200 mg for ≥ 12 months. The study comprises patients rolled-over from the Phase 2 [2] and pivotal Phase 3 studies [1], and additional newly-enrolled garadacimab-naïve patients. Primary endpoint is treatment-emergent adverse events (TEAEs). Secondary endpoints include time-normalised HAE attack rate and reduction vs run-in.

**Results:** As of 30 September 2022, 161 patients received garadacimab (n = 92 roll-over; n = 69 garadacimab-naïve), 41/161 (25.5%) had ≥ 12 months of exposure. Of safety analysis set (n = 159), 119 patients (74.8%) experienced ≥ 1 TEAE. Most TEAEs (367/375, 97.9%) were mild or moderate. No deaths or TEAEs of special interest (thromboembolic events, abnormal bleeding, severe hypersensitivity including anaphylaxis) reported. Three patients (1.9%) reported serious non-garadacimab-related TEAEs (COVID-19 [1 severe, 1 moderate], 1 abdominal HAE attack). Overall, 19 patients (11.9%) experienced 41 garadacimab-related TEAEs, of which 30 (73.2%) were injection-site reactions (28/30 mild; 2/30 moderate). One patient discontinued due to moderate injection-site reaction.

Mean time-normalised monthly attack rate (95% confidence interval [CI]) was 0.17 (0.11–0.23) vs 3.57 (3.20–3.95) during run-in, corresponding to a 94.2% reduction (95% CI 92.2–96.2), consistent with observed mean reduction vs. run in for garadacimab-treated patients (n = 39) of 90.7% in the pivotal Phase 3 study. For garadacimab-treated patients in the Phase 3 study who rolled-over to OLE (n = 36; median [range] OLE exposure, 7.5 months [4.9–11.9], plus additional 6 months from pivotal Phase 3 study), reduction in mean attack rate vs run-in was 95.8%. Median (interquartile range) monthly attack rate in the OLE was 0.0 (0.17) vs 2.85 (2.45) during run-in, corresponding to a 100% reduction.

In the OLE, 101 patients (62.7%) were attack-free for a mean exposure per patient of 9.6 months (range 3.0–16.7), generally consistent with the results of the pivotal Phase 3 study (24/39; 61.5%). Over the first 3 months of the pivotal Phase 3 study, 28/39 patients (71.8%) were attack-free (vs 2/24 for placebo [8.3%]), and similarly over the first 3 months of the OLE, 122 (75.8%) were attack-free.

**Conclusions:** Generally consistent with the pivotal Phase 3 study results, the OLE demonstrates that garadacimab has a favourable safety profile and sustained efficacy as routine prophylaxis to prevent HAE attacks.

**References**

1. Craig T, Reshef A, Li H, et al. Efficacy and safety of garadacimab, a factor XIIa inhibitor for Hereditary Angioedema prevention (VANGUARD): a global, multicentre, randomised, double-blind, placebo-controlled, phase 3 trial. Lancet. 2023. Published online February 28, 2023, DOI: https://doi.org/10.1016/S0140-6736(23)00350-1
2. Craig T, Magerl M, Levy DS, et al. Prophylactic use of an anti-activated factor XII monoclonal antibody, garadacimab, for patients with C1-esterase inhibitor-deficient Hereditary Angioedema: a randomised, double-blind, placebo-controlled, phase 2 trial. Lancet. 2022; 399:945–955

## O-24 Effect of prophylactic immunomodulation in non-human primates treated with BMN 331, an AAV5 gene therapy for Hereditary Angioedema

### M. Benjamin Hock^1,^*, Kristin Obrochta Moss^1^, Madhav Sachar^1^, Kristin Tracy^1^, Todd Oppeneer^1^, Gao Dong^1^, Aryun Kim^1^, Jill Woloszynek^1^, Jack Brownrigg^2^, Thomas Machnig.^2^

#### ^1^BioMarin Pharmaceutical Inc., Novato, CA, USA; ^2^BioMarin (UK) Ltd., London, UK

##### *Correspondence: ben.hock@bmrn.com

*Allergy, Asthma & Clinical Immunology* 2023, **19**(Suppl 1):O-24

**Introduction:** Hereditary Angioedema due to C1-INH inhibitor deficiency (HAE-C1-INH) is due to mutations in *the SERPING1* gene. HAErmony-1 (331-201) is the first in-human gene transfer study in Type I/ II HAE patients investigating an AAV5 mediated gene therapy that contains the human *SERPING1* gene (BMN 331) for the prevention of HAE attacks. Several immunomodulation approaches, including corticosteroids, have been utilized in previous AAV gene therapy clinical trials to mitigate the risk of transaminitis. Study 331-201 employs a prophylactic corticosteroid course over 14 weeks. The effect of prophylactic corticosteroid on the *SERPING1* transgene expression is not known. This non-human primate study characterized the expression of human (hC1-INH) derived from BMN 331 in the presence or absence of prophylactic corticosteroid treatment.

**Methods:** A study in female cynomolgus monkeys (n = 5/group) evaluated BMN 331 at 6E14 vg/kg in animals with or without prophylactic methylprednisolone sodium succinate (MPS) treatment [prior to injection of BMN 331 and then daily for approximately four weeks (IM injection of 10 mg/kg/day for majority of the period) post-BMN 331 dose]. Cynomolgus and human C1-INH protein levels in plasma were determined using a protein digestion and solid phase extraction (SPE) procedure and analysis by UHPLC-MS/MS. Terminal samples were harvested 12 weeks after BMN 331 dose.

**Results:** Total plasma hC1-INH protein concentrations after administration of 6E14 vg/kg BMN 331 were greater with prophylactic corticosteroids than without by approximately fivefold with no difference in time to peak concentration. Analyses of target liver tissues demonstrated that steroid administration at the time of BMN 331 dosing resulted in increased total vector DNA, accessible vector DNA (as determined by ATAC seq), and expression of transgene RNA. Endogenous cynomolgus C1-INH expression was variable but generally consistent over time, and similar with and without corticosteroid treatment. There was no overt impact on the safety endpoints related to the use of corticosteroids. BMN 331-related changes (with or without corticosteroid treatment) in clinical chemistry parameters were limited to a minimal increase in alanine aminotransferase (ALT) and aspartate aminotransferase (AST) on Day 3, with values that generally trended toward baseline (Day -1) by Day 15.

**Conclusion:** This non-human primate study demonstrated that levels of expression of hC1-INH protein derived from BMN 331 were enhanced in animals receiving prophylactic corticosteroids initiated prior to gene therapy. These nonclinical findings support the clinical study design and employed corticosteroid regimen in HAErmony-1 investigating the safety and efficacy of *SERPING1* gene transfer in HAE patients.

**Ethics Approval:** This protocol and any amendment(s) or procedures involving the care and use of animals in this study were reviewed and approved by Charles River—Nevada Institutional Animal Care and Use Committee (IACUC) before conduct. During the study, the care and use of animals was conducted with guidance from the guidelines of the USA National Research Council.

## O-25 Garadacimab for Hereditary Angioedema prophylaxis: efficacy and safety from a Phase 2 open-label extension trial

### Timothy J. Craig^1^, Avner Reshef^2^, William R. Lumry^3^, Inmaculada Martinez Saguer^4^, Joshua S. Jacobs^5^, William H. Yang^6^, Emel Aygören-Pürsün^7^, Paul K. Keith^8^, Paula Busse^9^, Markus Magerl^10^, Henrike Feuersenger^11^, Mihai Alexandru Bica^11^, Fiona Glassman^12^, Ingo Pragst^11^, Donald S. Levy^13^

#### ^1^Allergy, Asthma and Immunology, Department of Medicine and Pediatrics, Penn State University, Hershey, PA, USA; ^2^Allergy, Immunology & Angioedema Center, Barzilai University Hospital, Ashkelon, Israel; ^3^AARA Research Center, Dallas, TX, USA; ^4^HZRM Haemophilia Center Rhein Main, Mörfelden-Walldorf, Germany; ^5^Allergy & Asthma Clinical Research, Walnut Creek, CA, USA; ^6^Ottawa Allergy Research Corporation, Department of Medicine, University of Ottawa, Ottawa, ON, Canada; ^7^Klinikum der Johann Wolfgang-Goethe Universität, Klinik für Kinder- und Jugendmedizin, Frankfurt, Germany; ^8^McMaster University Medical Centre Site, Hamilton, ON, Canada; ^9^Mount Sinai, New York, NY, USA; ^10^Institute of Allergology, Charité – Universitätsmedizin Berlin, and Fraunhofer Institute for Translational Medicine and Pharmacology ITMP, Immunology and Allergology, Berlin, Germany; ^11^CSL Behring Innovation GmbH, Marburg, Germany; ^12^CSL Behring, King of Prussia, PA, USA; ^13^Division of Allergy and Immunology, University of California, Irvine, CA, USA

*Allergy, Asthma & Clinical Immunology* 2023, **19**(Suppl 1):O-25

**Background:** Once-monthly 200 mg subcutaneous (SC) garadacimab (a fully human anti-activated factor XII monoclonal antibody) has demonstrated reduction in Hereditary Angioedema (HAE) attack rate vs placebo in Phase 2 (TP1; NCT03712228) [1] and pivotal Phase 3 studies [2], respectively. Long-term (over 2 years) safety and efficacy data from the final analysis of the open-label extension (OLE) part of the Phase 2 trial are reported here.

**Methods:** In TP1, after 4–8 week run-in, patients were randomised to placebo or garadacimab once-monthly (28 ± 2 days; 75, 200 or 600 mg) or assigned to garadacimab every 2 weeks (400 mg). In OLE, patients received 200 mg or 600 mg garadacimab (either continued from TP1 or re-randomised); after protocol amendment, patients receiving 600 mg garadacimab underwent dose reduction to 200 mg. Primary endpoint was time-normalized number of HAE attacks per month. Secondary endpoints included safety.

**Results:** Of 38 patients who received garadacimab in the OLE, 21 (55.3%) were female and median age was 39.0 years [interquartile range (IQR) 27.0–53.0]. Median (IQR) exposure was 727.0 days (80–784.0). Median (IQR) number of time-normalized HAE attacks per month was 0.0 (0.0–0.1) for garadacimab 200 mg (n = 36) and 0.1 (0.0–0.2) for garadacimab 600 mg (n = 18), corresponding to median (IQR) reduction vs run-in of 100.0% (98.4–100.0%) for 200 mg garadacimab and 98.0% (95.4–100.0%) for 600 mg garadacimab. Mean [standard deviation (SD)] time-normalized number of HAE attacks per month was 0.1 (0.2) for garadacimab 200 mg (n = 36) and 0.2 (0.3) for garadacimab 600 mg (n = 18), corresponding to mean (SD) reduction vs run-in of 97.8% (5.4) for 200 mg garadacimab and 93.2% (12.5) for 600 mg garadacimab. A total of 36/38 patients (94.7%) experienced ≥ 1 treatment-emergent adverse event (TEAE); most were mild or moderate. Most frequently reported TEAEs were headache (9/38; 23.7%), abdominal pain (7/38; 18.4%), injection-site reactions, nasopharyngitis, upper respiratory tract infections, and pain in extremity (5/38 for each; 13.2%). All reported injection-site reactions were mild. Laboratory abnormalities reported as AEs were considered unrelated to garadacimab. Two serious TEAEs not related to garadacimab were reported (diverticular perforation, asthma). No TEAE of special interest (thromboembolic, abnormal bleeding, severe hypersensitivity including anaphylaxis) or TEAEs leading to discontinuation or death were reported. No anti-drug antibodies that could potentially impact the efficacy of garadacimab were observed.

**Conclusions:** Prophylaxis with once-monthly SC garadacimab for over 2 years was effective in preventing HAE attacks with a favourable safety profile.

**References**

1. [1]Craig T, Magerl M, Levy DS, et al. Prophylactic use of an anti-activated factor XII monoclonal antibody, garadacimab, for patients with C1-esterase inhibitor-deficient Hereditary Angioedema: a randomised, double-blind, placebo-controlled, phase 2 trial. Lancet. 2022; 399:945–955.
2. [2]Craig T, Reshef A, Li H, et al. Efficacy and safety of garadacimab, a factor XIIa inhibitor for Hereditary Angioedema prevention (VANGUARD): a global, multicentre, randomised, double-blind, placebo-controlled, phase 3 trial. Lancet. 2023. Published online February 28, 2023, DOI: https://doi.org/10.1016/S0140-6736(23)00350-1

## O-26 Prophylaxis of angioedema attacks due to acquired C1-inhibitor deficiency with PHA121, a novel oral bradykinin B2 receptor antagonist

### Remy S. Petersen, Lauré M. Fijen, Johannes P. Kelder, Danny M. Cohn

#### Department of Vascular Medicine, Amsterdam Cardiovascular Sciences, Amsterdam UMC, University of Amsterdam, Amsterdam, The Netherlands

##### *Correspondence: r.s.petersen@amsterdamumc.nl

*Allergy, Asthma & Clinical Immunology* 2023, **19**(Suppl 1):O-26

**Background:** Acquired C1-inhibitor deficiency (AAE-C1-INH) is a rare condition characterized by recurrent episodes of angioedema. There is an unmet need for effective prophylactic treatment as there are no therapies licensed for this condition. The bradykinin B2 receptor antagonist icatibant is effective in treating angioedema attacks in this patient population, however, due to its relatively short half-life it is unsuitable for prophylaxis. PHA121 is a selective, orally bioavailable, competitive bradykinin B2 receptor antagonist. In this proof-of-concept study, we aimed to investigate the efficacy and safety of prophylactic treatment with PHA121 as softgel capsule formulation (PHVS416) for AAE-C1-INH.

**Methods:** A double-blind, placebo-controlled, randomized, cross-over intervention study was conducted in AAE-C1-INH patients that continued to have angioedema attacks after treatment with rituximab. All participants provided written informed consent. Participants were randomly allocated to one of two treatment arms: a 20 mg dose of PHVS416 or placebo b.i.d. for a total duration of eight weeks, followed by a cross-over to the other treatment arm. The primary outcome was the number of investigator-confirmed angioedema attacks during the treatment period. Safety was evaluated through the occurrence of treatment-related adverse events including clinically significant changes in vital signs, laboratory tests and ECG.

**Results:** A total of three patients were enrolled in the study. All patients were male and respectively 38, 56 and 68 years of age. The patients had an attack frequency of 5.0, 2.0 and 1.1 attacks per month respectively in the year previous to participation. Two patients had previously been diagnosed with a monoclonal gammopathy and one patient did not have an identified associated disorder. Anti-C1-inhibitor antibodies were present in two of the three patients. The last visit of the last subject is expected at 16 March 2023.

The primary outcome and safety results will be shared in the presentation.

**Conclusion:** In this proof-of-concept, randomized, double-blind, placebo-controlled, cross-over study, a total of three patients were enrolled and treated with PHA121 and placebo b.i.d. in a random order.

All patients gave explicit permission for their information to be published.

## O-27 Patient case series of Hereditary Angioedema with normal C1-inhibitor and factor XII mutation: findings from an allergy and immunology department in Argentina

### Ricardo D. Zwiener^1,^*, Natalia Fili^2^, Menendez Alejandra^3^, Rozenfeld Paula^4^

#### ^1^Department of Allergy and Immunology, Hospital Universitario Austral, Pilar, Buenos Aires, Argentina; ^2^Department of Allergy and Immunology, Hospital Materno Infantil, Salta, Argentina; ^3^Asociación Argentina de Angioedema Hereditario, Argentina; ^4^ IFP, University of La Plata-CONICET, La Plata, Buenos Aires, Argentina

##### *Correspondence: ricardozwiener@hotmail.com

*Allergy, Asthma & Clinical Immunology* 2023, **19**(Suppl 1):O-27

Hereditary Angioedema (HAE) is a rare genetic disease associated with either a quantitative or qualitative deficiency in C1-inhibitor (HAE C1-INH) or normal C1-INH (HAE nC1-INH) [1]. HAE nC1-INH can be caused by mutations in different genes: Factor 12 gene (F12), plasminogen gene (PLG), angiopoietin gene (ANGPT1), kininogen 1 (KNG1), myoferlin (MYOF) and heparan sulfate (HS)-glucosamine 3-O-sulfotransferase 6 gene (HS3ST6) [4].

Mutations in F12 gene have been associated with familial angioedema and account for up to 25% patients with nC1-INH-HAE [2]. Point mutation (Thr328Lys or Thr328Arg), a large deletion (deletion of 72 base pairs: c.971_1018 + 24del72*) or an 18-bp duplication in the F12 (FXII) gene are detected in HAE-FXII [3].

We describe a case series of nine HAE nC1-INH patients and Factor FXII mutation from two different families. All affected patients were women with a mean age at diagnosis of 43 years. Four were asymptomatic, while the others reported recurrent episodes of edema with facial and abdominal location. Trigger factors included medication (contraceptives such as drospirenone 3 mg and ethinyl estradiol 0.03 mg), pregnancy, trauma, and stress. The mean time to diagnosis since the first symptom was three years. Both families had Spanish origin.

**Materials and methods:** Serum, citrated plasma, and EDTA-blood were collected from the patients, and antigenic values of C4 and C1-INH were assayed by turbidimetric immunoassays in serum samples. Functional C1-INH activity was assayed by a chromogenic assay in plasma samples, and genetic testing for F12 was assayed by Sanger sequencing of exon 9 and intron–exon boundaries.

**Results:** The results of quantitative and qualitative C4, antigenic and functional C1-INH were normal. Genetic testing for F12 gene revealed that the patients were heterozygous for the common missense mutation c.983C > A (p.Thr328Lys). To date, 47 HAE nC1-INH patients in Argentina with FXII mutations have been diagnosed, and no other mutations have been described.

Specific treatments of bradykinin angioedema (icatibant, C1-INH concentrates and tranexamic acid) and nonspecific treatments (such as progestin) are effective in these patients [5].

**Conclusions:** We described a case series from two Argentinean families with mutations in the F12 gene. Interestingly, we found that all affected patients were women, clinical episodes were mild, and they were related to hormonal levels (oral contraceptives and pregnancy), stress, and trauma. All family members were less symptomatic than other types of HAE, and the delay in diagnosis was three years.

**Acknowledgments**

GRADAEH (Grupo Argentino de Estudio de Angioedema Hereditario)

Dr. Alejandro Berardi, Dr. Claudio Fantini, Dr. Dario Josviack, Dra. Mónica Marocco and Dr. Daniel Vazquez.

**References**

1. [1]Bork K. Diagnosis and treatment of Hereditary Angioedema with normal C1-inhibitor Allergy, Asthma & Clinical Immunology 2010, 6:15
2. [2]Bork K, Wulff K, Hardt J, et al. Hereditary Angioedema caused by missense mutations in the factor XII gene: clinical features, trigger factors, and therapy. J Allergy Clin Immunol. 2009;124(1):129‒134.
3. [3]Jyoti Sharma. Pathophysiology of Hereditary Angioedema (HAE) Beyond the SERPING1 Gene. Clin Rev Allergy Immunol. 2021 Jan 14. https://doi.org/10.1007/s12016-021-08835-8.
4. [4]Rosa Santacroce et al. The Genetics of Hereditary Angioedema: A Review. Clin. Med. 2021, 10, 2023. https://doi.org/10.3390/jcm10092023
5. [5]A. Deroux et al Hereditary Angioedema with normal C1-inhibitor and factor XII mutation: a series of 57 patients from the French National Center of Reference for Angioedema. Clinical and Experimental Immunology, 185: 332–337.

## O-28 Characteristics of Acquired Angioedema due to C1-inhibitor deficiency (AAE-C1-INH) at a large tertiary care hospital in Spain

### Patricia Mir-Ihara^1,^*, Ana Nin-Valencia^1^, Isamar De Agrela Méndes^1^, Itsaso Losantos^2^, Pilar Nozal^3,4,5,6^, Ana Entrala^1,4^, Teresa Caballero^1,4,5,6^

#### ^1^Allergy Department, Hospital Universitario La Paz, Madrid, Spain; ^2^Biostatistical Department, Hospital La Paz Health Research Institute (IdiPaz), Madrid, Spain; ^3^Immunology Unit, Hospital Universitario La Paz, Madrid, Spain; ^4^Hospital La Paz Health Research Institute (IdiPaz), Madrid, Spain; ^5^Centre for Biomedical Research Network on Rare Diseases (CIBERER) U754, Hospital Universitario La Paz, Madrid, Spain; ^6^CSUR de Angioedema Hereditario del Hospital Universitario La Paz, Madrid, Spain

##### *Correspondence: patriciakmir@gmail.com

*Allergy, Asthma & Clinical Immunology* 2023, **19**(Suppl 1):O-28

**Background:** Acquired Angioedema with C1-inhibitor deficiency (AEA-C1-INH) is a rare form of angioedema characterized by recurrent attacks of localized edema. The aim of this study was to describe the characteristics of patients with AEA-C1-INH.

**Methods:** A retrospective observational study was performed. Data were collected from all patients with a diagnosis of acquired C1-INH deficiency under follow-up at our center from 1999 to 2021. The data was updated as of December 31, 2021. The study was approved by the Ethics Committee (PI-4598).

**Results:** Fifteen cases were included (7 females, 8 males), four being asymptomatic. The median age of symptom onset was 44 years (P25–P75:41–52) and the median time between symptom onset and diagnosis (diagnosis delay) was 1.2 years (P25–75 0.7–3.2) (n = 11).

Complement study at diagnosis can be seen in Table 1. *SERPING1* gene study was negative in 10 patients.

The characteristics of 676 angioedema attacks from 10 patients were collected. Median number of AE attacks was 25.5 (P25–P75: 12–131). Most attacks affected an only location (n = 553, 82.8%) (abdominal 32.7%; peripheral 25.4%, cervicofacial 16.4%; genital 4.4%; upper airways 2,5%; other 0.3%), whereas a high percentage of attacks affected multiple locations (n = 123; 18.2%) (different peripheral locations 44.7%; peripheral + abdominal 27.6%; peripheral + cervicofacial 8.9%; abdominal + cervicofacial 5.7%; other combinations 13.0%).

Ten patients had used on-demand treatment for angioedema attacks including IV plasma derived C1-INH (pdC1-INH) (n = 4) and/or SC icatibant acetate (n = 8). Long-term prophylaxis was performed by 5 patients: oral tranexamic acid (n = 3), stanozolol (n = 1), danazol (n = 2), IV pdC1-INH (n = 2), SC pdC1-INH (n = 1). IV pdC1-INH was used for short- term prophylaxis when indicated. Rituximab was used to treat angioedema in one patient with a high decrease in the number of AE attacks and for hematological disorders in two patients. Rituximab was proposed to 3 other patients with a high frequency of AE attacks and rejected by 2 of them because of fear of secondary effects.

**Conclusion:** The disease activity in AEA-C1-INH is very different among patients. C1q may be in the normal range at diagnosis. AutoC1-INHAbs and C1-INH-antiC1-INHAb immunocomplexes are important for diagnosis. The most frequent only location of AE attacks is abdominal. Rituximab is a treatment option when the frequency of AE attacks is high.

**Table 1 (abstract O-28):** Laboratory study at diagnosis

Patient number	Plasma C1-INH function (> 50%)	Antigenic C1-INH (16.0–33.0 mg/dL)	Serum C4 levels (14.0–60.0 mg/dL)	Serum C1q levels (> 100 µg/dL)	Autoantibodies anti C1INH (antiC1INHAbs)	Immunocomplexes C1INH–antiC1INHAb	SERPING1 gene study
1	50	4.8	1.67	60	ND	ND	ND
2	10.4	27.5	7.71	60	ND	ND	ND
3	11	9.8		138	ND	ND	ND
4	40	9.89	2	58	Negative	ND	ND
5	18	2.8	1.4	11.5	IgG	ND	ND
6	14.06	17.9	16.4	120	Negative	ND	Negative
7	25.67	16.2		157.95	IgG	ND	Negative
8	6	15	1.56	0	IgA	Negative	Negative
9	10.77	5.82	1.56	0	IgA, IgG	ND	Negative
10	21.61	13	35.9	233.16	Negative*	Negative*	Negative
11	33.46	12.1	1.66	32.13	Negative	IgG	Negative
12	19.64	4.62	5.32	111.88	IgA, IgM	Negative	Negative
13	25.96	3.99	1.5	71.82	Negative	IgA, IgG	Negative
14	11.38	5.49	3.53	58.11	IgM	Negative	Negative
15	7.9	5.24	ND	115.58	IgG	Negative	Negative

ND: Not done.

*Patient 10 had positive autoC1-INHabs several years later.

## O-29 Angioedema due to acquired C1-inhibitor deficiency associated to monoclonal gammopathies of undetermined significance: characteristics of a French national cohort

### Constance Lahuna^1^, Federica Defendi^2^, Laurence Bouillet^3^, Isabelle Boccon-Gibod^3^, David Launay^4^, Arsene Mekinian^5^, Olivier Fain^5^, Delphine Gobert^5^

#### ^1^Internal Medicine Department, University Hospital, Martinique; ^2^Immunology Laboratory, University Hospital, Grenoble, France; ^3^Internal Medicine Department, University Hospital, Grenoble, France; ^4^Internal Medicine Department, Claude-Huriez University Hospital, Lille, France; ^5^Internal Medicine Department, Saint Antoine University Hospital, Assistance Publique-Hôpitaux de Paris, Sorbonne Université, DHU i2B, Paris, France

*Allergy, Asthma & Clinical Immunology* 2023, **19**(Suppl 1):O-29

**Background:** No specific description of monoclonal gammopathies of undetermined significance (MGUS)-associated Acquired Angioedema (AAE) has been reported yet.

**Objective:** Describe the biological and clinical characteristics, evolution and response to treatment of MGUS-associated C1 inhibitor (C1INH)-Acquired Angioedema.

**Material and methods:** We conducted a French national retrospective observational study on MGUS-associated Acquired Angioedema spanning a 30-year period.

**Results:** 41 patients with MGUS-associated C1INH-Acquired Angioedema at diagnosis were included; 70% were associated to anti-C1-INH antibodies (Table 1). Median age at first angioedema attack was 64 years and at diagnosis 66 years. 88% of patients benefited from acute attack treatments, and 77% from long-term prophylaxis, either danazol, tranexamic acid or lanadelumab. Median follow-up was 7 years, during which 14 patients (33%) evolved into well-defined malignant hemopathies. 50% of patients were given a hematological treatment, either rituximab alone, indicated by Acquired Angioedema, or validated combinations of chemotherapies, indicated by evolution into a lymphoma or myeloma. 15 patients (35%) were in complete remission at last visit, of which 60% were in complete remission of serum monoclonal immunoglobulin.

**Conclusion:** Complete Acquired Angioedema remission is correlated to remission of serum monoclonal immunoglobulin. In our MGUS-associated C1INH-Acquired Angioedema cohort, we recorded an incidence of evolution into malignant hemopathies of 4% per patient-year. It is therefore crucial to conduct full hematological workup during follow-up, especially if AAE relapses or acute attacks frequency increases, at an annual rhythm.

**Table 1 (abstract O-29):** Clinical and biological characteristics of AAE and MGUS

	N	IgM	IgG	IgA	Total
MIg isotype, n (%)	41	24 (58%)	11 (27%)	6 (15%)	41
Mean mIg value, n (g/L) (SD)	40	4 (4.2)	6.7 (6.3)	3.8 (1.7)	4,8 (4,8)
Sex ratio, F:M	41	1/1	0,72/1	2/1	1/1
Mean age at 1st AE attack y (SD)	41	64 (12)	58 (11)	68 (17)	63 (13)
Mean diagnostic delay, y (SD)	41	3 (4)	3 (5)	1 (1)	3 (4)
Anti-C1INH Ab presence	41	15 (63%)	7 (64%)	6 (100%)	28 (68%)
Anti-C1INH Ab isotype	41	10 IgM 5 IgG	7 IgG	6 IgA	6 IgA 10 IgM 12 IgG
Low C1INH antigen	40	22 (92%)	8 (80%)	5 (83%)	35 (87%)
Low C1q	34	15 (94%)	5 (55%)	4 (100%)	29 (85%)
Low C4	41	24 (100%)	9 (82%)	6 (100%)	39 (95%)
Evolution into malignant hemopathy	41	7	6	1	14 (34%)
Mean delay between AAE diagnosis and evolution into malignant hemopathy (years)	14	7	11	4	9

*N* number of patients with available data, *Low* biological value under 50% of the reference value for laboratory

## O-30 A novel diagnostic parameter for acquired C1-inhibitor deficiency

### Zsófia Pólai^1^, Erika Kajdácsi^2^, László Cervenák^2^, Zsuzsanna Balla^1^, Szabolcs Benedek^2^, Lilian Varga^1^, Henriette Farkas^1,^*

#### ^1^Hungarian Angioedema Center of Reference and Excellence, Department of Internal Medicine and Haematology, Semmelweis University, Budapest, Hungary; ^2^Department of Internal Medicine and Haematology, Semmelweis University, Budapest, Hungary

##### *Correspondence: farkas.henriette@med.semmelweis-univ.hu

*Allergy, Asthma & Clinical Immunology* 2023, **19**(Suppl 1):O-30

**Background:** Autoantibodies against C1-inhibitor (C1-INH-Ab) have a diagnostic value in Acquired Angioedema due to C1-inhibitor deficiency (C1-INH-AAE), even though antibodies can circulate in complexes, which can be undetectable by proven methods. C1-INH-AAE often accompanies lymphoproliferative underlying diseases, which can be the cause of C1-INH-Ab production. Our aim was to measure C1-INH/C1-INH-Ab complexes (CAC) and investigate their connection to clinical symptoms, C1-INH-Ab and the changes in their titer over time.

**Materials and methods:** In the past 30 years, out of the 3938 patients sent to the Hungaria Angioedema Center of Reference and Excellence with angioedema symptoms. All patients were followed-up and complement parameters were measured frequently.

**Results:** 19 patients were diagnosed with C1-INH-AAE in our Center; 79% of them had an underlying disease. Samples were examined with a newly developed in-house complex ELISA method. Patients with high C1-INH-Ab titer had a CAC titer which did not exceed the normal level and the ones with high CAC titer had a C1-INH-Ab titer which did not exceed the normal level. In case of those patients who had C1-INH-Ab and CAC of the same type of immunoglobulin, the increasing titer of C1-INH-Ab went together with the decreasing level of CAC and vice versa.

**Conclusions:** CAC measurements explained the ineffectiveness of long term profilactic treatment and the need of unusually high dose of acute treatment with C1-INH. Free circulating and complex antibodies are in a dynamically changing equilibrium. CAC measurements can help to predict the development of an underlying disease. The efficiency of the treatment for underlying disease can be monitored by the decreasing CAC titers. Our results show that the CAC can be of important additional information besides the complement panel examination in case of C1-INH-AAE. Measurement of CAC is recommended to be done parallelly with C1-INH-Ab, so as to detect both free and bound antibodies.

## O-31 Long-term prophylaxis with Lanadelumab for children and adolescents with HAE

### Ekaterina A. Viktorova^1^, Natalya B. Kuzmenko^1^, Yuliya Rodina^1^, Anna A. Mukhina^1^, Elena A. Latysheva^2^, Irina A. Manto^2^, Tatiana V. Latysheva^2^, Anna Shcherbina^1^

#### ^1^Dmitry Rogachev National Research Center of Pediatric Hematology, Oncology and Immunology, Ministry of Health of the Russian Federation, Russian Federation; ^2^National Research Center—Institute of Immunology Federal Medical-Biological Agency of Russia, Moscow, Russian Federation

*Allergy, Asthma & Clinical Immunology* 2023, **19**(Suppl 1):O-31

**Introduction:** HAE is a rare, debilitating and potentially life-threatening condition that causes unpredictable angioedema attacks that may occur early in childhood.

The management of pediatric HAE patients differs in many ways from the management of adults, however, the concept of achieving maximum remission and therefore improving quality of life is fundamental to both groups. It is not possible to achieve remission without use of long-term preventive treatment. One of the recently approved products for pediatric patients (from 12 years old in Europe and the Russian Federation, from 2 years old in the USA) for long-term prevention is lanadelumab.

**Objective:** to evaluate the efficacy and safety of lanadelumab in pediatric patients including children younger than 12 years.

**Materials:** 14 HAE type 1 patients, aged 9 to 17 years (Me 13.3) (M:F 1:1), who received lanadelumab therapy were included in the study. Efficacy was assessed by comparing the frequency and severity of angioedema attacks before and during lanadelumab treatment. The Peds QL scale adapted for pediatric patients was used to assess the quality of life. Safety was assessed by recording adverse events and evaluation of their severity.

**Results:** Prior to initiation of lanadelumab therapy, 57% of patients were receiving C1 esterase inhibitor concentrate as a long-term prophylaxis. Difficult venous access in pediatric patients and living in a remote area from a medical organization were the factors that impacted patients’ compliance to C1 esterase inhibitor concentrate therapy.

The average frequency of angioedema attacks before initiation of lanadelumab therapy was 5.6 episodes per patient per month [median (minimum–maximum) 5 (4–10)]. Six patients received lanadelumab therapy outside off-label in relation to the patient's age. After 6 months on lanadelumab therapy, this indicator decreased to 0.7 episodes [median (minimum–maximum) 0 (0–5), p < 0.001]. In 7 patients, no attacks were recorded during the 6 months of the therapy. In one patient, there was a complete lack of therapy effect registered. The average value of quality-of-life indicators according to the Peds QL questionnaire improved significantly, median increased from 50 to 75 points (p < 0.003). There were no serious adverse events associated with lanadelumab treatment. The most common side effects seen were injection site reactions (pain, redness).

**Conclusion:** according to the results of the study, it was found that long-term prophylactic therapy with lanadelumab is safe and effective in children, including under the age of 12, and leads to improved quality of life.

## O-32 Is pre-procedural prophylaxis needed in patients receiving the newer HAE-C1-INH prophylactic therapies?

### Mar Guilarte^1,2^,*, Krasimira Baynova^3^, Ramón Lleonart-Bellfill^4^, Ethel Ibañez^5^, Ramón Almero^5^, Johana Gil-Serrano^1,2^, Pilar Sánchez-Payá^6^, Eugenia Sanchís^6^, María Luisa Baeza^7^, Lucía Ferrer^8^, Sergio Porcel^9^, Tatiana Navarro^10^, Stefan Cimbollelk^3^, Carmen Marcos^11^, María Dolores Del-Pozo^12^, Carmen Díaz-Donado^13^, Irene García-Gutierrez^14^, Ariel Callero^15^, María Cruz Torres-Gorriz^16^, Virginia Rodríguez^17^, Teresa Caballero^10^, on behalf of the Spanish Angioedema Committee from SEAIC (Spanish Society of Allergy and Clinical Immunology)

#### ^1^Allergy Department, Hospital Universitari Vall d’Hebron, Barcelona, Spain; ^2^Vall d’Hebron Research Unit, VHIR, Barcelona, Spain; ^3^Allergy Department, Hospital Universitario Virgen del Rocío, Sevilla, Spain; ^4^Allergy Department, Hospital Universitari de Bellvitge, L’Hospitalet, Spain; ^5^Allergy Department, Hospital Universitari la Fe, Valencia, Spain; ^6^Allergy Department, Hospital Universitario Rio Hortega, Valladolid, Spain; ^7^Allergy Department, Hospital Universitario Gregorio Marañon, Madrid, Spain; ^8^Allergy Department, Hospital Universitario Lozano Blesa, Zaragoza, Spain; ^9^Allergy Department, Hospital San Pedro de Alcántara, Cáceres, Spain; ^10^Allergy Department, Hospital Universitario La Paz, IDIPAZ, Madrid, Spain; ^11^Allergy Department, Complejo Hospitalario Universitario De Vigo, Vigo, Spain; ^12^Allergy Department, Hospital Universitario San Pedro, Logroño, Spain; ^13^Allergy Departmnet, Hospital Universitario Central de Asturias, Oviedo, Spain; ^14^Allergy Department, Hospital Marqués de Valdecilla, Santander, Spain; ^15^Allergy Department, Hospital Universitario Virgen de la Candelaria, Tenerife, Spain; ^16^Allergy Department, Hospital General Universitario de Castellón, Spain; ^17^Allergy Department, Hospital Clínico Universitario de Santiago, Santiago de Compostela, Spain

##### *Correspondence: mar.guilarte@vallhebron.cat

*Allergy, Asthma & Clinical Immunology* 2023, **19**(Suppl 1):O-32

**Introduction:** Short-term or pre-procedural prophylaxis (STP) is appropriate for patients anticipating situations that might precipitate an HAE attack such as dental, surgical and medical procedures, especially those with mechanical impact to the upper aerodigestive tract. HAE guidelines recommend the use of intravenous pdC1-INH as a 1rst line for STP. However, it is unknown whether STP is still necessary in those patients under the newest long-term prophylaxis (LTP) therapies that have achieved a complete disease control.

**Aim:** To explore the use of pre-procedural prophylaxis (STP) in HAE-C1-INH patients using a long-term prophylaxis (LTP) with new therapies, such as berotralstat, lanadelumab and subcutaneous (SC) pdC1-INH.

**Methods:** A survey was conducted by the Spanish Angioedema Group to assess the number of HAE-C1-INH patients using lanadelumab or SC pd-C1-INH who underwent any dental, surgical or medical procedure from April 2020 (when new LTP therapies were available in Spain) to January 2023. Demographics, treatment and dose used for of LTP, dental, medical or surgical procedures, STP used and time since the last LTP therapy administration were recorded retrospectively.

**Results:** A total of 108 patients that were under an LTP with new therapies from May 2020 to January 2023 were included. Thirty-seven patients, 25 females and 44.23 (range 22–75) years old, underwent a total of 43 dental, surgical, or medical procedures. Lanadelumab was used in 25 (54%) and SC pd-C1-INH in 20 (41%) of these patients. A STP was administered in 22 (51%) of the procedures, however 14 patients underwent 21 (49%) procedures without a STP. None of them developed HAE attacks during or after the procedure. The interventions performed without STP were 10 exondontias, 2 endodontias, 2 dental implants, 2 dental fillings, 2 esophagogastroduodenoscopies, one lumpectomy, one hemorrhoidectomy and one inguinal herniorrhaphy. No differences regarding complexity of the procedures were observed in patients that received a STP.

**Conclusions:** New LTP therapies may be safe to prevent procedural-related HAE attacks. Nevertheless, more data is needed to explore if SPT is still mandatory in patients under innovative LTP therapies, and specially in patients achieving control of the disease.

## O-33 Quality of life, disease control and mental health in patients with Hereditary Angioedema in Slovakia—national online survey

### Miloš Ješenak^1,2,3^, Katarína Hrubiskova^4^, Martin Suchansky^5^, Martina Ondrusova^1^

#### ^1^National Centre for Hereditary Angioedema, Department of Paediatrics, Jessenius Faculty of Medicine in Martin, Comenius University in Bratislava, University Teaching Hospital in Martin, Slovakia; ^2^National Centre for Hereditary Angioedema, Department of Pulmonology and Phthisiology, Jessenius Faculty of Medicine in Martin, Comenius University in Bratislava, University Teaching Hospital in Martin, Slovakia; ^3^Department of Clinical Immunology and Allergology, University Teaching Hospital in Martin, Slovakia; ^4^National Centre for Hereditary Angioedema, Department of Internal Medicine, Faculty of Medicine, Comenius University in Bratislava, University Teaching Hospital in Bratislava, Slovakia; ^5^PHARM-In Ltd., Bratislava, Slovakia

*Allergy, Asthma & Clinical Immunology* 2023, **19**(Suppl 1):O-33

**Background**: Hereditary Angioedema (HAE) is associated with a significant disease burden and it strongly affects the quality of life of the affected patients and their relatives. The evaluation of QoL and specific impact of HAE on life could guide the decisions in the personalized management of HAE.

**Patients and Methods**: By the end of 2022, 126 living patients with HAE were identified in Slovakia. During the period 1.7.2022–30.9.2022 we performed the online survey among our HAE patients. We were focused on the disease control, quality of life and the impact on mental health using three standardized questionnaires: AE-QoL (Angioedema Quality of Life Questionnaire), AECT (Angioedema Control Test) and HADS (Hospital Anxiety and Depression Scale).

**Results**: All together, 68 patients (54% of all HAE patients; 42% females) were included and fulfilled the questionnaires. 45 patients (66.18%) were treated with long-term prophylaxis (18 danazol, 8 berotralstat, 4 tranexamic acid, 13 lanadelumab and 2 pdC1-INH concentrate). 9 patients (13.24%) had asymptomatic disease, 21 (30.88%) mild, 36 (52.94%) moderate and 2 (2.94%) severe form of HAE. 13 patients used a rescue therapy during 4 weeks before the survey. Based on AE-QoL—7 patients declared decreased QoL. Females showed better functioning in life compared to males. Patients treated with lanadelumab declared the best functioning compared to the other LTP modalities. Patients without LTP yielded better mood and lower fatigue compared to subjects with LTP. Fear and shame were declared by 12 patients. The disease was well controlled in 61 (89.71%) patients. Patients treated with lanadelumab has the best disease control compared to the other LTP tools. Altogether, 13.24% patients declared anxiety and 8.82% depression of various degree. Although patients with lanadelumab showed the highest level of anxiety, the depression prevalence was low and comparable with the patients without LTP.

**Conclusions**: HAE patients in Slovakia are successfully treated and managed according to the international guidelines. Our HAE patients’ cohort is well controlled and the disease control is even better compared to the published data from the other countries. HAE affects significantly selected domains of QoL. However, modern tools of LTP—especially lanadelumab, could significantly improve various aspects of QoL and prevent the negative impact on mental health of the affected patients.

## O-34 Effectiveness of an adapted treatment schedule for long-term prophylaxis in patients with HAE-C1-INH

### Johana Gil-Serrano^1^, Anna Sala-Cunill^1,2^, Moises Labrador-Horrillo, Paula Galvan Blasco^1,2^, Victoria Cardona^1,2^, Olga Luengo^1,2^, Javier Pereira, Mar Guilarte^1,2,^*

#### ^1^Allergy Section, Internal Medicine Department, Hospital Universitari Vall d’Hebron, Barcelona, Spain; ^2^HAE Spanish Reference Center (CSUR); ^3^Allergy Research Unit, Vall d’Hebron Research Institute (VHIR), Barcelona, Spain

##### *Correspondence: mar.guilarte@vallhebron.cat

*Allergy, Asthma & Clinical Immunology* 2023, **19**(Suppl 1):O-34

**Background:** Hereditary Angioedema due to deficient C1-inhibitor (HAE-C1-INH) is a rare disease in which the AE episodes are due to the activation of the contact system and the secondary production of bradykinin. Current prophylactict approach is focused either on the replacement of the C1-inhibitor or interfering in the cascade of the production of the bradykinin. The aim of this study is to describe our experience in a single center with new available prophylactic therapies and the use of a modified regimen treatment with Berinert 2000UI and Lanadelumab in a single hospital.

**Material and methods:** We performed a retrospective study from January 2020 until January 2023 in which we included all patients HAE-C1-INH that were under long-term-prophylaxis treatment with SC pdC1-INH and/or lanadelumab. Demographics, comorbidities, adapted regimen treatment and disease control/quality of life (HAE-QoL, AAS, AECT, AE-QoL) was assessed. Our adapted treatment schedule was: lanadelumab was started at doses of 300 mg every 15 days during 3 months (6 doses), then 300 mg every 3 weeks (2 doses) and finally maintenance dose of 300 mg every 4 weeks and SC pdC1-INH was administered on a fixed dose of 2000UI twice a week (following the GEAB (Spanish Group for the Study of Angioedema Mediated by bradykinin) protocol.

**Results:** A total of 14 patients were under a long term prophylaxis with both SC pdC1NH (57%) or lanadelumab (43%). Eleven patients (79%) were women with a mean age of 41.4 (range 24–61 years). Patients with HAE-C1-INH type 1 represent 93% (n = 13) and HAE-C1-INH type 2 the 7% (n = 1). Those patients receiving SC pdC1-INH, 5 achieved complete disease control with the regimen (4000Ui/week) and in 3 of them required a dose increase (6000Ui/week). Nevertheless, these increase was not enough and a switch to lanadelumab was required. In all patients receiving lanadelumab, a complete control of disease was obtained in 8/9 patients after the first administration. Lanadelumab adapted protocol was possible in all except 1 of the patients.

**Conclusion:** The use of an adapted treatment schedule in patients with HAE-C1-INH with lanadelumab and/or Sc pdC1-INH is effective to achieve a complete control of the disease in most of the cases. This can be a useful to minimize hospital visits, treatment costs and to improve patient’s quality of life.

## O-35 Lanadelumab effectiveness and safety regardless of dosing and dosing changes in patients with Hereditary Angioedema from the United States and Canada: Real-world evidence from the EMPOWER Study

### Stephen Betschel^1,^*, Paula J. Busse^2^, Aleena Banerji^3^, Daniel Petroni^4^, John Anderson^5^, Daniel Nova Estepan^6^, Jaco Botha^7^, Krystal Sing^8^, Salomé Juethner^8^

#### ^1^Clinical Immunology and Allergy, University of Toronto, Toronto, ON, Canada; ^2^Division of Allergy and Clinical Immunology, Icahn School of Medicine at Mount Sinai, New York, NY, USA; ^3^Division of Rheumatology, Allergy and Immunology, Department of Medicine, Massachusetts General Hospital, Harvard Medical School, Boston, MA, USA; ^4^Seattle Allergy and Asthma Research Institute, Seattle, WA, USA; ^5^AllerVie Health, Birmingham, AL, USA; ^6^Takeda Development Center Americas, Inc., Lexington, MA, USA; ^7^Takeda Pharmaceuticals International AG, Zurich, Switzerland; ^8^Takeda Pharmaceuticals U.S.A., Inc., Lexington, MA, USA

##### *Correspondence: stephen.betschel@unityhealth.to

*Allergy, Asthma & Clinical Immunology* 2023, **19**(Suppl 1):O-35

**Background:** Real-world effectiveness and safety of lanadelumab according to dosing frequency was analysed in a subanalysis of the Phase IV observational, non-interventional, multicenter EMPOWER Study (NCT03845400).

**Methods:** Patients with Hereditary Angioedema (HAE) Type I/II aged ≥ 12 years were enrolled. Prevalent lanadelumab users (≥ 4 lanadelumab doses prior to enrolment) were divided into subgroups by lanadelumab dosing [300 mg every 2 weeks (Q2W), 300 mg every 4 weeks (Q4W), and dosing frequency reduction from 300 mg Q2W to 300 mg Q4W (Q2W to Q4W)]. Data on HAE attack rates and treatment-emergent adverse events (TEAEs), excluding HAE attacks, were collected.

**Results:** Data were collected from March 30, 2019 to March 1, 2022. A total of 79 (Q2W to Q4W subgroup: 7, Q2W subgroup: 61, Q4W subgroup: 11) patients were included. The mean ± SD age was 44.7 ± 17.6 years in Q2W to Q4W subgroup, 43.2 ± 17.3 years in Q2W subgroup, and 40.5 ± 19.6 years in Q4W subgroup. Most patients were female (Q2W to Q4W subgroup: 85.7%, Q2W subgroup: 68.9%, Q4W subgroup: 72.7%), White (Q2W to Q4W subgroup: 85.7%, Q2W subgroup: 93.4%, Q4W subgroup: 100%) and had any previous medical history events in the 6 months prior to enrolment (Q2W to Q4W subgroup: 71.4%, Q2W subgroup: 77.0%, Q4W subgroup: 54.5%). Patients from Q2W to Q4W subgroup spent a mean ± SD of 6.5 ± 4.5 months on Q2W dosing before switching to Q4W dosing for a mean ± SD of 15.4 ± 7.7 months. Patients from Q2W and Q4W subgroups received lanadelumab for a mean ± SD of 23.4 ± 9.1 and 23.9 ± 3.7 months, respectively. Mean (95% CI) HAE attack rates were low during both dosing phases in patients from Q2W to Q4W subgroup (0.04 [0.01–0.12] attacks/month on Q2W dosing and 0.01 [0.00–0.04] attacks/month on Q4W dosing); in Q2W and Q4W subgroups, HAE attack rates were 0.26 (0.24–0.29) attacks/month and 0.08 (0.05–0.11) attacks/month, respectively. A total of 80 TEAEs were reported in 33 patients; most were mild/moderate in severity (93.8%) and non-serious (95.0%). No lanadelumab-related TEAEs led to discontinuation from the study.

**Conclusions:** Real-world evidence from the EMPOWER Study shows low HAE attack rates on lanadelumab treatment regardless of dosing frequency. Most TEAEs were mild/moderate in severity and non- serious. These data suggest that in selected patients with HAE, lanadelumab dosing may be reduced without compromising effectiveness and safety.

**Trial registration**

www.clinicaltrials.gov NCT03845400

## O-36 HAE patients decision to carry on-demand treatment when away from home

### Stephen Betschel^1^, Sally van Kooten^2^, Markus Heckmann^3^, Sherry Danese^4^, Ledia Goga^5^, Mar Guilarte^6^

#### ^1^Division of Allergy and Immunology, Department of Medicine, St. Michael's Hospital, University of Toronto, Toronto, ON, Canada; ^2^KalVista Pharmaceuticals, Inc., Cambridge, MA, USA; ^3^KalVista Pharmaceuticals, Inc. Cambridge, MA, USA; ^4^Outcomes Insights, Agoura Hills, California, USA; ^5^KalVista Pharmaceuticals, Inc., Cambridge, MA, USA; ^6^Allergy Section, Medicine Department, Hospital Universitari Vall d’Hebron, Vall d’Hebron Research Institute (VHIR), Barcelona, Spain

##### Email: etschels@smh.ca; svk@kalvista.com; sherry@outins.com; lgo@kalvista.com; mguilarte@gmail.com

*Allergy, Asthma & Clinical Immunology* 2023, **19**(Suppl 1):O-36

**Background:** Hereditary Angioedema (HAE) is characterized by recurrent, unpredictable episodes of subcutaneous or submucosal swelling. Guidelines recommend that all patients have sufficient medication for on‐demand treatment of at least two attacks and carry on‐demand medication at all times. This survey investigated the frequency with which HAE-C1-INH patients carry their on-demand treatment with them when away from home and the reasons for their decisions.

**Methods:** People living with HAE-C1-INH were recruited by the US Hereditary Angioedema Association (HAEA) to complete a 20-min, self-reported, online survey between September 6 and October 19, 2022.

**Results:** Respondents included 107 people with HAE; 80% female, 98% adults (≥ 18 years). Attack management included on-demand therapy only (50%, n = 53) or prophylaxis with on-demand therapy (50%, n = 54). Only one-third (36%) always carry their on-demand treatment with them when they leave home as part of their day-to-day life, a finding consistent amongst those taking on-demand treatment only (38%) and those on prophylaxis (35%). Overall, patients would travel 3.5 h (mean) from home without on-demand treatment, which included 2.5 h for those on prophylaxis and interestingly, those using on-demand treatment only (not on prophylaxis) would travel even further, 4.5 h, with 21% reporting travel > 6 h. When asked to provide reasons for not taking on-demand treatment with them, 72%, ‘prefer to administer treatment at home.’ Other reasons included, ‘on-demand treatment is bulky,’ (32%), ‘could trigger a security check,’ (29%), and is ‘embarrassing to carry,’ (13%). Overall, 44% reported they prefer to avoid potential attack triggers rather than carry on-demand treatment with them. While 77% of patients on prophylaxis state they are confident they will not have an attack and therefore do not carry their on-demand treatment when away from home, 43% also avoid potential attack triggers.

**Conclusions:** These results highlight that the majority of HAE patients often choose not to carry on-demand treatment with them when they are away from home, missing the opportunity to treat attacks early and optimize treatment outcomes. Given current on-demand treatment options, HAE patients prefer to avoid triggers and treat at home rather than carry on-demand treatment with them. Interestingly, this was also true for patients on modern prophylactic treatment. The promise of self-administered, on-demand therapy, leading to better attack management, has not been fulfilled. New treatment options are necessary to enable better management of attacks and encourage compliance in carrying on-demand treatment when, as part of day-to-day life, patients are away from home.

## O-37 The ACARE network—An update on global angioedema projects and initiatives

### Marcus Maurer^1,2^, on behalf of the GA2LEN/HAEi network of Angioedema Centers of Reference and Excellence (ACARE)

#### ^1^Angioedema Center of Reference and Excellence (ACARE), Institute of Allergology, Charité—Universitätsmedizin Berlin, corporate member of Freie Universität Berlin and Humboldt-Universität zu Berlin, Berlin, Germany; ^2^Fraunhofer Institute for Translational Medicine and Pharmacology ITMP, Allergology and Immunology, Berlin, Germany

*Allergy, Asthma & Clinical Immunology* 2023, **19**(Suppl 1):O-37

The ACARE program is a joint initiative by GA^2^LEN (Global Allergy and Asthma European Network) and HAEi (Hereditary Angioedema International) with the aim of developing and accrediting an interactive network of centers of reference and excellence in angioedema management. Founded in December of 2019, the ACARE network has established itself on 6 continents with 79 member centers in 35 countries as of March 2023. Today it is the fastest growing and most active international consortium of angioedemologists and angioedema centers with the largest global reach to patients affected by angioedema and its comorbidities.

The ACARE network facilitates scientific projects and studies. We also inform and educate medical professionals on angioedema through our websites and social media channels, our education programs, as well as our meetings. Our co-initiator and partner, HAEi, is a global non-profit network of patient associations dedicated to improving the lives of people with HAE. They provide their member organizations with specially designed tools and technical assistance that promote disease education and support activities addressing the unique needs of HAE patients and their families.

Since our inception, we successfully implemented several educational programs. These were: The Power of Prophylaxis webinar series (3x), COVID-19 webinars (2x). Our ongoing formats are: INTERACT Masterclass on angioedema (3/4) and the Make a Difference webinar series (8/11).

In 2023 and 2024, the ACARE network will host three live preceptorships, of which one already took place in India in February 2023 and the second will take place in the Gulf region in the fall of this year.

This year, we are launching the ACARE LevelUp physician education program. It will include Journal Clubs (30), Webinars (6), Preceptorships (3), Newsletters (6), a Podcast series, a digital Masterclass and a corresponding Social Media campaign to share and amplify our content.

There are currently five Scientific Projects run through the ACARE network. One of these (IMAGINE 1.0) has ended, while three (IMAGINE 2.0, SHAERPA and DANCE) are ongoing. In addition, there are two crossCORE projects called PROMUSE and PROMUSE-PAT. Lastly, one more project (HAPY) is in the pipeline and will commence in mid-2023.

The Bradykinin Symposium/Angioedema School was first held in 2022 and will take place every two years. Lastly, the Global Angioedema Registry CARE is in development and will be based on the highly successful chronic urticaria registry CURE.

## O-38 The angioedema registry of the Italian Network for Hereditary and Acquired Angioedema (ITACA): a tool to monitor HAE course, therapeutic adherence and overall burden of the disease

### Mauro Cancian^1^, Riccardo Senter^1^, Paolina Quattrocchi^2^, Giuseppe Spadaro^3^, Paolo Borrelli^4^, Francesca Perego^5^, Vincenzo Margaglione^6^, Francesco Arcoleo^7^, Vincenzo Montinaro^8^, Massimo Triggiani^9^, Andrea Zanichelli^10^

#### ^1^Department of Systems Medicine, University Hospital of Padova, Padova, Italy; ^2^Department of Clinical and Experimental Medicine, University of Messina, Messina, Italy; ^3^Department of Translational Medical Sciences, University of Naples Federico II, Italy; ^4^SSD Dermatologia e Allergologia, Ospedale Beauregard, Aosta, Italy; ^5^Istituti Clinici Scientifici Maugeri IRCSS, Milan, Italy; ^6^Department of Clinical and Experimental Medicine, University of Foggia, Italy; ^7^Clinical Pathology Division, Ospedali Riuniti Villa Sofia-Cervello, Palermo, Italy; ^8^Miulli General Hospital, Acquaviva delle Fonti, Italy; ^9^Division of Allergy and Clinical Immunology, University of Salerno, Italy; ^10^IRCSS Policlinico San Donato, University of Milan, Italy

*Allergy, Asthma & Clinical Immunology* 2023, **19(Suppl 1): **O-38

**Background:** Rare disease registries connect affected patients, families and clinicians to capture as much detailed data as possible with the aim of improving disease knowledge, scientific research, new drug evaluation and therapeutic strategies by precision-medicine based criteria. An Italian Hereditary Angioedema (HAE) registry was established in 2015 and formed the embryo for the development, in 2017, of the HAE Global registry (HGR). Unfortunately, the initiative proved to be economically unsustainable and HGR closed in December 2020.

**Objectives and methods:** After the closure of HGR the Italian Network for Hereditary and Acquired Angioedema (ITACA, www.angioedemaitaca.org) decided to take over its original registry and chose as technical partner Burning Flame, which had developed the informatic tool at the beginning. The new project is based on BIF (Business Innovation Framework), Burning Flame’s proprietary framework created using PHP, Laravel and Javascript technologies and designed to guarantee high performance and safety standards. The database is hosted on cloud servers managed and monitored by the same IT company.

**Results:** The ITACA HAE registry, active again from February 2023, allows physicians to collect demographics and data on type of angioedema, comorbidities, frequency, characteristics and treatment of acute attacks, Emergency Room admissions, administration of short term prophylaxis, long-term prophylaxis regimen and many other clinical details. Patients can directly enter data on angioedema episodes and periodically fill out questionnaires related to the overall burden of the disease, including AE-Qol, BIS-11, SF-36. To verify the quality of the collected data, patients and authorized staff of the referral centers receive alerts at regular intervals asking them to check whether all clinical events in that period have been entered and verified. Each reference center has access to information on its cohort, and multiple centers can agree to share their data in an anonymized manner for scientific and epidemiological purposes.

**Conclusions:** The HAE ITACA registry allows to follow-up patients in real time, even during periods like the one just past when patient access to hospitals and direct contact with referring physicians were severely hampered by the Covid-19 pandemic. The Italian registry gives patients the feeling that they are always in close contact with HAE specialists, as indeed it is, and enables them to have an objective vision of how their situation is progressing over time. Finally, as ITACA owns the software and source code there is the possibility of providing this tool to other countries with quite affordable costs.

## O-39 The impact of puberty on C1-INH-HAE course: a survey from the Italian Network for Hereditary and Acquired Angioedema (ITACA)

### Riccardo Senter^1^, Maria Domenica Guarino^2^, Caterina Colangelo^3^, Marica Giliberti^4^, Luisa Brussino^5^, Donatella Bignardi^6^, Oliviero Rossi^7^, Davide Firinu^8^, Isabella Del Corso^9^, Paola Triggianese^10^, Mauro Cancian^1^

#### ^1^Azienda Ospedale, Università di Padova, Italy; ^2^Presidio Ospedaliero di Civitanova Marche, Italy; ^3^Ospedale Spirito Santo di Pescara, Italy; ^4^Azienda Ospedaliero, Universitaria di Bari, Italy; ^5^Azienda Ospedaliera Ordine Mauriziano di Torino, Italy; ^6^IRCCS San Martino, Genova, Italy; ^7^AOU Careggi, Firenze, Italy; ^8^AUO Cagliari, Italy; ^9^AOU Pisana, Italy; ^10^Policlinico Tor Vergata, Roma, Italy

*Allergy, Asthma & Clinical Immunology* 2023, **19**(Suppl 1):O-39

**Background:** Sex hormones are thought to play a significant role in the pathophysiology of C1-INH-HAE. In fact, females present a more severe disease activity and medications containing estrogens worsen frequency and intensity of attacks. Our study aimed to investigate clinical features in HAE-C1-INH-patients at the time of puberty, when sex hormones begin to excert their activity on growth and development.

**Materials and methods:** We performed a retrospective observational study on HAE-C1-INH-patients referring to 10 centers of the Italian Network for Hereditary and Acquired Angioedema (ITACA) included in the ITACA Registry. Subjects between 10 (if puberty already occurred) and 40 years old at the time of the interview were enrolled, after proper consent. Age of onset of puberty was established as the age at the first menstruation for females and the age at spermarche for males.

**Results:** 118 patients were included (60 F; 58 M). 95.8% of them were type 1 HAE. Median age at the time of the interview was 30 years with an Interquartile range (IQR) 13 without gender difference; median age at puberty was lower in females (12 years, IQR 1.5) than in males (13 years, IQR 2, p < 0.001). The proportion of symptomatic patients increased after puberty in both males (98.2% after vs 83.9% before puberty, p = 0.002) and females (96.3% after vs 68,4% before puberty, p < 0.001). The median of the reported monthly means of the acute attacks in the three years after puberty was 2 (IQR 2.17) in females, and significantly increased from the three years before puberty (0.4, IQR 2, p < 0.001). This increase was also observed in males (1.25, IQR 1.56 vs 1, IQR 1.92 respectively; p < 0.001). A greater proportion of females than males showed an increase of attacks after puberty (63.2% vs 41.2%, p = 0.022) and the amount of increase was higher in females (1, IQR 2) than in males (0, IQR 1, p = 0.02). Distribution of sites of acute attacks did not show a gender difference, neither before nor after puberty. In an univariate regression analysis, female gender predicted the worsening of symptoms after puberty (OR 2.44, S.E. 1.65–3.63, p = 0.02).

**Conclusion:** Our study described the impact of puberty on disease features in a large multicenter cohort of HAE-C1-INH-patients from the ITACA network, showing that puberty leads to an increased number of angioedema attacks and may be the moment of life when the disease starts to get worse for females.

## O-40 Comorbidities in angioedema due to C1-INH deficiency: a survey from the Italian Network for Hereditary and Acquired Angioedema (ITACA)

### Andrea Zanichelli^1,2^, Ricardo Senter^3^, Francesco Arcoleo^4^, Massimo Triggiani^5^, Vincenzo Montinaro^6^, Mauro Cancian^3^, on behalf of ITACA

#### ^1^Operative Unit of Medicine, Angioedema Center, IRCCS Policlinico San Donato, San Donato Milanese, Milan, Italy; ^2^Department of Biomedical Sciences for Health, University of Milan, Milan, Italy; ^3^Department of Systems Medicine, University Hospital of Padua, Padua, Italy; ^4^Azienda Ospedaliera Ospedali Riuniti Villa Sofia Cervello, Italy; ^5^Division of Allergy and Clinical Immunology, University of Salerno, Salerno, Italy; ^6^Ospedale Generale Regionale F. Miulli, Italy

*Allergy, Asthma & Clinical Immunology* 2023, **19**(Suppl 1):O-40

**Background**: Angioedema due to C1-inhibitor deficiency, hereditary (HAE) or acquired (AAE), is characterized by unpredictable attacks of swelling. C1-inhibitor (C1-INH) plays a pivotal role in several biological pathways.

**Objective**: We aimed to investigate the possible association of comorbidities with C1-INH deficiency and long-term prophylaxis (LTP) with androgens (AA) or tranexamic acid (TXA).

**Methods**: This retrospective cohort study involved adult patients with HAE or AAE referring to Milan and Padua angioedema centers in the period 1979–2021. A qualitative comparison was performed to analyze comorbidities vs. general population. The incidence of comorbidities was evaluated during AA or TXA vs. patients without LTP.

**Results**: A total of 500 patients were studied. A greater prevalence among patients was found for: heart diseases (10% vs. 4.8%), acute myocardial infarction (5.4% vs. 1.4%), HCV infection (9.6% vs. 2.5%), and appendectomy (16% vs. 4.3%). In patients with Acquired Angioedema, a greater prevalence was found for monoclonal gammopathy of unknown significance—MGUS (38.5% vs. 3.3%) and lymphoproliferative disorders (38.5% vs. 0.4%). In patients taking AA a greater incidence was found for: hypertension (22% vs. 11.1%; OR 1.89), hypercholesterolemia (18.8% vs. 4.7%; OR 3.97), diabetes mellitus (4.8% vs. 1.4%; OR 3.22), hepatic angioma (4.3% vs. 0.6%; OR 8.35), and focal nodular hyperplasia (2.4% vs. 0.4%; OR 6.9). No association with TXA and comorbidities was found.

**Conclusion**: In this large patient population with a rare disease followed for a 43-year period, we found a greater prevalence of comorbidities hitherto unreported in the literature and an association between comorbidities and LTP with AA.

## O-41 Pharmacoeconomic burden for HAE patients in Brazil

### Marina Teixeira Henriques^1^, Lucca Nogueira Paes Jannuzzi^2^, Gabriel Abila Gonçalves^2^, Solange Rodrigues do Valle^3^, Bianca de Souza Leite Sender^4^, Faradiba Sarquis Serpa^4^, Daniel Prado dos Santos^5^, Henrique Sarquis Serpa^6^, Sabrina Macely Souza dos Santos^7^, Dhallya Andressa da Silva Cruz^7^, Luciana Costa Pinto da Silva^8^, Victor Evangelista de Farias Ferraz^9^, Angelina Xavier Acosta^10^, Maria Denise Carvalho^11^, Isabella Cristina Amaral Dantas^8^, Vânia Mesquita Gadelha Prazeres^8^, Camila Azevedo^12^, Têmis Felix^13^, Anete Sevciovic Grumach.^1^

#### ^1^Clinical Immunology, Department of Clinical Medicine, University Center Faculty of Medicine ABC, Santo Andre, SP, Brazil; ^2^Graduate student of University Center FMABC, Santo Andre, SP, Brazil; ^3^HU Clementino Fraga Filho UFRJ, Rio de Janeiro, RJ, Brazil; ^4^Escola Superior de Ciências da Santa Casa de Misericórdia de Vitoria, Espírito Santo, ES, Brazil; ^5^Graduate student of Escola Superior de Ciências da Santa Casa de Misericórdia—EMESCAM, Vitória, ES, Brazil; ^6^Graduate student of University Center Multivix Vitória, ES, Brazil; ^7^Graduate student of Federal University of Amazonas, Manaus, AM, Brazil; ^8^Políclinica Codajás, Manaus, AM, Brazil; ^9^Clinical Hospital of Ribeirão Preto, Ribeirão Preto, SP, Brazil; ^10^University Hospital Prof. Edgar Santos, Salvador, BA, Brazil; ^11^University Hospital Walter Cantídeo, Fortaleza, CE, Brazil; ^12^Consultant of MAPE Solutions; ^13^Clinical Hospital of Porto Alegre—HCPA, Porto Alegre, RS, Brazil

*Allergy, Asthma & Clinical Immunology* 2023, **19**(Suppl 1):O-41

**Introduction:** Although it has been said that ‘health is priceless’, it certainly has its costs. In the last decades, the growing expenditure on health has raised concerns about better management of resources destined for this purpose. Among the factors related to the increase in health costs, changes in morbidity and mortality patterns in contemporary societies are evident, which can be explained by the reduction in infectious and contagious diseases and the increase in chronic degenerative ones. Hereditary Angioedema is a chronic disease that requires regular medical follow-up and treatment. The aim of this study was to evaluate the direct and indirect costs of Hereditary Angioedema with C1-inhibitor deficiency (C1-INH-HAE) treatment in specialized centers in Brazil.

**Methods:** The present study is part of the National Network for Rare Diseases. It has a prospective and retrospective design that aims to understand and measure the Care Value Journey (CVJ) of patients with C1-INH-HAE in Brazil. It was carried out in 116 patients of 7 reference centers. Questionnaires were applied to patients/caregivers, one retrospective and one prospective (v1), containing general data about the patient; diagnosis, treatment, productivity, costs and evolution of the disease. In addition, interviews were conducted with nurses, doctors and administrators allocated in the centers to understand the reality of the resources used within the protocols used for management of C1-INH-HAE in each institution.

**Results:** The most frequently used drug for long-term prophylaxis was oxandrolone [25/116; 21,5%], followed by danazol [14/116; 12.6%] and tranexamic acid [14/116; 12.06%]. The average monthly cost of oxandrolone was US$ 19.94; danazol, US$ 24.05 and tranexamic acid, US$ 8.61. Icatibant Acetate was the most commonly used medication for on demand treatment [14/116; 12.06%], with a mean use of 3 shots every 4 months for each patient. This drug was obtained through lawsuit and the cost per application was US$ 1,416.26. The total cost of this patient's care journey, including medical appointments, exams and medications was US$ 23,391.31 per year. Loss of productivity at work, which is an indirect cost, must also be taken into account in a pharmacoeconomic study. The time spent in a 1-year follow-up of a patient with C1-INH-HAE, taking into account medical appointments and exams, was 372.5 min. This time can reflect on absenteeism at work and school. The assessment of productivity loss, assessed using the Work Productivity and Activity Impairment—General Health (WPAI-GH) questionnaire, was answered by 7 caregivers and 87 patients. Of these, 46 patients and 4 caregivers answered that they were employed. The percentage of commitment during work (presenteeism) was 32.4% and the absenteeism (time lost from work) was 17.2%.

**Conclusion:** The direct and indirect costs that patients with C1-INH-HAE have with the treatment of the disease are excessively high considering the socioeconomic level of Brazilian population. However, the need of better access to therapy is essential. More investments in pharmacoeconomic studies are needed to think of strategies that minimize these costs.

## O-42 Management of Hereditary Angioedema with normal C1-INH: About a series of 149 French cases

### Laurence Bouillet^1,2^, Aurélie Du Than^2^, Delphine Gobert^2^, Isabelle Boccon-Gibod^2^, Laurent Sailler^2^, Stéphane Gayet^2^, Pierre Yves Jeandel^2^, Marie Caroline Taquet^2^, Sophie Debord^2^, Catherine Mansard^2^, David Launay^2^

#### ^1^French National Reference Center for Angioedema, Grenoble University Hospital, Grenoble, France; ^2^CREAK, France

*Allergy, Asthma & Clinical Immunology* 2023, **19**(Suppl 1):O-42

**Introduction:** Hereditary Angioedema with normal C1-INH (HAE-nC1-INH) are very rare diseases. The diagnosis is genetic. The two most frequent mutations are FXII and PLG. The KNG mutation is rarer. Their management is similar to that of AEH with C1-INH deficiency but without evidence based medicine. CREAK has identified all cases of AEH in order to evaluate their therapeutic management.

**Materials and methods:** This is a national retrospective study conducted in the CREAK network. Any patient identified with an FXII, PLG and KNG mutation was included.

**Results:** 149 patients were included, 27% with the PLG mutation and one case with the KNG mutation. 81,4% were women. The average age of the first symptom is 23 years. In 54% of cases, the pill or pregnancy was the revealing factor. 50% of women who have had a pregnancy have seen their disease worsen. At least one laryngeal crisis occurred in 43% of patients. 39% of women received C1-INH concentrate as attack treatment during pregnancy: the treatment was effective and well tolerated in all of them. 27 patients used icatibant for at least one attack: the treatment was effective and well tolerated in all patients. 35 patients required long-term treatment: 62.5% received tranexamic acid, which was effective in 50% of cases; 7 received lanadelumab, fully effective in 83% of the cases; 1 patient improved with berotralstat. For contraception: 27 women were taking a microprogestin pill; 7 an intra uterin advice.

**Conclusion:** This is an important French series. The evaluation of the management shows that treatments targeting the kallikrein kinin pathway (C1-INH concentrate, ictibant) are effective and safe. This is, of course, a retrospective study that needs to be confirmed prospectively.

## O-43 Diagnostic criteria of Angiotensin Converting Enzyme inhibitors (ACEi) induced bradykinin angioedema: The experience from the French national reference center for angioedema

### Alexis Bocquet, Nicolas Marmion, Catherine Mansard, Isabelle Boccon-Gibod, Laurence Bouillet

#### CREAK, France

*Allergy, Asthma & Clinical Immunology* 2023, **19**(Suppl 1):O-43

**Introduction:** The estimated frequency of angioedema as a side effect of ACE inhibitor (ACEi) treatment varies between 0.1 and 1% of treated patients. There are evidences that increased levels of bradykinin have an important role in the pathophysiology of some of these AE. These ACEi- AE are very severe and life threatening. Spontaneous mast cell angioedema (MC-AE) are like spontaneous urticaria common in the general population and can occur in patients taking an ACEi. MC-AE are not very severe and rarely endanger the vital prognosis. Spontaneous MC-AE and ACEi-AE do not have the same prognosis nor the same impact, i.e. the definitive contraindication of ACEi. Indeed, If ACE inhibitors are not withdrawn, ACEi-AE recurrences tend to increase both in frequency and in severity. Our center has been receiving patients with suspected ACEi-induced BK-AE for several years. Through our national hotline, we ask emergency physicians that any patient suspected of ACEi-AE be re-evaluated by a CREAK specialist. Indeed, a significant proportion of patients labeled as ACEi-AE in the emergency room are in fact spontaneous MC-AE that often become chronic. In this study, we propose to compare ACEi-AE with spontaneous MC- AE initially reported to ACEi and then to propose diagnostic criteria for ACEi-AE.

**Materials, methods:** It is a multicenter retrospective study from 2019 to 2022 conducted in two CREAK centers (Grenoble and Reunion Island). We included every patients addressed for suspicion of ACEi–AE with a follow-up of at least 12 months. Diagnostic of spontaneous MC-AE was excluded if AE frequency does not improved with anti-histamines long-term prophylaxis. C1-INH assays have been performed for every patient. In some cases, PLG mutations was also researched.

**Results:** 126 patients were addressed for suspicion of ACEi-AE. Of these, 99 could be included in the study. The diagnosis of ACEi-AE was retained in 49 patients and of spontaneous MC-AE in 50. One acquired C1-INH deficiency and one HAE-PLG were identified.

**ACEi-AE:** The median age of patients at the date of the consultation was 71 years old. 4% have a history of AE before ACEi start. The median time between the start of treatment and the first attack is 1095 days (3 years). The main AE localization were tongue (60.4%) and larynx (18.75%). The median duration of ACEi-AE was 27 h. 85% of ACEi-AE patients went to emergency room for an attack. 23,4% were hospitalized in intensive care unit. Three patients were intubated. One patient died. ACEi was stopped for every patients. Thee patients (6%) have an attack after ACEi disruption. It was a single attack within less than 3 months of ACEi stopping. ACE inhibitors were reintroduced in 2 patients: in both cases, the patients experienced AE again. After 3 months without ACEi, no patients relapsed with a median follow up of 12 months.

**Spontaneous MC-AE:** Patients with MC-AE have a median age of 53,5 years old. 22.5% of them have a history of AE before ACEi start. The ACEi was prescribed since 1267,5 days (3.5 years) when the first attack occurred. Tongue AE occurred in 55% of patients. The median duration of MC-AE was 18 h. ACEi was stopped for every patients and during all the follow up (median duration: 24 months). 30,6% had hives in the same period as AE and 12.24% during the follow up. 47% of patients have had many AE attacks after ACEi disruption. Every patients improved with anti-histamines long-term prophylaxis.

**Conclusion:** Based on the characteristics of these angioedema, we can propose diagnostic criteria for AE with IEC.

The main ones are: the absence of angioedema before starting an ACEi, and the absence of relapse after 3 months of stopping the ACEi.

## O-44 Autoimmune disorders in C1-INH-Hereditary Angioedema: preliminary data of a prospective study from an ITACA cohort

### Paola Triggianese, Francesco Cellupica, Stella Modica, Elisabetta Greco, Alberto Bergamini, Mauro Cancian

#### Department of System Medicine, Tor Vergata University Hospital, Rome, Italy

*Allergy, Asthma & Clinical Immunology* 2023, **19**(Suppl 1):O-44

**Background:** In Hereditary Angioedema (HAE) resulting from the deficiency of C1-inhibitor (C1-INH-HAE), the potential inadequate clearance of immune-complexes in the presence of reduced levels of complement components may result in inflammatory damage and release of autoantigens triggering autoimmune responses. Few studies on large patient populations documented controversial results on autoimmune diseases (AIDs) in patients with C1-INH-HAE. Thus, the occurrence of AIDs in C1-INH-HAE patients remains debated.

**Patients and methods:** We performed a prospective study on autoimmunity (including autoantibodies and defined AIDs) from a consecutive C1-INH-HAE Italian patients referring to the Reference Centre Tor Vergata University Hospital, in Rome (Italy). Inclusion criteria were a defined diagnosis of type I/II C1-INH-HAE, non-pediatric age at the enrollment (≥ 16 y.o.), and the consent to study, in the period Jan-Dec 2022.

**Results:** Among 190 type I/II C1-INH-HAE patients, 110 patients were included but 17 subjects drop out the study. The study cohort thus included 93 C1-INH-HAE patients (type I 94.6% and type II 5.4%, 19.4% de novo diagnosis) with F:M ratio 1.3 and a mean age at the enrollment 43.6 ± 17 yrs. Anti-thyroid antibodies were revealed in 16.7% of the cohort, anti-nuclear (ANA, ≥ 1:160 titer) in 18.5% with concomitant extractable nuclear antibodies in 6%, anti-citrullinated antibodies in 6.7%, and a single case of positivity to rheumatoid factor. A defined diagnosis of AIDs occurred in 22.6% of patients with a F:M ratio 2.0 and included connective tissue diseases and inflammatory arthritis in 52.4% and 28.6% of the cohort, respectively; the remaining 19% had autoimmune thyroiditis. In 95.2% of cases, C1-INH-HAE diagnosis was done before the AIDs detection. Interestingly, patients with AIDs were on long term prophylaxis in a significantly higher proportion than patients without AIDs (p = 0.0015).

**Conclusions:** C1-INH-HAE patients may have concomitant AIDs that appear to affect both disease outcome and treatment.

## O-45 Health-related quality of life with garadacimab for Hereditary Angioedema prophylaxis: Results from a Phase 2 trial

### Emel Aygören-Pürsün^1^, William R. Lumry^2^, Inmaculada Martinez Saguer^3^, Joshua S. Jacobs^4^, William H. Yang^5^, Paul K. Keith^6^, Paula Busse^7^, Avner Reshef^8^, Timothy J. Craig^9^, John-Philip Lawo^10^, David Kormann^11^, Ingo Pragst^10^, Markus Magerl^12^

#### ^1^Klinikum der Johann Wolfgang-Goethe Universität, Klinik für Kinder- und Jugendmedizin, Frankfurt, Germany; ^2^AARA Research Center, Dallas, TX, USA; ^3^HZRM Haemophilia Center Rhein Main, Mörfelden-Walldorf, Germany; ^4^Allergy & Asthma Clinical Research, Walnut Creek, CA, USA; ^5^Ottawa Allergy Research Corporation, Department of Medicine, University of Ottawa, Ottawa, ON, Canada; ^6^McMaster University Medical Centre Site, Hamilton, ON, Canada; ^7^Mount Sinai, New York, NY, USA; ^8^Allergy, Immunology & Angioedema Center, Barzilai University Hospital, Ashkelon, Israel; ^9^Allergy, Asthma and Immunology, Department of Medicine and Pediatrics, Penn State University, Hershey, PA, USA; ^10^CSL Behring Innovation GmbH, Marburg, Germany; ^11^CSL Behring, King of Prussia, PA, USA; ^12^Institute of Allergology, Charité—Universitätsmedizin Berlin, and Fraunhofer Institute for Translational Medicine and Pharmacology ITMP, Immunology and Allergology, Berlin, Germany

*Allergy, Asthma & Clinical Immunology* 2023, **19**(Suppl 1):O-45

**Background:** Hereditary Angioedema (HAE) carries substantial disease burden and impacts quality of life (QoL). HAE long-term prophylaxis (LTP) may improve QoL vs on-demand therapy [1]. LTP with 75 mg, 200 mg, and 600 mg once-monthly subcutaneous garadacimab elicited significantly decreased attack rates vs placebo (median reduction: 100%, 100%, and 93%, respectively; P < 0.001) in a Phase 2 study [2]. Embedded QoL outcomes in the Phase 2 placebo-controlled (TP1) [2] and open-label extension (OLE; NCT03712228) periods are reported.

**Methods:** In TP1, 32 patients were randomised to placebo (n = 8) or garadacimab 75 mg (n = 9), 200 mg (n = 8) and 600 mg garadacimab (n = 7) once-monthly (28 ± 2 days). At OLE initiation, patients received 200 mg (n = 20) or 600 mg (n = 18) garadacimab (continued dose from TP1 or newly randomised). After protocol amendment, patients receiving 600 mg underwent dose reduction to 200 mg. Validated QoL outcomes [Angioedema Quality of Life (AE-QoL) Work Productivity and Activity Impairment (WPAI)] and patient-reported outcomes [Subject’s Global Assessment of Response to Therapy (SGART)] were exploratory objectives.

**Results:** In TP1, median exposure for 75 mg, 200 mg and 600 mg was 84 days [interquartile ranges (IQR) 83‒85, 83‒86, 83‒86, respectively]. In OLE, overall median (IQR) exposure was 727 days (668–784), maximum ~ 2.2 years.

Garadacimab achieved clinically meaningful improvements (≥ 6 points) [3] from baseline in total AE-QoL scores in TP1 and throughout OLE. During TP1, mean [standard deviation (SD)] improvement in total AE-QoL score from baseline was 26.0 (8.6; n = 6), 20.0 (12.0; n = 8) and 15.6 (14.1; n = 5) points for 75, 200, or 600 mg garadacimab, respectively, vs − 0.4 (7.9; n = 8) with placebo. At OLE end, mean (SD) improvement in total AE-QoL score was 29.1 points (14.6; n = 29).

Garadacimab demonstrated reduced work productivity loss (per WPAI) from baseline in TP1 and throughout OLE. During TP1, mean (SD) improvement from baseline was 39.7% (20.4; n = 5), 34.2% (27.1; n = 5) and 35.7% (32.7; n = 3) for 75, 200 or 600 mg garadacimab, respectively, vs 2.0% (8.4; n = 5) for placebo. At OLE end, mean (SD) change from baseline was 34.2% (31.8; n = 16). Similar trends were observed across all WPAI domains.

For SGART, 100% of patients [n = 23/23] receiving 75, 200 or 600 mg garadacimab vs 12.5% [n = 1/8] receiving placebo in TP1 and 93.9% (n = 31/33) at OLE end rated their response as ‘good’ or better.

**Conclusions:** Garadacimab (for up to 2.2 years) elicited clinically meaningful and sustained improvements across QoL instruments AE-QoL and WPAI as well as SGART for patients with HAE.

**References**

1. Park K, Yeich A, Craig T. Evaluating the impact of acute versus prophylaxis therapy in Hereditary Angioedema. J Allergy Clin Immunol. 2023;151(2):AB135.
2. Craig T, Magerl M, Levy DS, et al. Prophylactic use of an anti-activated factor XII monoclonal antibody, garadacimab, for patients with C1-esterase inhibitor-deficient Hereditary Angioedema: a randomised, double-blind, placebo-controlled, phase 2 trial. Lancet. 2022; 399:945–955.
3. Weller K, Magerl M, Peveling-Oberhag A, et al. The Angioedema Quality of Life Questionnaire (AE-QoL)—assessment of sensitivity to change and minimal clinically important difference. Allergy. 2016; 71:1203–1209.

## O-46 Quality of life in Hereditary Angioedema in Brazil: A multicentric study

### Lucca Nogueira Paes Jannuzzi^1^, Gabriel Abila Gonçalves^1^, Marina Teixeira Henriques^2^, Daniel Prado dos Santos^5^, Henrique Sarquis Serpa^6^, Sabrina Macely Souza dos Santos^8^, Dhallya Andressa da Silva Cruz^8^, Isabela Cristina Amaral Dantas^9^, Luciana Costa Pinto da Silva^9^, Vânia Mesquita Gadelha Prazeres^9^, Bianca de Souza Leite Sender^10^, Têmis Félix^7^, Solange Oliveira Rodrigues Valle^3^, Faradiba Sarquis Serpa^4^, Anete Sevciovic Grumach^2^

#### ^1^Graduate student of University Center FMABC, Santo Andre, SP, Brazil; ^2^Clinical Immunology, Department of Clinical Medicine, University Center Faculty of Medicine ABC, Santo Andre, SP, Brazil; ^3^Hospital Universitário Clementino Fraga Filho, Universidade Federal do Rio de Janeiro, Rio de Janeiro, RJ, Brazil; ^4^Escola Superior de Ciências da Santa Casa de Misericórdia—EMESCAM, Vitória, ES, Brazil; ^5^Graduate student of Escola Superior de Ciências da Santa Casa de Misericórdia—EMESCAM, Vitória, ES, Brazil; ^6^Graduate student of University Center Multivix Vitória, ES, Brazil; ^7^Clinical Hospital of Porto Alegre—HCPA, Porto Alegre, RS, Brazil; ^8^Graduate student of Federal University of Amazonas, Manaus, AM, Brazil; ^9^Policlínica Codajás, Manaus, AM, Brazil; ^10^Graduate student of Instituto Federal do Rio de Janeiro, Rio de Janeiro, RJ, Brazil

*Allergy, Asthma & Clinical Immunology* 2023, **19**(Suppl 1):O-46

**Introduction:** As the medicine evolves quickly and begins to understand the patient holistically integrating biological, psychological and social factors, the value of understanding every part that composes the quality of life of these patients increases. That matter is even bigger in which concern to rare diseases as Hereditary Angioedema. Lack of information, difficult access to treatment and diagnosis delays are proporcional to the burden in patients quality of life.

With that in mind, this study aim to measure the impacts in quality of life of Hereditary Angioedema with C1-inhibitor deficiency (C1-INH-HAE) in specialized treatment centers in Brazil.

**Methods:** The present study is part of the National Network for Rare Diseases project. It has a prospective and retrospective design that aims to understand and measure quality of life of patients/caregivers with C1-INH-HAE in Brazil. It was carried out in 7 reference centers and with 116 patients which 91 of those answered. Two different questionnaires were applied for these patients/caregivers. AE-QoL, where higher scores indicate higher impairment and SF-36, where higher scores indicate better quality of life.

**Results:** Analyzing SF-36, which is divided in 8 domains (Physical functioning, physical role limitations, bodily pain, general health perceptions, energy/vitality, social functioning, emotional role limitations and mental health) the most affected domains were bodily pain (41,26), physical role limitations (42,68) and emotional role functioning (43,95) scored out of 100. General health perceptions had 55,16 out of 100. Physical functioning was the less impaired domain (73,00) The average was 52,20 out of 100. In AE-QoL, which is divided in 4 domains (food, fears/shame, fatigue/mood and functioning) the most affected domain was fears/shame (61,62 out of 100). Food was the less impaired domain (28,24 out of 100) and the average was 42,48 out 100.

**Conclusion:** Studying the results, both questionnaires are in agreement since emotional aspects at SF-36 and fears/shame at AE-QoL were the most impaired domains. Thoughts like fear of asphyxia and death or not knowing when the next attack would occur are key reasons to increase the burden of the disease. Added to that, physical questions like severe pain when there is recurrence of crisis also decreased the total average in both questionnaires.

## O-47 Real-world effectiveness data on lanadelumab in HAE long term prophylaxis in Slovakia

### Miloš Ješenak^1,2,3^, Anna Bobcakova^2,^*, Katarína Hrubiskova^4^, Veronika Urdova^2,3,5^, Otilia Petrovicova^1^, Eva Malicherova Jurkova^1^, Branislav Slenker^1^, Peter Banovcin^1^

#### ^1^National Centre for Hereditary Angioedema, Department of Paediatrics, Jessenius Faculty of Medicine in Martin, Comenius University in Bratislava, University Teaching Hospital in Martin, Slovakia; ^2^National Centre for Hereditary Angioedema, Department of Pulmonology and Phthisiology, Jessenius Faculty of Medicine in Martin, Comenius University in Bratislava, University Teaching Hospital in Martin, Slovakia; ^3^Department of Clinical Immunology and Allergology, University Teaching Hospital in Martin, Slovakia; ^4^National Centre for Hereditary Angioedema, Department of Internal Medicine, Faculty of Medicine, Comenius University in Bratislava, University Teaching Hospital in Bratislava, Slovakia; ^5^Department of Internal Medicine, F.D.Roosevelt Teaching Hospital, Banska Bystrica, Slovakia

##### *Correspondence: abobcakova@gmail.com

*Allergy, Asthma & Clinical Immunology* 2023, **19**(Suppl 1):O-47

**Background:** The main goal of HAE management is the achievement of full control over the disease activity. In the recent years, an arrival of innovative molecules was noticed in the field of long-term prophylaxis (LTP) for HAE. Among them, lanadelumab (LAN) represents the first monoclonal antibody registered for the HAE. Its efficacy and safety were confirmed by several clinical trials. However, real-world evidence (RWE) data are essential to confirm these results in the daily clinical practice.

**Patients and Methods:** Currently, 126 living patients with HAE were identified in Slovakia (prevalence 1:43 000) and are detailly monitored in the National HAE Registry. LAN was registered in Slovakia in 2021. All the patients with at least 1 HAE attack per months within the last 12 months can be indicated for this therapy. We analysed the clinical effectiveness and tolerability of LAN in the prevention of HAE attacks in a cohort of Slovak HAE patients. We assessed the changes in attacks’ frequency and their severity and the safety of this therapy.

**Results:** Till January 2023, 17 HAE patients (aged 21.24 years, range 24–66 years; 14 females, 82%) are treated with LAN (13.5% of all HAE patients). All these patients fulfilled the indication criteria for LAN. Three treated males were switched from berotralstat due to loss of its efficacy and the rest of the patients were treated with tranexamic acid, attenuated androgens or intravenous C1-INH concentrate. The mean duration of LAN treatment was 11.5 months (range 0–19 months). The average number of HAE attacks within the 12 months before the initiation of LAN was 26.06/12 months (range 16–47) and during the treatment this number decreased to 1.44 (range 0–7). In half of the patients, no attack emerged during the LAN treatment. For the next analysis, we evaluated only 6 patients with the duration of the LAN treatment longer than 12 months. The mean number of HAE attacks decreased from 29.83 (range 22–40) to 2 (range 0–5) during the 12 months before of during the treatment. We didn’t notice any systemic or moderate-to-severe local side effects. All the patients were trained for auto-application at home facilities and their concordance was excellent.

**Conclusions:** RWE data from Slovakia clearly confirm the effectiveness and good tolerability of lanadelumab in the long-term prophylaxis of HAE attacks. This molecule can help to achieve the full control over the disease, even in the most severe symptomatic patients. Lanadelumab presents the breakthrough strategy in the long-term prevention of HAE attacks.

## O-48 Understanding the impact of HAE attacks on patient reported quality-of-life—analysis of real-world patient data

### David R. Hinds^1,^*, Jennifer A. Mellor^2^, Lucy V. Earl^2^, Margaret Cho^1^, Hannah L. Connolly^2^, Kieran S. Wynne-Cattanach^2^, Charlotte L. Camp^3^, Sara Dosenovic^3^

#### ^1^BioMarin Pharmaceutical, San Rafael, CA, USA; ^2^Adelphi Real World, Bollington, UK; ^3^BioMarin Pharmaceutical, London, UK

##### *Correspondence: davidhinds@bmrn.com

*Allergy, Asthma & Clinical Immunology* 2023, **19**(Suppl 1):O-48

**Background:** Hereditary Angioedema (HAE) is characterized by unpredictable, painful, and recurring angioedema attacks. HAE affects patients’ activities of daily living and overall health-related quality-of-life (HR-QoL). Real-world research quantifying the effect of HAE attacks on HR-QoL is limited.

**Methods:** Data were drawn from the Adelphi HAE Disease Specific Programme™, a real-world point-in-time survey of physicians and HAE patients in the United States. Physicians recorded patient demographics, clinical history, current treatment, and recent attack history for their HAE patients seen between July-November 2021. These patients were invited to provide self-reported data on their attack history and HR-QoL using the Angioedema Quality-of-Life (AE-QoL) tool. In this analysis, AE-QoL results, along with patient characteristics, were described among adult patients with HAE type I or II who had self-reported data stratified based on patient report of any vs. no attacks in the previous month (recall period for AE-QoL).

**Results:** Of the 108 patients in the analysis, 25 (23.1%) reported an attack in the previous month. Demographics of patients who reported or did not report an attack in the previous month were generally similar: 52.0% vs. 45.8% female, mean age 35.2 vs. 30.0 years, mean time since HAE diagnosis 5.6 vs. 4.4 years, respectively. Those who reported an attack in the previous month also experienced more attacks in the last 12-months: mean (SD) attacks in the last 12 months 5.3 (4.7), n = 25 vs. 2.3 (1.6), n = 83. 96.0% of those reporting an attack in the previous month were on long-term prophylaxis at the time of data collection (i.e., AE-QoL completion) vs. 83.2% not reporting an attack. Patients who reported an attack in the past month had higher mean AE-QoL scores, indicating more adverse impact on HR-QoL, than those who did not report an attack, as measured by total score (mean [SD] 39.8 [16.14] vs. 24.4 [13.36]) and domain scores (functioning, 39.2 [14.27] vs. 19.1 [15.88]; fatigue/mood, 35.8 [18.62] vs. 21.0 [14.24]; fears/shame, 46.1 [20.59] vs. 33.9 [18.50]; nutrition: 35.5 [21.55] vs. 15.1 [13.49]).

**Conclusion:** HAE patients who experienced an attack in the previous month reported worse HR-QoL with the greatest impact regarding fears/shame followed by functional impacts. This suggests that anxiety and fear of experiencing an attack, along with functional impacts, are driving factors in overall HR-QoL for HAE patients on current therapies. Further research is required to understand how attack characteristics affect HR-QoL, along with incorporating these observations into the holistic impact of HAE for patients.

## P-01 One-year epidemiologic data from Angioedema Center Vienna, Austria

### Tamar Kinaciyan

#### Department of Dermatology, Medical University of Vienna, Vienna, Austria

*Allergy, Asthma & Clinical Immunology* 2023, **19**(Suppl 1):P-01

**Background:** In Austria, around 145 people are currently known to suffer from C1-esterase inhibitor deficiency-associated Hereditary Angioedema type I/II (C1-INH-HAE). Moreover, 6 other HAE-types with normal C1-INH values (nC1-INH-HAE) have been identified in recent years. Epidemiological data on these are widely missing yet. While around 450 individuals with F-XII mutation are estimated worldwide, other forms have been identified in 4–5 individuals in 1–2 families each. There is no data from Austria on this. The aim of our register is to reduce this gap for all HAE forms in Austria. Here we report one-year epidemiologic results from Vienna.

**Method:** To rise epidemiologic data, awareness activities like webinars and talks were given and articles in medical non-medical journals published, and all patients who visited the angioedema Outpatient Clinic of Department of Dermatology Vienna with isolated angioedema were subjected to diagnostic tests for HAE after a precise case history was taken.

**Results:** In 2021, 82 angioedema patients have been screened. Among them, we identified thirteen (15%) ACE inhibitor-induced, one unspecified infection-associated, one Helicobacter Pylori-related, three (3,7%) food-related, and three (3,7%) drug- related angioedema patients. Additional three (3,7%) patients suffered from autoimmune diseases, one had sialadenitis-related unilateral facial swelling, and one patient reported globus sensations due to silent reflux. Another thirty (36%) people suffered from histaminergic angioedema of unknown cause. Among the remaining twenty-six (32%) patients, we identified one patient with C1-INH-HAE type I and an 80-year-old man with decreased C1-INH levels without underlying hematologic disease. Genetic analysis had been initiated for the remaining 24 (29,3%) patients with steroid-resistant angioedema attacks and nC1-INH levels.

**Conclusion:** In summary, we diagnosed one new patient with C1-INH-HAE type I, one elderly patient with late onset C1-INH-HAE type I or acquired AE patient, four women and one man with nC1-INH-HAE. Another 19 people with suspected nC1-INH-HAE are still being investigated and are under observation, while HAE could be ruled out in 56 patients. Shire-Takeda IIR-AUT-002649.

## P-02 Outcome parameters to measure efficacy of prophylactic therapy for Hereditary Angioedema: a systematic review

### Remy S. Petersen*, Lauré M. Fijen, Danny M. Cohn

#### Department of Vascular Medicine, Amsterdam Cardiovascular Sciences, Amsterdam UMC, University of Amsterdam, Amsterdam, The Netherlands

##### *Correspondence: r.s.petersen@amsterdamumc.nl

*Allergy, Asthma & Clinical Immunology* 2023, **19**(Suppl 1):P-02

**Background:** In recent years, several new prophylactic treatment options for Hereditary Angioedema (HAE) have been developed and studied. To accurately compare these new therapies, it is pivotal to use uniform outcome measures. Fijen et al. showed there is extensive heterogeneity in outcome measures used to assess efficacy of on demand treatment for HAE [1] leading to a currently ongoing consensus project to create a core outcomes set for studies investigating on demand treatment. In this study, we assessed if a similar variety in primary and secondary efficacy outcome measures is present in randomized controlled trials investigating prophylactic treatment for HAE.

**Methods:** MEDLINE, EMBASE and Cochrane Central were systematically searched from their commencements until November 2022. Title and abstract and subsequent full report screening was performed by two researchers independently. Disagreements were resolved through discussion. Articles of randomized controlled trials examining patients with HAE due to C1-inhibitor deficiency of all ages that reported at least one outcome parameter and were published in English were included.

**Results:** From a total of 2951 publications, 25 eligible articles were included, reporting results from 22 different studies, including two open label extension (OLE) trials that randomized participants between different doses. All outcomes were extracted verbatim and translated to standardized outcome measures. 80% (16/20) of the non-OLE studies reported the standardized outcome measure *‘time-normalized angioedema attack frequency’* as their primary efficacy outcome. All studies that reported a different primary efficacy endpoint were published before 2003. Still, there were differences observed in the time period used to measure efficacy and the adjudication of angioedema attacks. 75% of the studies measured attack frequency during the complete treatment period, and 25% measured attack frequency during effective dosing period. Angioedema attacks were adjudicated by the patient themselves, the investigator, an expert adjudication panel, and an independent expert, in 43%, 31%, 19%, and 6% of the studies, respectively. There was considerable heterogeneity in secondary endpoints, as there were 43 different standardized outcome measures reported and none of these were reported in all 22 studies.

**Conclusions:** This systematic literature review shows that while there is some homogeneity in primary efficacy outcome measures in trials with prophylactic treatment in HAE, these outcome measures are still not completely comparable. Moreover, there is a wide variation in secondary outcome measures. We thus argue that a core outcome set for future trials would enhance comparison between the various prophylactic agents.

**Reference**

1. Fijen LM, Petersen RS, Cohn DM. Outcome measures in randomized controlled studies of acute therapy for Hereditary Angioedema: A systematic review. Allergy. 2022.

## P-03 Efficacy and safety of Lanadelumab in Russian participants with Hereditary Angioedema (HAE)

### Elena A. Latysheva^1,2^, Irina A. Manto^1^, Liubov V. Aleshina^3^, Elena N. Bobrikova^4^, Ekaterina A. Viktorova^5^, Elena M. Gracheva^6^, Darya V. Demina^7^, Darya S. Fomina^4,8^, Anna Yu. Shcherbina^5^, Tatiana V. Latysheva^1,9^

#### ^1^National Research Center – Institute of Immunology Federal Medical-Biological Agency of Russia, Moscow, Russian Federation; ^2^Pirogov Russian National Research Medical University, Moscow, Russian Federation; ^3^Saratov State Medical University named after V.I. Razumovsky, Saratov, Russian Federation; ^4^Clinical State Hospital No. 52, Moscow, Russian Federation; ^5^Dmitry Rogachev National Medical Research Center of Pediatric Hematology, Oncology and Immunology, Moscow, Russian Federation; ^6^Vologda Regional Clinical Hospital, Vologda, Russian Federation; ^7^Research Institute of Fundamental and Clinical Immunology, Novosibirsk, Russian Federation; ^8^I.M. Sechenov First Moscow State Medical University (Sechenov University), Moscow, Russian Federation; ^9^Moscow State University of Medicine and Dentistry named after A.I. Evdokimov, Moscow, Russian Federation

*Allergy, Asthma & Clinical Immunology* 2023, **19**(Suppl 1):P-03

**Background:** Lanadelumab—is a modern medicine developed and used to prevent attacks in patients with Hereditary Angioedema (HAE) aged 12 years and over. A retrospective study (IISR-2021-200085) was conducted in order to evaluate its efficiency and safety in real-life practice in Russia.

**Methods:** 16 patients with HAE type 1 who initiated treatment with lanadelumab (300 mg every 14 days) were enrolled. The effectiveness was evaluated by comparison of patient-reported attack rate and PROs (AAS, AECT, AE-QoL, HAE-AS—only adults) before treatment and on-treatment up to 6 months (Wilcoxon signed-rank test). The incidence of adverse events (AEs) was evaluated.

**Results:** The majority of patients were female 81% (13/16). The middle age was 29.9 years (min = 13, max = 47); 19% (3/16) were adolescents.

Prior to initiating lanadelumab, 69% (11/16) of patients received long-term prophylaxis (3/danazol, 4/C1-inhibitor IV, 4/tranexamic acid), which was cancelled after lanadelumab initiation.

The initial dosing interval after 3 months of treatment for symptoms-free patients was increase gradually (+ 7 days). At the time point of data collection 3 patients used an average fixed injection interval of 14 days, 2—of 21; 9—of 28; 1—of 30; 1—of 56.

The median number of attacks/month was 10 (min = 1, max = 17) and treated attacks/month pre-lanadelumab was 4.7 (min = 1, max = 8) per patient. After 6 months of treatment these values were 0.26 (min = 0, max = 0.83; p < 0.001) and 0,09 (min = 0, max = 0.33; p < 0.001). 10 patients were absolutely attack-free for 6 months after the treatment start. The mean HAE-AS score after 6 months of treatment decreased from 16.2 (min = 5; max = 23) to 2.3 (min = 0; max = 6); p < 0.001.

After 3 months of treatment the mean AECT values improved from 5.6 (poor control) to 14.2 (good control) (p < 0.001), and all patients showed an adequate disease control. After six months of treatment mean AECT 15.1 (3 vs 6 months p = 0.077). Total control (16 points) was observed in 7/13 adult patients after 6 months of treatment.

After 6 months of treatment AE-QoL decreased from 58 (min = 17; max = 82) to 19 (min = 1; max = 54), p < 0.001.

No serious AEs connected with lanadelumab were observed.

**Conclusion:** Our study demonstrated that lanadelumab not only minimized the attack rate in HAE patients but also improved quality of life. The extension of injection intervals in most patients do not lead to losing efficacy. Good safety of lanadelumab was observed.

Clinical trial IISR-2021-200085

## P-04 Mimics of Hereditary Angioedema in an emergency department

### Marko Barešić, Boris Karanović, Ljiljana Smiljanić Tomičević, Darija Čubelić, Branimir Anić

#### Division of Clinical Immunology and Rheumatology, Department of Internal Medicine, School of Medicine, University Hospital Center Zagreb, Zagreb, Croatia

*Allergy, Asthma & Clinical Immunology* 2023, **19**(Suppl 1):P-04

Hereditary Angioedema (HAE) is a disease with an unpredictable course and the attacks can occur at any time, with or without provocation. Most of the patients with their first severe attack end up in Emergency departments (ED) and are seen by Emergency medicine doctors or ENT specialists. The first clinical presentation of bradykinin-mediated and non-bradykinin-mediated angioedema is hard to differentiate in cases of emergency, especially if the facial or laryngeal structures are involved.

We present the clinical cases of five patients from the ED with the angioedema of the head, face and tongue.

**Patient 1:** Male (born 1954) presented with the swelling of the lips, face and tongue and afterwards developed stridor. Antihistamines and glucocorticoids proved ineffective. He had to be mechanically ventilated. He was treated with icatibant and conestat-alpha and his condition gradually improved. He was diagnosed with ACEI-induced angioedema.

**Patient 2:** Female (born 1950) presented with the swelling of her face, dysphagia and hoarseness. Antihistamines and glucocorticoids were ineffective. Symptomatic treatment reduced the swellings. She was diagnosed with ACEI-induced angioedema.

**Patient 3:** Male (born 1960) presented with a recurrent swelling of the face and dyspnea. Antihistamines and glucocorticoids had no effect. Icatibant was partially effective. The swelling regressed and he was diagnosed with ACEI-induced angioedema. During the hospitalization, he developed symptoms of progressive muscle weakness. He was diagnosed with myasthenia gravis, likely induced by the glucocorticoids used for the treatment of angioedema.

**Patient 4:** Male (born 1943) presented with the swelling of his face and tongue. Antihistamines and glucocorticoids had no effect. He was treated with conestat-alpha and his condition improved. He was diagnosed with ACEI-induced angioedema.

**Patient 5:** Female (born 1959) presented with a recurrent swelling of her neck, face and uvula. She was taking ACEI. Additional work-up was performed and a mediastinal mass was detected. Disseminated carcinoma of the mediastinum with the superior vena cava syndrome and blood stasis caused the swelling of the neck and face.

All of the described patients had normal levels and function of C1-inhibitor, excluding HAE type I and II. Four out of the five patients used ACEI for the treatment of hypertension and were diagnosed with ACEI-induced angioedema. One patient had carcinoma with superior vena cava syndrome, which mimicked angioedema.

Presenting symptoms of the patients called for a quick reaction in the ED and the initiation of treatment as their diagnosis was Hereditary Angioedema. Mimics of HAE are not rare.

All patients gave explicit permission for their information to be published.

## P-05 Mechanistic modeling and simulations predict long-term HAE attack prevention with STAR-0215

### Jou-Ku Chung^1,^*, Haobin Luo^2^, John Tolsma^2^, Pradeep Bista^1^, Andrew Nichols^1^, Chris Morabito^1^

#### ^1^Astria Therapeutics, Boston, MA, USA; ^2^RES Group, Needham, MA, USA

##### *Corresponence: jchung@astriatx.com

*Allergy, Asthma & Clinical Immunology* 2023, **19**(Suppl 1):P-05

**Background:** Inhibition of plasma kallikrein is a validated mechanism for prevention of Hereditary Angioedema (HAE) attacks. Clinically, STAR-0215 demonstrated a long circulating half-life (estimated 117 days) and prolonged plasma kallikrein inhibition (at least 84 days). Simulation of different dose regimens was performed using a mechanistic quantitative systems pharmacology (QSP) model to explore the potential for the reduction of HAE attacks.

**Materials and methods:** A simplified QSP model was established based on published reaction parameters for the plasma kallikrein-kininogen pathway in the vascular space and adjacent to the endothelial surface. The human pharmacokinetic (PK) parameters of STAR-0215 were derived from healthy adult subjects in a Phase 1a trial. The virtual cohorts were established using human PK parameters and their variabilities identified from healthy subjects who received STAR-0215. A Poisson distribution was applied to randomly determine the timing and frequency of HAE attack events with an average baseline frequency of 3 attacks per month.

**Results:** The QSP model predicted long-term robust HAE attack suppression following a single subcutaneous administration of STAR-0215 at dose levels of ≥ 300 mg. Simulations also showed subcutaneously administered STAR-0215 potentially could provide prolonged HAE attack suppression with a once every 3 months dose regimen.

**Conclusions:** Results from the QSP model simulations support STAR-0215 dosing once every 3 months for robust suppression of HAE attacks.

## P-06 The impact of dental procedures on angioedema attacks—An observational study of patients enrolled in the Romanian Hereditary Angioedema Registry

### Valentin Nădăşan^1,^*, Konrád-Ottó Kiss^2^, Réka Borka-Balás^3^, Noémi-Anna Bara^4^

#### ^1^Department of Hygiene, George Emil Palade University of Medicine, Pharmacy, Science, and Technology of Targu Mures, Targu Mures, Romania; ^2^Department of Anaesthesiology and Critical Care Medicine, Mures Emergency Clinical County Hospital, Targu Mures, Romania; ^3^Department of Paediatrics, Mures Emergency Clinical County Hospital, Targu Mures, Romania; ^4^Hereditary Angioedema Expertise Centre, Sangeorgiu de Mures, Romania

##### *Correspondence: valentin.nadasan@umfst.ro

*Allergy, Asthma & Clinical Immunology* 2023, **19**(Suppl 1):P-06

**Background:** Hereditary Angioedema (HAE) is a rare, life-threatening condition of genetic aetiology [1]. HAE related to C1-esterase inhibitor deficiency (C1-INH) is caused by mutations in the *SERPING1* gene and has an estimated prevalence of 1:50,000 in the general population [2]. Dental procedures can trigger angioedema attacks with critical consequences [3]. The study aimed to assess the frequency of dental procedure-associated angioedema attacks, the type of medications administered to prevent or treat such attacks, and whether the perceived risk of a dental procedure-related angioedema attack may discourage patients from asking and dental professionals from offering appropriate dental care.

**Materials and methods:** The observational study included all the eligible adults from the Romanian Hereditary Angioedema Registry who provided informed consent. The impact of dental procedures on angioedema attacks was measured using a structured questionnaire including 20 questions administered via telephone. Absolute and relative frequencies of the categorical variables, the mean values, and the standard deviations of the numerical variables were computed.

**Results:** The study sample included 94 patients (66% females, 34% males; 88.3% with HAE type I, and 11.7% with HAE type II or III). Patients experienced dental procedure-related symptoms suggestive of HAE both before (47.6%) and after their condition was diagnosed (51.9%). Before the HAE diagnosis, 86.2% of the patients received glucocorticoids and antihistamines for post-procedural swelling. After diagnosis, 85.3% of the patients were given Icatibant and C1-INH. Preventive medication in the case of dental interventions was consistently administered to 8% of the respondents. More than half (55.3%) of the patients reported not demanding dental interventions because of fear of Hereditary Angioedema attacks or anticipation of refusal, and 24.7% of them declared they were denied dental care by dental health professionals at least once.

**Conclusions:** Swelling related to dental procedures was common among the studied HAE patient. Unwarranted medications used before HAE diagnosis for dental post-procedural symptoms were replaced by adequate specific medications in most patients with known HAE diagnosis. Few patients benefited from short-term prophylaxis. Many patients avoided dental interventions due to fear of Hereditary Angioedema attacks and some were denied dental care by dental health professionals.

**Reference**

1. Zanichelli A, Farkas H, Bouillet L, Bara N, Germenis AE, Psarros F, et al. The Global Registry for Hereditary Angioedema due to C1-inhibitor Deficiency. Clin Rev Allergy Immunol. 2021;61(1):77-83.
2. Aygören-Pürsün E, Magerl M, Maetzel A, Maurer M. Epidemiology of Bradykinin-mediated angioedema: a systematic investigation of epidemiological studies. Orphanet J Rare Dis. 2018;13(1):73.
3. Forrest A, Milne N, Soon A. Hereditary Angioedema: death after a dental extraction. Aust Dent J. 2017 Mar;62(1):107-110.

## P-07 The analysis of the effect of the COVID-19 pandemic on C1-inhibitor deficient Hereditary Angioedema patients

### Dávid Szilágyi, Hanga Réka Horváth, Noémi Andrási, Zsuzsanna Balla, Henriette Farkas

#### Semmelweis University, Budapest, Hungary

*Allergy, Asthma & Clinical Immunology* 2023, **19**(Suppl 1):P-07

**Introduction:** Due to the similarity between the pathomechanism of SARS-CoV-2 infections and Hereditary Angioedema due to C1-inhibitor deficiency (C1-INH-HAE), a possibility emerged that C1-INH-HAE may worsen the course of the infection, or that the infection may influence the severity of angioedema (HAE) attacks in C1-INH-HAE patients.

**Objective:** To evaluate the effects of the COVID-19 pandemic on the quality of life (QoL) of Hungarian C1-INH-HAE patients, and to survey the acute course of the infection, post COVID symptoms (PCS), vaccination coverage and the side effects of vaccines in this patient population.

**Methods:** 93 patients completed our questionnaire between 1st July 2021 and 31st October 2021. In this same period and between March 2019 and March 2020, 63 patients completed the angioedema quality of life questionnaire (AE-QoL).

**Results:** Out of those patients infected with SARS-CoV-2 in the examined period (18/93 patients; 19%), 5% required hospitalization, 28% experienced HAE attacks in the acute phase of the infection, and 44% experienced PCS. Serious vaccine reactions did not occur in any case, 4 patients out of 73 (5%) experienced HAE attacks. No significant difference (p = 0.5903) was found in the median of the AE-QoL total score, or in the number of HAE attacks prior and during the pandemic.

**Conclusions:** Based on our study, HAE patients did not experience more serious SARS-CoV-2 infection, and it did not aggravate the course of HAE either. Changes in the QoL were not significant, and vaccines were safe in HAE patients.

## P-08 Clinical spectrum of high titre anti-Ro/SSA antibodies in patients with Hereditary Angioedema with C1-inhibitor deficiency

### Sladjana Andrejević, Radovan Mijanović, Branka Bonači-Nikolić

#### Clinic of Allergy and Immunology, University Clinical Center of Serbia, Belgrade, Serbia

*Allergy, Asthma & Clinical Immunology* 2023, **19**(Suppl 1):P-08

**Background:** Anti-Ro/SSA antibodies have been used as a diagnostic marker for Sjogren's syndrome and systemic lupus erythematosus (SLE) for decades and they are also associated with a variety of other diseases such as: dermatological and hematological disorders, interstitial pneumonitis and with hereditary C2 or C4 or C1q deficiency. Meanwhile, C1-INH deficiency may predispose patients to defective clearance of apoptotic cells that provides a source of autoantigens that can result in production of autoantibodies.

**Methods:** Serbian database included 94 patients (45.7% female) with C1-INH-HAE from 48 unrelated families. The majority of patients (92.5%) had C1-INH-HAE type I. Determination of antibodies against Ro/SSA was performed for 27 patients with C1-INH-HAE. We reviewed the medical records, laboratory findings and perform physical examination of patients positive for anti-Ro/SSA antibodies for manifestations of autoimmune diseases.

**Results:** High concentrations of anti-Ro/SSA antibodies were detected in four (3 females, 1 male) patients. Three female patients were affected by type I HAE, while a male was affected by type II HAE. For all patients a disease causing mutations was found in *SERPING1* gene.

**Case Reports:**

**Patient 1** is 61-year old women with a history of recurrent swelling since the age of 2. At the age of 12 she was diagnosed with SLE and treated with prednisolone and azatioprine. A year later, SLE was in remission, but she continued to experience frequent HAE attacks. Immunosuppressive therapy was discontinued. Despite high concentration of anti-Ro/SSA antibodies, symptoms attributable to SLE are absent for almost 50 years. **Patient 2** is a 38-year woman who experienced HAE attacks since puberty. At the age of 19 the diagnosis of SLE was established and therapy with antimalarials and prednisone was initiated. **Patient 3**, age 38 was diagnosed with seropositive rheumatoid arthritis at the age of 20 and initially treated with prednisolone and metotrexate. The first presentation of HAE was laryngeal angioedema at the age of 24. She is currently treated with tocilizumab. **Patient 4**, age 71 male had a first HAE attack at the age of 8. At the age of 33 he was diagnosed with HAE type II, and since then used danazol for long-term prophylaxis. Two years ago he was diagnosed with interstitial pneumonia and therapy with pirfenidone was initiated.

**Conclusions:** Based on our findings, the prevalence (14.8%) of antibody positivity to Ro/SSA in patients with C1-INH-HAE is not negligible. All patients described above were symptomatic for HAE before developing autoimmune disease.

All patients gave explicit permission for their information to be published.

## P-09 Efficacy and safety of rituximab in angioedema with acquired C1-inhibitor deficiency

### Galith Kalmi^1,6^, Yann Nguyen^2^, Federica Defendi^3,6^, Isabelle Boccon Gibod^4,6^, Laurence Bouillet^4,6^, Aurélie Du Thanh^5,6^, Delphine Gobert^1,6^, Olivier Fain^1,6^

#### ^1^Internal Medicine Department, Saint Antoine University Hospital, Assistance Publique-Hôpitaux de Paris, Sorbonne Université, DHU i2B, Paris, France; ^2^Cochin Hospital, Internal medicine department, Assistance Publique-Hôpitaux de Paris, Paris, France; ^3^Immunology Laboratory, University Hospital, Grenoble, France; ^4^Internal Medicine Department, University Hospital, Grenoble, France; ^5^Dermatology Departement, Montpellier University Hospital, Montpellier, France; ^6^CREAK Centre de reference et d’Etude des Angioedemes à Kinine, France

*Allergy, Asthma & Clinical Immunology* 2023, **19**(Suppl 1):P-09

**Introduction:** Angioedema (AE) due to acquired C1-inhibitor (C1-INH) deficiency (AAE) are related to excessive consumption of C1-INH or to anti-C1-INH antibodies (Ab), and are associated with lymphoproliferative syndromes or monoclonal gammopathies. Standard of care for prophylactic treatment in this condition is not established. Rituximab may be effective to prevent attacks, but data are scarce.

**Material and methods:** A retrospective multicenter study was carried out in France, including all patients with AE due to acquired C1-INH deficiency and treated with rituximab between April 2005 and July 2019.

**Results:** Fifty five AAE patients were included in the study, 23 of them had an anti-C1-INH Ab. A lymphoid malignancy was identified in 39 patients; a monoclonal gammopathy in 9. There was no associated condition in 7 patients. Thirty patients received rituximab alone (N = 5) or in association with chemotherapy (N = 25). Among 51 patients with available follow-up, 34 patients were in clinical remission and 17 patients had active AE after a median follow-up of 3.9 years (IQR 1.5–7.7). Three patients died. The presence of anti C1-INH Ab was associated with a lower risk of remission (HR 0.29 (0.12–0.67), p = 0.004). Relapse was less frequent in patients with lymphoma (risk ratio 0.27 (0.09–0.80) p = 0.019) and in patients treated with rituximab and chemotherapy (risk ratio 0.31 (0.12 -0.79), p = 0.014).

**Conclusion:** Rituximab is an efficient and well-tolerated therapeutic option in AE, especially in lymphoid malignancies and in the absence of detectable anti C1-INH Ab.

## P-10 Hereditary Angioedema in the Republic of Panama

### Olga M. Barrera^1^, Marisela Williams^2^, Georgina Díaz^3^, Dayanara Herrera^4^, Janina Vergara^5^, Diva Almillategui^5^, Manuel Cruz^6^, Manuel Espinoza^7^, Indira Santos^8^

#### ^1^San Fernando Hospital, Panama; ^2^Pediatric Specialties Hospital, Social Security, Panama; ^3^Allergic Private Clinical, Santiago, Veraguas, Panama; ^4^Pediatric Specialties Hospital, Social Security, Panama; ^5^Children´s Hospital of Panama, Panama; ^6^Dr. Chicho Fabrega Hospital, Social Security, Santiago, Veraguas, Panama; ^7^Chiriqui Hospital, David, Panama; ^8^Clinical Private Practice, Panama

*Allergy, Asthma & Clinical Immunology* 2023, **19**(Suppl 1):P-10

**Background:** HAE is a rare genetic condition characterized by recurrent episodes of edema that can be found in any part of the body, this condition can be deadly.

HAE has an estimated prevalence of 1:50,000 in the global population, and is not very well know by doctors in our country.

**Objective:** To characterize the patients that have been confirmed in this country until 2022 with HAE.

**Material and methods:** The retrospective information was obtained from clinical records of the patients attended by the authors.

**Results:** We have studies 22 patients with a diagnostic of Hereditary Angioedema; nine men and 13 females, aged between 7 and 74 years old. Nineteen patients were type 1, and of which 12 were woman and 7 men. Three patients from the same family were reported as type 2. Only one patient was registered with Hereditary Angioedema with normal C1-INH. Seven patients have no family history, but the rest do. Ten patients were consanguineous with their spouse.

Our patients presented a total of 684 attacks in 2022, the majority being moderates, followed by mild crisis and severe attacks. Three patients required tracheostomy and stay in intensive care. Most of the attacks were in the following locations: subcutaneous, abdominal, and genital.

The age of onset of symptoms was highly variable, but most were between 10 and 15 years old. Prodromes were reported in 20% of these patients studied and were related to irritability, fatigue, headaches, and some with serpiginous erythema.

Associated comorbidity were Allergic Rhinitis, Bronchial Asthma, Cholecystitis, Sickle Cell Anaemia, Arterial Hypertension and Diabetes Mellitus.

**Conclusions:** The Republic of Panama, located in Central America has a data of 22 patients with HAE until 2022. According to the global prevalence, we should have 84 patients with this rare disease. We need more disclosure of it at the level of medical schools and doctors in general, to have a real data de HAE.

## P-11 Vascular neural control blockade in a COVID-19 positive Hereditary Angioedema patient

### Beatrice De Maria, Giuseppina Cassetti, Lorenza Chiara Zingale, Azzurra Cesoni Marcelli, Lorena Grano De Oro, Laura Adelaide Dalla Vecchia, Francesca Perego*

#### Istituti Clinici Scientifici Maugeri IRCCS, Milano-Camaldoli, Milan, Italy

##### *Correspondence: francesca.perego@icsmaugeri.it

*Allergy, Asthma & Clinical Immunology* 2023, **19**(Suppl 1):P-11

**Background and aim:** Hereditary Angioedema (HAE) due to C1-inhibitor deficiency, is a rare condition, characterised by recurrent swelling mediated by uncontrolled bradikinin release and sometimes precipitated by infection. Activated products of the kallikrein-kinin system might also play a role in the inflammatory processes in COVID-19. The common final pathway in HAE patients with COVID-19 is a plausible additional alteration of vascular permeability. The autonomic nervous system is one of the modulators of the vascular tone that could be investigated by means of the analysis of the Systolic Arterial Pressure (SAP) variability thus providing indices of sympathetic branch of vascular neural control.

**Case description**: a 58 y-old female HAE patient underwent an head-up-tilt-test the day before the onset of fever due to COVID-19 infection (PRE) and one month after the negative nasopharyngeal swab (POST). The patient was on long-term prophylaxis for HAE.

**Method**: continuous electrocardiogram (ECG) and non-invasive arterial blood pressure were recorded for 10 min in supine position (REST) and for 10 min during passive orthostatism at 70° (TILT). From ECG, the temporal distance between two consecutive R peaks time series (RR) was derived. From the AP signal, the SAP time series was derived, SAP being the maximum of the AP inside each RR. Baro-Reflex Sensitivity (BRS) in the low frequency band (LF) was calculated by the spectral method.

Mean (µ_RR_ and µ_SAP_) and variance (σ^2^_RR_ and σ^2^_SAP_) of the RR and SAP series were calculated. Parametric power spectral analysis of SAP time series provides indices of the cardiac and of the vascular sympathetic modulation (LF_SAP_).

**Results**: µ_SAP_ from REST to TILT decreased of 10 mmHg during PRE while remained stable in POST. The physiological increase of LF_SAP_ with TILT was absent in PRE (from 1.14 to 0.58 mmHg^2^) while it was restored in POST (from 4.17 to 5.44 mmHg^2^). µ_RR_ decreased from REST to TILT by 84 ms in PRE and by 127 ms in POST. σ^2^_RR_ at REST was lower at PRE compared to POST (436 vs 719 ms^2^). The expected decrease of BRS with TILT was absent in PRE (from 5.26 to 14.52 ms mmHg^−1^) while restored in POST (from 2.16 to 1.20 ms mmHg^−1^). During the one-month observation period no attacks occurred.

**Conclusion**: in the prodromal phase of Covid-19, this HAE patient’s autonomic profile was characterized by the absence of activation of the vascular sympathetic branch and a baroreflex dysfunction not associated with the occurrence of acute angioedema attacks.

## P-12 A value-based and human centred innovative approach based on persona: psychosocial needs of Hereditary Angioedema patients and caregivers from the ITACA cohort

### Alessandra Gorini^1,2^, Lorenza Chiara Zingale^1^, Beatrice De Maria^1^, Lorenzo Rimoldi^3^, Nurgul Nsanbayeva^3^, Giorgia Lano^3^, Bhavya Krishnan^3^, Enrico Rimoldi^3^, Maria Rosaria Natale^3^, Laura Adelaide Dalla Vecchia^1^, Francesca Perego^1,^*

#### ^1^Istituti Clinici Scientifici Maugeri IRCCS, Milan, Italy; ^2^Dipartimento di Scienze Cliniche e di Comunità, Università degli Studi di Milano, Italy; ^3^Your Business Partner, London, United Kingdom

##### *Correspondence: francesca.perego@icsmaugeri.it

*Allergy, Asthma & Clinical Immunology* 2023, **19**(Suppl 1):P-12

**Background:** Hereditary Angioedema (HAE) due to C1-inhibitor deficiency (HAE-C1-INH), is a rare, potentially life-threatening condition, clinically characterised by recurrent swelling of the extremities, genitals, bowel mucosa, face and upper airway.

Throughout their lives, HAE patients and their caregivers face numerous disease-specific challenges, such as prolonged and complex diagnostic processes, often resulting in a diagnostic delay of many years. The unpredictability of attacks and the impact of traumatic episodes on the patients’ physical and mental health limit their ability to perform daily tasks. HAE patients and their families have vast unmet physical, psychological and social needs that are often overlooked.

Value-Based Healthcare (VBH) is a model where achieving high value for patients becomes the overarching goal of healthcare delivery. Value should always be defined around the user’s needs, to develop personalised solutions. A common human-centred approach, to describing the needs of different user types, is to utilise personas. Persona is a data-driven narrative tool, based on qualitative and quantitative findings, for communicating user archetypes that capture their attitudes, goals, and behaviours. Used in design and human interaction for health technology research, persona could be extended to studies of the psychosocial and care experience.

**Aim:** to better understand unmet and unarticulated needs of HAE patients and caregivers, relating to their psychosocial aspects and care experience using personas.

**Methodology:** semi-structured qualitative interviews were conducted through 1 h online anthropological conversations with patients, patient&caregivers (double role of patient and caregiver), and healthy caregivers from the ITACA (Italian network for Hereditary and Acquired Angioedema) cohort. The conversations were analysed using MAXQDA 2022 software. Qualitative and quantitative insights from analyses, formed the basis to create personas. Written informed consents were acquired.

**Results:** we enrolled 17 subjects: 9 patients, 6 patient-caregivers, and 2 non-affected caregivers. From MAXQDA code mapping, the principal needs that emerged are the provision of psychological support, increasing awareness and better communication of HAE.

Six personas were identified describing the personal history, experience with the centre, daily management, and needs substantiated by the real quotes.

Four patient, one patient-caregiver, and one non-affected caregiver personas were identified.

Across patient personas, the most expressed needs are psychological support and better awareness amongst healthcare professionals. Caregivers want better information about the condition, including the latest therapies, and higher awareness within the community.

**Conclusions:** using persona as a narrative tool and following an innovative VBH human-centred approach may allow care providers to make better-informed decisions for enhancing the care experience.

## P-13 Decreased adhesion to the endothelium leads to elevated neutrophil granulocyte count in Hereditary Angioedema patients

### Erika Kajdácsi^1^, Zsuzsanna Balla^2^, Zsófia Pólai^1,2^, László Cervenak^1^, Henriette Farkas^1,2^

#### ^1^Research Laboratory, Department of Internal Medicine and Haematology, Semmelweis University, Budapest, Hungary; ^2^Hungarian Angioedema Center of Reference and Excellence, Department of Internal Medicine and Haematology, Semmelweis University, Budapest, Hungary

*Allergy, Asthma & Clinical Immunology* 2023, **19**(Suppl 1):P-13

**Introduction:** As many aspects of Hereditary Angioedema (HAE) due to C1-inhibitor (C1-INH) deficiency cannot be explained with the elevated bradykinin level alone, it has recently become clear that other factors also play an important role in the pathogenesis. One of these factors could be the elevated neutrophil granulocyte (NG) count, which is associated with increased NG activation in C1-INH-HAE patients; however, their origin has not been elucidated so far. Here, we aimed to investigate whether the excess of NGs results from a disturbed maturation, biased circulating/marginated pool equilibrium or decreased elimination.

**Methods:** We enrolled 20 attack-free C1-INH-HAE patients together with 21 healthy controls, and collected blood samples. We compared cell surface maturation markers, adhesion molecules, cytokine receptors, and Ca-mobilization of NG by flow cytometry, activation markers by ELISA and NG/endothelial cell adhesion by automated pipetting system.

**Results:** Cell surface markers showed normal maturation of NGs in C1-INH-HAE patients. Adhesion of NGs to endothelial cells pretreated with lipopolysaccharide or phorbol 12-myristate 13-acetate (PMA) was significantly weaker in samples from C1-INH-HAE patients, and bradykinin had no effect on the adhesion. NGs from C1-INH-HAE patients were in a more activated state when assessed by soluble activation markers without any stimulation.

**Discussion:** Our data support that the maturation of NGs in C1-INH-HAE patients is normal, whereas adhesion properties are weakly disturbed, indicating a bias between the pool of circulating and marginated NGs. Bradykinin may not be responsible for the disturbed adhesion characteristics.

## P-14 Psychodynamic factors impacting the degree of trust in the relationship between a doctor and a HAE patient

### Ekaterina Shutkova*

#### Department of Clinical Psychology, Dmitry Rogachev National Medical Research Center Of Pediatric Hematology, Oncology and Immunology, Moscow, Russian Federation

##### *Correspondence: eshutkova@gmail.com

*Allergy, Asthma & Clinical Immunology* 2023, **19**(Suppl 1):P-14 

The HAE diagnosis and a recurrent edema can cause a certain psychological burden. The more severe the disease, the more significant psychological burden it acquires, such as

1. Experience of death anxiety (in case of a laryngeal edema).
2. Sense of lacking the control over one’s own life.
3. Sense of dependence on doctors and medications.

All of the points above are the factors which provoke psychological regression of the patients.

HAE is a rare disease; the correct diagnosis can take many years, because the awareness of it, including among general physicians, is often lacking. Many patients, before they get a correct diagnosis, suffer from a misdiagnosis and mistreatment which either has no effect or even has a negative effect on their health (as in case of a surgery on the abdominal edema or the prescription of various diets and antihistamines).

This leads to high dependance on and expectations of doctors and medications, while at the same time many patients have frustrating experiences with healthcare staff and treatment.

The patient’s dependance on the doctor (as well as on healthcare officials responsible for the provision of necessary and often expensive medications) creates a breeding ground for the development of a regressive relationship on the part of the patient, the patient perceives a doctor as a parental figure, and himself as a child who needs care and support. That, in turn, results in the developing transference of the patient’s internal parent–child relationship representations towards the doctor (as well as other staff involved in the treatment). Both negative and positive experiences gained by the patient in the childhood as part the child-parent relationship are involved in the transference, impacting the degree of trust and the establishment of a working alliance between the patient and the healthcare staff. Psychological working through the child-parent relationship representations involved in the transference to the healthcare staff helps to remove the distorted perception of the current experience of the interaction with the healthcare staff and establish a more constructive interaction between the patient and the staff.

## P-15 Targeted sequencing panel for the diagnosis of Hereditary Angioedema due to C1-inhibitor deficiency

### Matija Rijavec^1,2^, Jerneja Debeljak^1^, Nina Rupar^1^, Julij Šelb^1,3^, Peter Korošec^1,4^

#### ^1^University Clinic of Respiratory and Allergic Diseases Golnik, Golnik, Slovenia; ^2^Biotechnical Faculty, University of Ljubljana, Ljubljana, Slovenia; ^3^Faculty of Medicine, University of Ljubljana, Ljubljana, Slovenia; ^4^Faculty of Pharmacy, University of Ljubljana, Ljubljana, Slovenia

*Allergy, Asthma & Clinical Immunology* 2023, **19**(Suppl 1):P-15

Hereditary Angioedema due to C1-inhibitor deficiency (C1-INH-HAE) is caused by pathogenic variants in the *SERPING1* gene, located on chromosome 11q12-q13.1. More than 800 different disease-causing pathogenic/likely pathogenic variants in *SERPING1* have been described to date. Genetic testing in C1-INH-HAE should encompass all exonic, 5’- and 3’- untranslated regions (UTRs) and exon–intron boundaries for single nucleotide variants (SNV), small indels and copy number variations (CNV, large defects) of the *SERPING1* gene. It is mainly performed with the Sanger sequencing followed by Multiplex Ligation-dependent Probe Amplification (MLPA), which is not only time-consuming but also does not allow us to detect deep intronic variants. Next-generation sequencing (NGS) has proved to be a valid replacement for conventional techniques. The present study aimed to develop and introduce into routine clinical practice a new single-step NGS method for genetic analysis of patients suspicious of C1-INH-HAE.

The QIAseq Targeted DNA Custom Panel was designed to cover the entire *SERPING1* gene, sequencing was performed on Illumina MiSeq, and the bioinformatic analysis implemented (standard GATK germline variant calling pipeline and on top of that Atlas-CNV or delly tool) allowed us concurrent detection of SNVs, small indels, and CNVs, including breakpoint predictions. Altogether, 35 different *SERPING1* variants which were previously tested by Sanger sequencing and/or MLPA, and 42 controls were included in the validation process. Furthermore, after the implementation of the method in the clinical setting, additional individuals suspicious of C1-INH-HAE were tested.

Our targeted sequencing panel provide 100% coverage of all exonic, 5’- and 3’- untranslated regions and exon–intron boundaries, as well as most of the intronic regions, with the overall coverage of the entire *SERPING1* gene being 94%. The sensitivity and specificity of rare variant detection was 100% as all identified variants were concordant with variants identified by Sanger sequencing (24 substitutions, 6 indels) and MLPA (5) and no rare variants were found in controls. Moreover, in samples with large defects, we were able to predict the precise size and location of the deleted and inserted DNA fragment in the *SERPING1* gene. Furthermore, in all additional individuals suspicious of C1-INH-HAE tested, a causative variant in *SERPING1* was identified and confirmed by Sanger sequencing.

Targeted NGS panel allows for the accurate identification of SNVs, small indels or CNVs in a rapid and straightforward manner and represents a valid single-step improvement with several advantages over conventional techniques for the genetic testing of C1-INH-HAE.

## P-16 Route of administration preferences of people with Hereditary Angioedema for on-demand treatment: A US-based qualitative study

### Vibha Desai^1,^*, Ledia Goga^1^, Shawn Czado^2^, Michelle Brown^3^, Kelley Myers^3^, Don Bukstein^4^, Paul Audhya^1^, Laurence Bouillet^5^

#### ^1^Global Medical Affairs/Outcomes Research, KalVista Pharmaceuticals, Inc., Cambridge, MA, USA; ^2^Global Market Access, KalVista Pharmaceuticals, Inc., Cambridge, MA, USA; ^3^RTI International, Inc., NC, USA; ^4^The PBL Institute, Madison, WI, USA; ^5^Internal Medicine, Grenoble Alpes University, National Reference Center for Angioedema, Grenoble, France

##### *Correspondence: vcad@kalvista.com

*Allergy, Asthma & Clinical Immunology* 2023, **19**(Suppl 1):P-16

**Background:** Hereditary Angioedema (HAE) is a rare genetic disorder characterized by unpredictable and often debilitating swelling attacks [1]. All currently approved HAE on-demand treatments must be administered parenterally, which results in significant treatment burden. The objectives of this qualitative study were to understand patients’ treated attack experiences and route of administration (ROA) preferences for on-demand treatment.

**Materials and methods:** We interviewed adolescents and adults with type 1/type 2 HAE, recruited through the US HAE Association, who had ≥ 1 HAE attack in the past 6 months and were presently utilizing on-demand treatment with or without long-term prophylaxis (LTP), to characterize their most recent treated attack experiences and assess ROA preferences. To understand trade-offs that participants were willing to make when choosing the ROA, hypothetical self-administered injection and oral on-demand treatments were initially presented with similar efficacy and safety profiles, that were then made better/worse depending upon participants’ initial treatment choice. Profiles were based on-demand injection treatments’ US package inserts and clinical trial data for oral on-demand treatment in development.

**Results:** Ten adolescents (mean age 15.5 years, 50% female, 80% on LTP) and 10 adults (mean age 36.7 years, 60% female, 50% on LTP) were interviewed. Adolescents primarily used IV pdC1-INH/rhC1-INH (60%) for their most recent attack and adults used icatibant (80%). Participants’ most favorable attribute of their on-demand treatment was effectiveness (adolescents: 50%, adults: 80%), while the least favorable attributes, were pain/burning (adolescents: 40%, adults: 30%), delayed response (adolescents: 20%, adults: 30%), and burden of administration (adolescents: 20%, adults: 30%). When presented with hypothetical on-demand treatments with similar efficacy and safety profiles, 100% of participants chose oral over injection treatment. When profiles were varied, most participants (adolescents: 90%, adults: 80%) preferred injection treatment only in a hypothetical scenario where much better efficacy versus oral treatment was demonstrated within the same timeframe. Most adolescents (80%) and adults (70%) changed their preference from oral to injection treatment, only when tolerability/mild side-effects risk was ≥ 50% with oral treatment.

**Conclusion:** All participants preferred oral over self-administered injection on-demand treatment when efficacy and safety profiles were similar. Most participants (adolescents and adults alike) preferred injection treatment only if it offered much better efficacy, and most changed their preference from oral to injection treatment, only if the oral treatment had substantively worse tolerability and/or safety profile than observed in available clinical studies. Quantitative analyses in a larger cohort are warranted to better refine on-demand treatment preferences.

**Reference**

1. Bork K, Anderson JT, Caballero T, Craig T, Johnston DT, Li HH, et al. Assessment and management of disease burden and quality of life in patients with Hereditary Angioedema: a consensus report. Allergy Asthma Clin Immunol. 2021; 17: 40

## P-17 Second patient with kininogen 1 mutation in Hereditary Angioedema

### Gaëlle Hardy^1,^*, Morgan Lamorinière^1^, Federica Defendi^2^, Nicolas Ozanne^3^, Guillaume Armengol^3^

#### ^1^Molecular Genetics Laboratory, Grenoble Alpes University Hospital, Grenoble, France; ^2^Immunology Laboratory, Grenoble Alpes University Hospital, Grenoble, France; ^3^Department of Internal Medicine, CHU Rouen, Rouen, France

##### *Correspondence: ghardy@chu-grenoble.fr

*Allergy, Asthma & Clinical Immunology* 2023, **19**(Suppl 1):P-17

Hereditary Angioedema (HAE) cosegregating with a *KNG1* mutation was fist described in 2019 in a single large family by Bork et al. [1]. No other patient was identified so far in this novel type of HAE named HAE-KNG1. We report here the molecular diagnosis of a supplementary patient with the same heterozygous probably pathogenic variant on *KNG1* (NM_001102416.3): c.1136 T > A p.(Met379Lys).

The proband is a 50 years old woman with a history of recurrent facial swelling, including lips and tongue, usually last 3 to 4 days. Three other members of her family (half-brother and his son, half-sister) presented also lips swelling, in agreement with a dominant inheritance pattern. Antihistaminic drugs and steroids had no efficacy on her symptoms. ER administration of Icatibant permitted rapid favourable outcome in a severe crisis with tongue swelling and breathe difficulty. Biological investigations showed normal C1-inhibitor activity. *F12* and *PLG* specific HAE variants were not present. *KNG1* heterozygous variant previously described was identified on two separate blood samples. This variant (class 4 ACMG) changes the N-terminal cleavage site of bradykinin from both high molecular weight kininogen (HMWK) and low molecular weight kininogen (LMWK) isoproteins. The patient's kininogen immunoblot shows a majority of uncleaved kininogen, with a molecular weight of 120 KDa, and a minority of cleaved kininogen at 45 KDa. Interestingly, her *KNG1* haplotype was different from the previously described family’s. It is therefore probably an independent molecular event, which reinforces the importance of this functional position for bradykinin synthesis from HMWK and LMWK proteins.

Despite its low frequency among HAE patients, *KNG1* exon 10 variant has to be investigated in patients with normal C1-inhibitor activity and a family history of HAE.

All patients gave explicit permission for their information to be published.

**Reference**

1. Bork K, Wulff K, Rossmann H, Steinmüller‐Magin L, Brænne I, Witzke G, Hardt J. Hereditary Angioedema cosegregating with a novel kininogen 1 gene mutation changing the N‐terminal cleavage site of bradykinin. 2019 Dec;74(12):2479-2481.

## P-18 A retrospective study (INTEGRATED) of real-world effectiveness of Lanadelumab in European patients with HAE type I/II

### Markus Magerl^1^, Laurence Bouillet^2^, Inmaculada Martinez Saguer^3^, François Gavini^4^, Laura Sayegh^5,^*, Nawal Bent-Ennakhil^4^, Irmgard Andresen^4^

#### ^1^Dermatological Allergology, Allergie-Centrum-Charité, Department of Dermatology and Allergy, Charité—Universitätsmedizin Berlin, Germany; ^2^Department of Internal Medicine, National Reference Centre for Angioedema (CREAK), Université Grenoble Alpes, Grenoble, France; ^3^Hemophilia Centre Rhine Main (HZRM), Moerfelden-Walldorf, Hessen, Germany; ^4^EUCAN Medical Affairs, Takeda Pharmaceuticals International AG, Glattpark-Opfikon, Zürich, Switzerland; ^5^Real-World Evidence, PPD, part of Thermo Fisher Scientific, Montreal, QC, Canada

##### *Correspondence: laura.sayegh@evidera.com

*Allergy, Asthma & Clinical Immunology* 2023, **19**(Suppl 1):P-18

**Rationale:** Lanadelumab, a fully human monoclonal antibody and inhibitor of plasma kallikrein, is indicated for long-term prophylaxis (LTP) of type I and II HAE with C1-INH deficiency in patients ≥ 12 years. In Europe a starting dose of 300 mg every 2 weeks (Q2W) is recommended. If the patient remains attack free with the Q2W dose, the physician can reduce the frequency to once every 4 weeks (Q4W). The study aimed to assess the effectiveness of lanadelumab (attack free rate [AFR]) and dose adjustment in preventing HAE attacks in a real-world setting.

**Methods:** This retrospective cohort chart review study was conducted in Europe (Germany, France, Austria, Greece), in patients ≥ 12 years old with HAE type I or II who initiated LTP with lanadelumab from 14 November 2017—31 October 2021. Data were collected from lanadelumab initiation (index event) to earliest of discontinuation, death, loss to follow-up, or chart abstraction initiation. Patient and treatment history were collected 12 months pre-index. Attacks were documented in the medical chart and/or diary.

**Results:** Of the 198 patients included, mean (SD) age was 43.4 (15.7) years, 61.6% were female, 91.9% had type I HAE and 30.8% had history of life-threatening attacks. In the 12 months before starting lanadelumab (pre-index), 59.6% of patients received LTP, 99.0% used on-demand treatment and 7.5% experienced ≥ 1 life-threatening attack. Most common reason for initiating lanadelumab was lack of/incomplete response to prior HAE prophylactic therapy (41.4%). Median lanadelumab treatment duration was 28.8 months (IQR: 20.4–35.7); 96% of patients were still treated at the end of data collection. Increases in intervals of administration from Q2W to any titration occurred in 72.7% of patients. On average, the first increase occurred 8.1 (SD: 6.3) months after index (range 0.54 to 33.64 months). HAE attacks were experienced by all patient’s pre-index and 60.6% patient’s post-index. Mean (SD) number of attacks 12-months pre-index was 35.8 (33.2) and 1.6 (2.9) in the first 12-months post-index. Monthly AFRs varied from 16.2% to 28.3% pre-index, and ranged from 82.7% and 84.8% (months 1 and 2) to > 95% (months 26, 36, 38, 39, and 41 +) post-index. On-demand use decreased to 52.0% of patients post-index; life-threatening attacks occurred in 1.0% of patients post-index.

**Conclusions:** The findings suggest that lanadelumab LTP improves attack free rate in HAE patients and provides real-world insight into lanadelumab utilization patterns, specifically that physicians are individualizing treatment by increasing the intervals of administration in clinical practice.

The study is sponsored by Takeda Pharmaceuticals International AG. Medical writing support for the development of this abstract was provided by PPD and funded by Takeda Pharmaceuticals International AG. The authors retain full control of the abstract content.

**Disclosures**

MM has received research grant support and/or speaker/consultancy fees from BioCryst, CSL Behring, KalVista, Pharming, and Shire (a Takeda company). LB has received honoraria and travel grants from CSL Behring, Novartis, Pharming, and Takeda, and his institute has received research funding from CSL Behring, Novartis, and Takeda. IMS has received honoraria, research funding, consultancy fees, and travel grants from and/or has participated in advisory boards for BioCryst, CSL Behring, Pharming, and Shire (a Takeda company). FG, IA and NBE are employees of Takeda and own Takeda stocks. LS is an employee of PPD, part of Thermo Fisher Scientific, Canada.

## P-19 Quality of life in patients with Hereditary Angioedema in Latvia

### Adine Kanepa^1^, Natalja Kurjane^1,2,^*

#### ^1^Riga Stradiņš University, Riga, Latvia; ^2^Pauls Stradiņš Clinical University Hospital, Riga, Latvia

##### *Correspondence: natalja.kurjane@rsu.lv

*Allergy, Asthma & Clinical Immunology* 2023, **19**(Suppl 1):P-19

**Background:** Hereditary Angioedema (HAE) is a potentially life-threatening disease that can significantly affect patient’s health-related quality of life.

**Objective: **This study aims to assess the quality of life (QoL) in patients with HAE in Latvia identifying the main reasons that reduce it the most.

**Methods: **QoL was measured using a Quality of Life Questionnaire for Patients with Recurrent Swelling Episodes (AE-QoL), validated in Latvian in March 2022. 13 HAE patients were invited to participate in the study and to answer a written questionnaires and verbal questions in the form of an interview performed by an internist-immunologist or an internist-allergist. Questionnaires covered demographic data and detailed clinical information on HAE. The impact of the available clinical factors on the QoL scores was evaluated. In data analysis, descriptive statistics were used. The reasons that reduce the quality of life the most were analysed for each patient.

**Results:** In AE-QoL questionnaire the highest score was 57.35, and the lowest—0 across all participants. Analysis of the AE-QoL questionnaire revealed that the fears/shame domain was most affected with an average impact of 45.19%, followed by nutrition (37.5%), fatigue/mood (34.62%) and functioning (24.52%).

The unpredictability of the disease reduces the quality of life in all HAE patients, but 90% of patients episodic, chronic nature of HAE disturbed the most. Despite physical recovery between angioedema attacks, patients with HAE continued to experience emotional impairment, resulting in anxiety, feeling of stress, depression and reduced quality of life. The choice of maintenance treatment and prophylaxis drugs are currently limited in Latvia, alternatives are often used in the treatment of HAE, especially for long-term prevention, impairing the effectiveness of treatment in preventing HAE attacks, thus worsening the quality of life.

**Conslusions:** HAE negatively affected patient's lifestyle and quality of life. Fears/shame domain was most affected, followed by nutrition and fatigue/mood. Patients quality of life was most affected by the episodic, unpredictable and chronic nature of HAE and limited access to first-line therapy, especially for long-term prevention.

## P-20 Burden of Hereditary Angioedema Type I and II: Preliminary Results from a Real-world Study in Europe, Israel and Canada

### Henriette Farkas^1^, Emel Aygören-Pürsün^2^, Amin Kanani^3^, Fotis Psarros^4^, Didier Ebo^5^, Noémi Bara^6^, François Gavini^7^, Nawal Bent-Ennakhil^7^, Laura Sayegh^8,^*, Irmgard Andresen^7^

#### ^1^Semmelweis University, Budapest, Hungary; ^2^University of Frankfurt, Frankfurt, Germany; ^3^University of British Columbia, St. Paul’s Hospital, Vancouver, BC, Canada; ^4^Navy Hospital of Athens, Athens, Greece; ^5^Inflamed Centre of Excellence, Antwerp University, Antwerp University Hospital, Belgium; ^6^MediQuest Clinical Research Center, Sangiorgiu de Mures, Romania; ^7^EUCAN Medical Affairs, Takeda Pharmaceuticals International AG, Glattpark-Opfikon, Zürich, Switzerland; ^8^Real-World Evidence, PPD, part of Thermo Fisher Scientific, Montreal, QC, Canada

##### *Correspondence: laura.sayegh@evidera.com

*Allergy, Asthma & Clinical Immunology* 2023, **19**(Suppl 1):P-20

**Rationale:** Hereditary Angioedema (HAE) is a rare, serious, autosomal dominant inherited disorder marked by recurrent, painful, unpredictable, and potentially life-threatening episodes of swelling. The study objective was to understand the real-world burden of HAE and to obtain HAE patients’ perspective on their health-related quality of life (HRQoL).

**Methods:** The observational retrospective cohort study was conducted in Europe, Israel and Canada in patients ≥ 12 years with inadequately controlled HAE type I or II. Patients using lanadelumab as long-term prophylaxis (LTP) were excluded. Medical chart data were abstracted over a 12-month observation period, backward-looking from a qualifying event (QE, the patients’ last documented HAE attack within the eligibility period). In addition, a one-time cross-sectional patient-reported HRQoL survey was distributed after enrollment. All data were collected between 05 April 2022 and 30 November 2022. Analyses were descriptive.

**Results:** Overall, 214 patients were enrolled from 32 HAE centers in 20 countries. Among patients, 58.9% were females, and had a mean (SD) age of 43 (16) at the QE. The majority (94.4%) of patients were ≥ 18 years. Most patients (81.8%) had family history of HAE, and had been diagnosed with HAE an average of 16.4 years before the QE. During the 12-month observation period, the average number of attacks was 9.9, attacks lasted 1.9 days on average and mostly affected the extremities (n = 153, 71.5%) and abdomen (n = 149, 69.6%). Laryngeal/pharyngeal attacks occurred in 22.0% (n = 47) of patients. Patients’ attacks were reported as mild (n = 137, 64.0%), moderate (n = 164, 76.6%), severe (n = 97,45.3%) and life-threatening (n = 21, 9.8%). Nearly all patients (95.4%) had awareness of their attack trigger factors. Ninety-one (42.5%) patients used LTP, and 185 (86.4%) used on-demand treatment with or without LTP. Five (2.3%) patients needed HAE-specific hospitalization and 47 (21.5%) had an emergency room visit. The angioedema quality of life (AE-QoL) questionnaire was completed by 132 patients ≥ 18 years to assess patients’ experiences with angioedema attacks over a 4 week recall period. On a scale of 0 to 100 (higher scores indicating worse QoL), mean (SD) total AE-QoL scores were higher among patients with a higher number of attacks, ranging from 41.1 (23) among patients who reported < 1 attack per month (n = 97) to 69.6 (21) among patients who reported ≥ 3 attacks/month (n = 7).

**Conclusions:** This burden of illness study shows that patients with uncontrolled HAE experience debilitating mild to severe attacks, and have impaired HRQoL. Novel, effective and safe LTP could be beneficial to further reduce HAE burden and optimize patients’ HRQoL.

## Disclosures:

HF reports receiving research grants from CSL Behring, Shire/Takeda and Pharming and consultancy/speaker fees and honoraria from BioCryst Pharmaceuticals, Inc., CSL Behring, Pharming Group NV, KalVista and Shire HGT/Takeda and serves as an advisor and principal investigator for clinical trials/registries for BioCryst Pharmaceuticals, Inc., CSL Behring, Pharming, KalVista and Shire/Takeda. EAP received grants and/or fees as consultant or speaker for Biocryst, Centogene, CSL Behring, Kalvista, Pharming, Pharvaris and Shire/Takeda. AK research grants from Takeda and BioCryst, served on advisory boards for Takeda, BioCryst, Kalvista and CSL Behring and received speaker fees from Takeda and CSL Behring. FP has received speaker and advisory board funding from Takeda Pharmaceuticals, CSL Behring and principal investigator for clinical trials/registries for CSL Behring, Pharming, KalVista and Shire/Takeda. DGE does not have any disclosure. NB has received research grants from Takeda and Pharming and consultancy/speaker fees and honoraria from Pharming Group NV, KalVista and Shire HGT/Takeda and serves as an advisor and principal investigator for clinical trials for BioCryst Pharmaceuticals, Pharming, KalVista and Shire/Takeda. FG, NBE and IA are employees of Takeda and own Takeda stocks. LS is an employee of PPD, part of Thermo Fisher Scientific, Canada.

The study is sponsored by Takeda Pharmaceuticals International AG. Medical writing support for the development of this abstract was provided by Pharmaceutical Product Development (PPD) and funded by Takeda Pharmaceuticals International AG. The authors retain full control of the abstract content. Special thanks to patients who participated in the study and all investigators for their contribution.

## P-21 Assisted reproductive techniques in patients with bradykinin- induced angioedema

### Tatiana Navarro-Cascales^1,2,3^, Olga Roche^1,3^, Rosario Cabañas^1,2,3,4^, Silvia Iniesta Perez^5^, Teresa Caballero^1,2,3,4^

#### ^1^Allergy Department, Hospital Universitario La Paz, Madrid, Spain; ^2^Hospital La Paz Health Research Institute (IdiPaz), Madrid, Spain; ^3^CSUR de Angioedema Hereditario del Hospital Universitario La Paz, Madrid, Spain; ^4^Centre for Biomedical Research Network on Rare Diseases (CIBERER) U754, Hospital Universitario La Paz, Madrid, Spain; ^5^Assisted Reproductive Unit, Department of Gynecology and Obstetrics, Hospital Universitario La Paz, Madrid, Spain

*Allergy, Asthma & Clinical Immunology* 2023, **19**(Suppl 1):P-21

**Introduction:** Bradykinin-induced angioedema (BK-AE) can be due to a deficiency in the C1-esterase inhibitor (C1-INH), which may be hereditary (HAE-C1-INH) or acquired (AAE-C1-INH) or have normal C1-INH, that includes Hereditary Angioedema with *F12* gene mutation (HAE-FXII), among others.

These diseases characterize by unpredictable recurrent angioedema attacks. Some triggering factors have been identified, being estrogens among them.

In women with BK-AE assisted reproductive techniques such as artificial insemination (AI) or in vitro fertilization (IVF) may increase the frequency and severity of attacks due to the increase in both endogenous and exogenous estrogens induced by hormonal treatment.

**Objective:** To describe how assisted reproductive techniques influence the course of BK-AE and to analyze whether long-term prophylaxis (LTP) with plasma-derived human C1-inhibitor (pdC1-INH) during fertility treatment prevents angioedema attacks.

**Material and methods:** Retrospective descriptive study. Review of the course of the disease during assisted fertility techniques in female patients diagnosed with BK-AE, under follow-up in the CSUR (National Reference Center) for Hereditary Angioedema of La Paz University Hospital (Madrid, Spain). The study was approved by the Ethics Committee (PI-4598).

**Results:** Ten women with BK-AE (7 with HAE-C1-INH, 2 with AAE-C1-INH, 1 with HAE-FXII) underwent assisted fertility treatments, 7 of them because of infertility and 3 to avoid disease inheritance.

A total of 24 fertility treatments were performed:

- 9 IVF with own egg: Two were performed with LTP (IV pdC1-INH 1,000U twice a week) with adequate tolerance and 7 without prophylaxis, with increased frequency of angioedema attacks in all of them.
- 3 IVF with preimplantation genetic diagnosis (PGD): All performed with LTP (IV pdC1-INH 1,000U twice a week), 1 without increase of angioedema crises and 2 without attacks.
- 6 IVF with egg donation: Three of them without worsening, of which 2 were performed with LTP (IV pdC1-INH 1000U twice a week). In all those performed without LTP (3) there was an increase in the frequency of angioedema attacks.
- 6 AI: All were performed without LTP; 1 with a slight increase in the number of angioedema attacks and 5 without attacks.

**Conclusion:** Hormonal treatment used for ovarian stimulation and endometrial preparation increases the frequency of angioedema attacks in female patients with BK-AE.

LTP with IV pdC1-INH during fertility treatment is effective in controlling attacks.

Almost all patients tolerated artificial insemination without LTP, what could be due to the hormone treatment being administered at lower doses than those used for in vitro fertilization. More data are necessary in order to advise or not LTP during assisted fertility.

## P-22 Prevalence of non-alcoholic steatohepatitis in adult patients with Hereditary Angioedema due to C1-inhibitor deficiency (HAE-C1-INH)

### Fiorella Adrianzen Alvarez^1^, Tatiana Navarro-Cascales^1,2,3^, Antonio Olveira^4^, Rosario Cabañas^1,2,3,4^, Teresa Caballero^1,2,3,4^

#### ^1^Allergy Department, Hospital Universitario La Paz, Madrid, Spain; ^2^Hospital La Paz Institute for Health Research (IdiPAZ, Group 44), Madrid, Spain; ^3^CSUR Angioedema Hereditario, Hospital Universitario La Paz, Madrid, Spain; ^4^Biomedical Research Network in Rare Diseases (CIBERER U754), Madrid, Spain

*Allergy, Asthma & Clinical Immunology* 2023, **19**(Suppl 1):P-22

**Background:** Hereditary Angioedema due to C1-inhibitor deficiency (HAE-C1-INH) is a rare disease characterized by multiple episodes of angioedema due to the lack of regulation of the contact activation system.

It has been previously proposed that HAE-C1-INH maybe a hepatic metabolic disease, that could increase the risk of development of non-alcoholic steatohepatitis (NASH), even in patients who had never received long-term prophylaxis (LTP) with attenuated androgens (AAs) (stanozolol, danazol).

Although AAs are not currently the first line of treatment as long-term prophylaxis (LTP), they have been widely used in prior years. These drugs have hepatic metabolism and can cause hepatotoxicity as an adverse effect, and so abdominal ultrasound and blood liver enzymes tests are performed to detect adverse events. We therefore decided to compare the prevalence of NASH in patients with HAE-C1-INH who had previously received LTP with AAs, in comparison to those who had not received them.

**Methods:** Retrospective study approved by the Ethics Committee (PI-4598). We included 65 adult patients with confirmed HAE-C1-INH who had at least one abdominal ultrasound with a written report from an echographist available to identify the presence or absence of NASH and had anthropometric data available to calculate their body mass index (BMI). Additionally, we reviewed their clinical history to verify if they had previously received LTP with AAs.

**Results:** Twenty-seven patients (37.5%) had NASH in at least one abdominal ultrasound. Seventy per cent of the patients with NASH had not previously received LTP with AAs, whereas only 48% of those without NASH had not received this therapy (Table 1). XºAdditionally patients with NASH presented a higher BMI median in comparison to those without NASH.

**Conclusion:** Although there are many other factors that contribute to the development of NASH, our findings are consistent with the hypothesis that LTP with attenuated androgens is not a determinant factor that leads to NASH in patients with HAE-C1-INH.

Some limitations of this study are its retrospective design and a possible skew in the prescription of AAs in those patients with NASH, as we considered it a relative contraindication for LTP with attenuated androgens.

**Table 1 (abstract P-22):** Previous LTP with attenuated androgens in patients with HAE-C1-INH with and without NASH

	Patients without NASH	Patients with NASH
Previous treatment with attenuated androgens	23 (51%)	8 (29%)
No previous treatment with attenuated androgens	22 (49%)	19 (70%)
BMI median (kg/m^2^)	25.62	29.12
BMI IQR (kg/m^2^)	3.97	3.77
Female patients	29 (64%)	13 (48%)
Male patients	16 (35%)	14 (52%)
Average age (years)	47.5	52.5
TOTAL	45	27

## P-23 Possible psychological fluctuations in severity and frequency of Hereditary Angioedema swellings

### Tomaz Garcez

#### Immunology Department, Manchester University NHS Foundation Trust, Manchester, UK

*Allergy, Asthma & Clinical Immunology* 2023, **19**(Suppl 1):P-23

**Case report:** Case of a 16 year female old patient that transitioned to our adult service in 2022. The patient initially experienced abdominal symptoms (recurrent vomiting and abdominal pain) in 2012 and then developed swellings of hands and legs in 2013. Hereditary Angioedema (HAE) type 1 was suspected in 2013. C4 and C1-inhibitor levels were found to be variably low. HAE was confirmed by genetic testing with a disease-causing mutation in the *SERPING1* gene c.536C < T, p.Thr179Ile identified in 2014. The patient has never had laryngeal swellings.

The patient developed at least weekly abdominal swellings. Peripheral swellings were infrequent. The family, professionals and clinical psychologists agreed that there was a functional component to the pain and that stress and anxiety worsen the symptoms and can also trigger swellings. School attendance dropped significantly to 25% due to swellings. Various prophylactic interventions were tried, including tranexamic acid (2013—ineffective), regular C1-inhibitor infusions (2016 gradual increasing dose, but still poor control and school attendance) and lanadelumab (2020 with initial improvement in school attendance).

Despite lanadelumab swellings continued requiring treatment with on demand C1-inhibitor concentrate, and therefore lanadelumab was stopped. Confidence with the treating team was impacted by the progress and the patient was transitioned to adult services.

At transition the swelling pattern was one to two swellings per week; 70–80% abdominal; 20–30% predominantly the feet and the hands. The patient recognises the abdominal swellings as being part of HAE rather than an alternative pathology as occasionally there is a rash with the swellings. Treatment response is within two to twenty-four hours dependant on how quickly treatment is used. Response is often quicker for peripheral swellings as they are treated quicker.

The patient completed GCSE examinations and received the required results to progress to college for the preferred course. By review 6 months after transition the patient reported one swelling per month treated with on demand C1-inhibitor concentrate.

**Comment:** The only changes included transition to a different team and moving to a new education environment. At the initial transition consultation, it was agreed to not make any changes to medical management and to simply observe the pattern of swellings for 6 months. Other than psychological factors we have no other explanation for the observed improvement in disease control.

All patients (or their guardians) gave explicit permission for their information to be published.

## P-24 Real-life experience after eight months of long-term subcutaneous C1-inhibitor prophylactic treatment in four Hungarian patients with Hereditary Angioedema due to C1-inhibitor deficiency

### Hanga Réka Horváth^1^, Beáta Visy^1,2^, Henriette Farkas^1^

#### ^1^Hungarian Angioedema Center of Reference and Excellence, Department of Haematology and Internal Medicine, Semmelweis University, Budapest, Hungary; ^2^Heim Pál National Institute of Pediatrics, Budapest, Hungary

*Allergy, Asthma & Clinical Immunology* 2023, **19**(Suppl 1):P-24

**Introduction:** Hereditary Angioedema (HAE) is a rare, autosomal dominant disease that is characterised by recurrent subcutaneous and/or submucosal swellings. The most common form of HAE is caused by the deficiency of the C1-inhibitor (C1-INH) protein. One of the targeted long-term prophylactic treatment options is the substitution of the lacking protein with plasma-derived C1-INH administered subcutaneously twice weekly (sc-pd-C1-INH).

**Objectives:** In our study we aimed to summarise the medical history of four Hungarian patients receiving sc-pd-C1-INH and to assess the effectiveness and safety of the therapy after eight months of follow-up.

**Methods:** Blood and urine tests were taken from all our patients right before the first dose, at one month and at five months of treatment. All four patients filled out an AE-QoL questionnaire right before the first dose and once every month since then. We examined the occurrence of HAE attacks, the number of rescue medications used and the longest attack-free intervals in the eight months before and the eight months since the introduction of therapy. The potential side-effects were also monitored.

**Results:** In our patients (two females (F, aged 33 and 43 years) and two males (M, aged 47 and 61 years)), the complement parameters normalised after the initiation of treatment. None of them experienced any drug-related side effects. In the first month after initiation of treatment, only one patient experienced significant improvement of their quality of life. However, from the second month three out of four patients experienced the same. Two patients (33 y/o F, 47 y/o M), who has had 50 and 23 HAE attacks respectively in the previous 8 months, did not experience any HAE attacks since the introduction of sc-pd-C1-INH. The number of HAE attacks in the other two patients (43 y/o F, 61 y/o M) was reduced to one-fifth during the treatment period (10 instead of 50 and 7 instead of 35, respectively). The two symptom-free patients did not need any rescue medication, while the other two patients needed one-fifth of the previous amount (23 instead of 130 and 8 instead of 39). The longest attack-free period expanded in all four patients (259 days instead of 4, 259 instead of 38, 49 instead of 4 and 67 instead of 14).

**Conclusions:** Long-term prophylaxis with sc-pd-C1-INH is a safe and effective therapy to normalise patients’ complement parameters, thus decreasing the number of HAE attacks and improving patients’ quality of life.

All patients gave explicit permission for their information to be published.

## P-25 Early symptom relief following treatment with the oral bradykinin 2 receptor antagonist PHVS416 in patients with hereditary angioedema attacks

### Marc A. Riedl^1,^*, John Anderson^2^, Emel Aygören-Pürsün^3^, Maria Luisa Baeza^4^, Laurence Bouillet^5^, Hugo Chapdelaine^6^, Danny M. Cohn^7^, Aurélie Du-Thanh^8^, Olivier Fain^9^, Henriette Farkas^10^, Jens Greve^11^, Mar Guilarte^12^, David Hagin^13^, Roman Hakl^14^, Joshua S. Jacobs^15^, Aharon Kessel^16^, Sorena Kiani-Alikhan^17^, Pavlina Králícková^18^, H. Henry Li^19^, Ramon Lleonart^20^, Markus Magerl^21^, Michael E. Manning^22^, Avner Reshef^23^, Bruce Ritchie^24^, Giuseppe Spadaro^25^, Maria Staevska^26^, Petra Staubach^27^, Marcin Stobiecki^28^, Gordon L. Sussman^29^, Michael D. Tarzi^30^, Anna Valerieva^26^, William H. Yang^31^, Marie-Helene Jouvin^32^, Rafael Crabbé^33^, Simone van Leeuwen^34^, Huaihou Chen^32^, Li Zhu^32^, Jochen Knolle^35^, Anne Lesage^36^, Peng Lu^32^, Marcus Maurer^21^

#### ^1^Division of Rheumatology, Allergy and Immunology, University of California, San Diego, La Jolla, CA, USA; ^2^Clinical Research Center of Alabama, AllerVie Health Birmingham, AL, USA; ^3^Department for Children and Adolescents, University Hospital Frankfurt, Goethe University Frankfurt, Frankfurt, Germany; ^4^Allergy Department, Hospital General Universitario Gregorio Marañón, Madrid, Spain; ^5^National Reference Center for Angioedema (CREAK), Department of Internal Medicine, Grenoble Alpes University, Laboratoire T-RAIG, UMR 5525 TIMC-IMAG (UGA-CNRS), Grenoble, France; ^6^CHU de Montréal, Université de Montréal, Montréal, Canada; ^7^Department of Vascular Medicine, Amsterdam Cardiovascular Sciences, Amsterdam UMC, University of Amsterdam, Amsterdam, The Netherlands; ^8^Department of Dermatology, University Montpellier, Montpellier, France; ^9^Department of Internal Medicine, Sorbonne University, AP-HP, Saint Antoine Hospital, Paris, France; ^10^Department of Internal Medicine and Haematology, Hungarian Angioedema Center of Reference and Excellence, Semmelweis University, Budapest, Hungary; ^11^Department of Otorhinolaryngology, Head and Neck Surgery, Ulm University Medical Center, Ulm, Germany; ^12^Allergy Section, Internal Medicine Department, Hospital Universitari Vall d'Hebron, Barcelona, Spain; ^13^Allergy and Clinical Immunology Unit, Department of Medicine, Tel Aviv Sourasky Medical Center and Sackler Faculty of Medicine, University of Tel Aviv, Tel Aviv, Israel; ^14^Department of Clinical Immunology and Allergology, St. Anne's University Hospital in Brno and Faculty of Medicine, Masaryk University, Brno, Czech Republic, Brno, Czech Republic; ^15^Allergy and Asthma Clinical Research, Walnut Creek, CA, USA; ^16^Bnai Zion Medical Center, Technion-Israel Institute of Technology, Haifa, Israel; ^17^Department of Immunology, Royal Free London NHS Foundation Trust, London, UK; ^18^Institute of Clinical Immunology and Allergy, University Hospital Hradec Kralove, Charles University, Faculty of Medicine in Hradec Kralove, Hradec Kralove, Czech Republic; ^19^Institute for Asthma and Allergy, Chevy Chase, MD, USA; ^20^Allergology Service, Bellvitge University Hospital, L'Hospitalet de Llobregat, Barcelona, Spain; ^21^Institute of Allergology, Charité—Universitätsmedizin Berlin, Corporate Member of Freie Universität Berlin and Humboldt-Universität zu Berlin, and Fraunhofer Institute for Translational Medicine and Pharmacology ITMP, Allergology and Immunology, Berlin, Germany; ^22^Allergy, Asthma and Immunology Associates, Ltd., Scottsdale, AZ, USA; ^23^Allergy, Immunology and Angioedema Center, Barzilai University Hospital, Ashkelon, Israel; ^24^Division of Hematology, Department of Medicine, University of Alberta, Edmonton, AB, Canada; ^25^Department of Translational Medical Sciences and Center for Basic and Clinical Immunology Research (CISI), University of Naples Federico II, Napoli, Italy; ^26^Department of Allergology, Clinic of Allergology, University Hospital "Alexandrovska", Medical University of Sofia, Sofia, Bulgaria; ^27^Department of Dermatology, University Medicine Mainz, Mainz, Germany; ^28^Department of Clinical and Environmental Allergology, Jagiellonian University Medical College, Krakow, Poland; ^29^Gordon Sussman Clinical Research Inc, Toronto, Canada; ^30^Department of Medicine, Brighton and Sussex Medical School, Brighton, UK; ^31^Ottawa Allergy Research Corporation, Department of Medicine, University of Ottawa, Ottawa, ON, Canada; ^32^Pharvaris Inc., Lexington, MA, USA; ^33^RC Consultancy, Bassins, Switzerland; ^34^SLC Consultancy, Woerden, The Netherlands; ^35^JCK Consult, Frankfurt, Germany; ^36^GrayMatters Consulting, Schilde, Belgium

##### *Correspondence: mriedl@health.ucsd.edu

*Allergy, Asthma & Clinical Immunology* 2023, **19**(Suppl 1):P-25

**Background:** Excessive activation of bradykinin B2 receptors by bradykinin is the cause of hereditary angioedema (HAE) attacks, manifesting as painful swelling of subcutaneous and submucosal tissue at various body sites. PHA121 is a potent and selective bradykinin B2 receptor antagonist under development for on-demand and prophylactic treatment of HAE.

**Materials and Methods:** RAPIDe-1 was a phase 2, double-blind, placebo-controlled, cross-over, dose-ranging trial of PHVS416, oral softgel capsule formulation of PHA121, for treatment of attacks in HAE due to C1INH deficiency. The Mean Symptom Complex Severity (MSCS) score is a measure of attacks’ symptom severity incorporating the body sites affected and patient-reported symptom severity at each site. The Treatment Outcome Score (TOS) assesses patient-reported response to therapy based on body sites, baseline severity, and response post-treatment dosing. Change in MSCS [minimally important difference (MID): − 0.30] score from pre-treatment to 4 h (h) post-treatment and TOS (MID: 30) at 4 h post-treatment were secondary endpoints of RAPIDe-1 trial.

**Results:** Seventy-four participants were enrolled and 62 of them treated 147 qualifying HAE attacks with study drug (PHVS416 10, 20, 30 mg or placebo) at the time of the primary analysis. MSCS (score decreased) and TOS (score increased) improved during the first 4 h after administration of PHVS416 (all 3 doses) whereas they did not significantly change in placebo-treated attacks. At 4 h, the least squares mean differences of change in MSCS and TOS scores in PHVS416 (10, 20, 30 mg)- and placebo-treated attacks were − 0.79 (nominal p < 0.0001), − 0.61 (p = 0.0008), and − 0.39 (p = 0.0291) and 64.13 (nominal p < 0.0001), 62.69 (p < 0.0001), and 71.06 (p < 0.0001), respectively. Estimated median time for patients to achieve improvement (“a little better”) for all body sites involved by the attacks at 2 consecutive time points on TOS was 1.89, 2.15, and 1.98 h for PHVS416-treated attacks vs. 7.62 h for placebo-treated attacks. Median time to achieve a status of “a lot better or resolved” at all body sites involved was 4.02, 5.93, and 4.12 h for attacks treated with the 3 doses of PHVS416 vs. 23.28 h for placebo-treated attacks.

**Conclusions:** In RAPIDe-1 placebo-controlled trial, treatment with PHVS416 led to more rapid symptom relief and resolution of HAE attacks assessed through the MSCS and TOS, consistently with the outcomes of primary and other secondary endpoints measured through the VAS-3.

**Trial registration**

ClinicalTrials.gov Identifier: NCT04618211. EudraCT Number: 2020-003445-11.

## P-26 Evolution of health-related quality of life in patients with Hereditary Angioedema due to C1-inhibitor deficiency (HAE-C1-INH) and its relationship with disease activity

### Inés Fernández-Concha Llona^1^, Sonia San Martín-Caballero^2^, Itsaso Losantos^2,3^, Tatiana Navarro Cascales^1^, Ana Entrala Bueso^1,2^, Rosario Cabañas^1,2,4,5^, Teresa Caballero^1,2,4^

#### ^1^Allergy Department, La Paz University Hospital, Madrid, Spain; ^2^Health Research Institute of Hospital La Paz (IdiPAZ), Madrid, Spain; ^3^Biostatistics Research Unit, Hospital Universitario La Paz, Madrid, Spain; ^4^Biomedical Research Network on Rare Diseases (CIBERER U754), Madrid, Spain; ^5^Piel en RED, Hospital Universitario La Paz, Madrid, Spain

*Allergy, Asthma & Clinical Immunology* 2023, **19**(Suppl 1):P-26

**Background:** Hereditary Angioedema due to C1-inhibitor deficiency (HAE-C1-INH) has a significant impact on health-related quality of life (HRQoL).

**Material and methods:** Retrospective study in adult patients with HAE-C1-INH between 2016 and 2022. The study was approved by the Ethics Committee (PI-4598). Demographic and clinical data were collected. HRQoL was assessed with HAE-QoL and disease activity with HAE-AS. The first (T1) and last (T2) questionnaires available for each patient were selected, separated by at least 1 year.

**Results:** A total of 76 patients were included, predominantly female (56.6%). The median age at T1 was 41.5 years (P25–75: 35.0–55.0) and at T2 46.5 years (P25–75: 39.0–59.75).

The number of patients that used Long-term prophylaxis (LTP) between T1 and T2 increased (38,2% versus 43.4%). In T1 6.6% used tranexamic acid, 25% attenuated androgens and 6.6% IV plasma derived C1-INH (IV pdC1-INH). In T2 new treatments were available, only 1.3% used tranexamic acid, 21.1% attenuated androgens, 2.6% IV plasma derived C1-INH (IV pdC1-INH), 10% SC pdC1-INH, 6.6% Lanadelumab and 1.3% Berotralstat.

18.4% of the patients started or stopped LTP between T1 and T2. 31.6% changed type of LTP between T1 and T2. 21.1% of the patients had begun to use the new available treatments at T2.

The rescue treatments used in the last 6 months before T1 and T2 were the following: in T1 40.8% used icatibant acetate, 18.4% IV pdC1-INH, 11.8% both and 28.9% none. In T2 51.3% used icatibant acetate, 10.5% IV pdC1-INH, 5.3% both and 32.9% none.

The median total HAE-QoL score was 102.5 (P25–75: 84.0–120) and HAE-AS score was 6 (P25–75: 3.25–8) at T1. The median total HAE-QoL score was 109 (P25–75: 89.25–125) and HAE-AS score was 5.5 (P25–75:3–8) at T2.

HRQoL (HAE-QoL) improved significantly in T2 compared to T1 (p = 0.028). A decrease in disease activity (HAE-AS) was observed in T2 compared to T1, although it did not reach statistical significance (p = 0.079). There was an inverse correlation between total HAE-QoL and total HAE-AS at T1 (-0.665, p < 0.001) and T2 (− 0.565, p < 0.001).

**Conclusions:** There have been changes in the LTP profile. The HRQoL of patients with HAE-C1-INH assessed by HAE-QoL is inversely related to HAE-C1-INH disease activity measured by HAE-AS and has improved significantly. This improvement could be related to the increase in the percentage of patients performing LTP, the change in LTP and a decrease in the activity of the disease.

## P-27 Cardiovascular safety of the orally administered bradykinin B2 receptor antagonist PHA-022121

### Brigitte Loenders^1,^*, Nieves Crespo^2^, Raf Crabbé^3^, Peng Lu^4^, Anne Lesage^5^

#### ^1^BLC, Sint-Huibrechts-Lille (Pelt), Belgium; ^2^Pharvaris GmbH, Zug, Switzerland; ^3^RC Consultancy, Bassins, Switzerland; ^4^Pharvaris Inc., Lexington, USA; ^5^GrayMatters Consulting, Schilde, Belgium

##### *Correspondence: brigitte.loenders@pharvaris.com

*Allergy, Asthma & Clinical Immunology* 2023, **19**(Suppl 1):P-27

**Background**: PHA-022121 (PHA121), an orally bioavailable potent competitive antagonist of the human bradykinin B2 receptor, is being developed for the treatment and prevention of Hereditary Angioedema (HAE) attacks.

**Methods**: The preclinical cardiovascular safety of PHA121 was assessed using in vitro cardiac ion channel and off-target receptor screenings, and in vivo acute and chronic studies in cynomolgus monkeys, as the pharmacologically responsive species. Occurrence of cardiovascular events was monitored in Phase 1 studies of PHA121 and continues to be monitored in ongoing clinical trials in HAE.

**Results**: PHA121 did not significantly inhibit 8 cardiac ion channels (hNav1.5, hKv4.3/KChlP2, hCav1.2, hKv1.5, hKCNQ1/mink, hERG, hHCN4, hKir2.1) in automated patch clamp (inhibition < 25% at 10 μM) and did not notably affect the hERG current in manual whole-cell patch clamp (IC_50_ > 30 μM), at concentrations ≥ 150-fold the unbound PHA121 plasma concentration anticipated at human therapeutic doses.

PHA121 did not elicit cardiac conduction disturbances or arrhythmias in the acute and chronic (up to 39 weeks of dosing) in vivo studies in monkeys and did not affect ECG intervals, morphology and rhythm up to systemic exposures of at least sixfold the anticipated effective concentrations in humans.

Hemodynamic effects were not observed in vivo. Single and repeat oral doses of PHA121 did not induce relevant changes in heart rate (HR), arterial blood pressure (BP) or body temperature in monkeys up to the highest dose tested.

Assessment of cardiac weights, a sensitive measure of muscle mass, and microscopic evaluation of cardiac tissue in the 4-, 13- and 39-week toxicology study in monkeys, revealed no treatment-related adverse effects and no signs of ventricular wall thickness after chronic repeat-dose administration. These data together with the lack of effects on the QRS complex are indicative of the absence of left ventricular hypertrophy, which is consistent with the finding that PHA121 did not relevantly increase BP after long-term administration.

PHA121 was well tolerated in clinical studies in humans. No clinically significant treatment-emergent adverse events were observed in the MedDRA Cardiac disorders SOC, nor dose-, time-, or treatment-dependent changes in ECG-intervals or relevant effects on HR and BP were observed across single- and multiple-dose Phase 1 clinical studies and the Phase 2 on-demand RAPIDe-1 study.

**Conclusions**: PHA121 showed no effect on cardiovascular function in in vitro and in vivo preclinical studies, and in clinical studies completed to date, including acute on-demand and repeat administration up to 10 days at anticipated efficacious doses.

## P-28 Recombinant human C1 esterase inhibitor on-demand treatment for attacks of Hereditary Angioedema: a European registry update

### Anna Valerieva^1^, Maria T. Staevska^1^, Vesna Grivcheva-Panovska^2^, Miloš Ješenak^3^, Kinga Viktória Kőhalmi^4,5,6^, Katarina Hrubiskova^7^, Andrea Zanichelli^8^, Drasko Cikojević^9^, Luca Bellizzi^10^, Anurag Relan^11^, Roman Hakl^12^, Henriette Farkas^4^

#### ^1^Department of Allergology, Medical University of Sofia, Sofia, Bulgaria; ^2^PHI University Clinic of Dermatology, School of Medicine, University Saints Cyril and Methodius, Skopje, North Macedonia; ^3^National Center for Hereditary Angioedema, Jessenius Faculty of Medicine, University Hospital in Martin, Comenius University in Bratislava, Martin, Slovakia; ^4^Hungarian Angioedema Center of Excellence and Reference, Department of Internal Medicine and Haematology, Semmelweis University, Budapest, Hungary; ^5^Department of Rheumatology and Clinical Immunology, Semmelweis University, Budapest, Hungary; ^6^Hospital of Hospitaller Brothers of St. John of God, Budapest, Hungary; ^7^Comenius University in Bratislava and University Hospital, Bratislava, Slovakia; ^8^ASST Fatebenefratelli Sacco, Ospedale Luigi Sacco-University of Milan, Milan, Italy; ^9^Department of Otorhinolaryngology, University Hospital of Split, Split, Croatia; ^10^Pharming Technologies BV, Leiden, The Netherlands; ^11^Pharming Healthcare Inc., Warren, NJ, USA; ^12^St. Anne’s University Hospital, and Masaryk University, Brno, Czech Republic

*Allergy, Asthma & Clinical Immunology* 2023, **19**(Suppl 1):P-28

**Background:** Hereditary Angioedema (HAE) due to C1-inhibitor deficiency (C1-INH-HAE) causes recurrent episodes of disabling, painful swelling. Recombinant human C1 esterase inhibitor (rhC1-INH; Ruconest) is approved in multiple countries for the on-demand (acute) treatment of HAE attacks. An ongoing treatment registry for multiple European countries has been examining the efficacy and safety of rhC1-INH.

**Methods:** Patients with C1-INH-HAE were enrolled following a decision to treat with rhC1-INH and acquisition of written informed consent. Medical history and baseline HAE information were obtained at screening. Treatment decisions were at the discretion of the health care providers (HCPs) involved in the patients’ care, according to the HCPs’ standards for the management of C1-INH-HAE, and in line with the approved rhC1-INH summary of product characteristics. Using a web-based questionnaire, HCPs entered data on HAE attacks, response to therapy, and adverse events (AEs) following treatment.

**Results:** From 01 July 2011 through 01 January 2023, 92 patients with C1-INH-HAE (37 male/55 female; current mean age, 51 years; current age range, 18–82 years) in 9 countries reported 3599 HAE attacks and were treated with rhC1-INH within the registry. The mean age at HAE diagnosis was 27 years (range, 3–78 years). Before registry enrolment, patients, including 26 (28.3%) who were on maintenance therapy/prophylaxis at registry enrolment, experienced a mean of 30 HAE attacks the previous year. Since enrolment there have been 1544 (42.9%) abdominal, 1294 (36.0%) peripheral, 498 (13.8%) oro-facial-pharyngeal, 229 (6.4%) urogenital, and 181 (5.0%) laryngeal HAE attacks; of these, 133 attacks involved 2 locations and 7 involved 3 locations. The mean rhC1-INH dose was 3417 U (43.8 U/kg). Patients reported resolution of 97.8% of HAE attacks (3520/3599) with rhC1-INH within 4 h; most HAE attacks (99.8%; 3593/3599) required only 1 dose of rhC1-INH. Six HAE attacks were treated with a second dose (total rhC1-INH administered to treat attack, 4200 U). No hypersensitivity or thrombotic/thromboembolic events or drug-related serious AEs were reported.

**Conclusion:** This rhC1-INH treatment registry continues to provide real-world data on the on-demand (acute) treatment of HAE attacks and supports the efficacy and safety of rhC1-INH across multiple HAE attack locations in individuals with C1-INH-HAE.

**Disclosures**

A Valerieva reports serving as a consultant for Pharming Group NV and receiving symposium sponsorship from CSL Behring, Sobi, and Takeda Pharmaceutical Co. Ltd./Shire.

M.T. Staevska reports receiving consultancy/speaker honoraria from Pharming Group NV and Sobi.

V. Grivcheva-Panovska reports serving as principal investigator for clinical trials sponsored by Pharming Group NV.

M. Ješenak reports receiving consultancy/speaker honoraria from CSL Behring, Sobi, and Takeda Pharmaceutical Co. Ltd./Shire; and serving as a principal investigator for clinical trials sponsored by BioCryst Pharmaceuticals, Inc. and Pharming Group NV.

K. Viktória Kőhalmi reports receiving consultancy/speaker honoraria from CSL Behring and Takeda Pharmaceutical Co. Ltd./Shire.

K. Hrubiskova reports serving as co-investigator for clinical trials sponsored by Pharming Group NV and receiving consultancy honoraria from Takeda Pharmaceutical Co. Ltd.

A. Zanichelli reports receiving consultancy/speaker honoraria from BioCryst, CSL Behring, Pharming Group NV, and Takeda Pharmaceutical Co. Ltd.; and serving as a principal investigator for clinical trials/registries for BioCryst, CSL Behring, KalVista, Pharming Group NV, and Takeda Pharmaceutical Co. Ltd.

D. Cikojević reports serving as principal investigator for clinical trials sponsored by Pharming Group NV.

L. Bellizzi is a medical advisor for Pharming Technologies BV.

A. Relan is an employee of Pharming Healthcare Inc.

R. Hakl reports receiving consultancy/speaker honoraria from CSL Behring and Takeda Pharmaceutical Co. Ltd./Shire; and serving as a principal investigator for clinical trials sponsored by CSL Behring, BioCryst Pharmaceuticals, Pharming Group NV, and KalVista Pharmaceuticals.

H. Farkas reports receiving research grants from CSL Behring, Pharming Group NV, and Takeda Pharmaceutical Co. Ltd./Shire; serving as an advisor for BioCryst Pharmaceuticals, Inc., CSL Behring, KalVista, Pharming Group NV, and Takeda Pharmaceutical Co. Ltd./Shire Human Genetic Therapies and receiving consultancy/speaker fees and honoraria from these companies, and as a principal investigator for clinical trials/registries for BioCryst, CSL Behring, KalVista, Pharming Group NV, and Takeda Pharmaceutical Co. Ltd./Shire.

## P-30 Genetic segregation study in Hereditary Angioedema with normal C1-inhibitor due to F12 mutation in Southern Spanish population- an observational study

### Krasimira Baynova^1,^*, Teresa De Aramburu ^1^, Raul García^2^, Teresa González-Quévedo^1^, Stefan Cimbollek^1^

#### ^1^Department of Allergy, National Unit of Angioedema, University Hospital Virgen del Rocío, Seville, Spain; ^2^Department of Immunology, National Unit of Angioedema, University Hospital Virgen del Rocío, Seville, Spain

##### *Correspondence: krasi1024@yahoo.com

*Allergy, Asthma & Clinical Immunology* 2023, **19**(Suppl 1):P-30 

**Background:** Hereditary Angioedema with normal C1-inhibitor due to F12 mutation /FXII-HAE/ has an autosomal dominant mode of inheritance with low penetration. In 2006, two different missense mutations located in the same position in exon 9 of the F12 gene (coding factor XII [FXII] HAE) were described in approximately 25% of a cohort of nC1-INH-HAE patients. The most common of the 2 mutations predicts a substitution of threonine by lysine in the secreted zymogenic protein (c.983C > A, p.Thr309Lys, also called p.Thr328Lys with the addition of the leader protein), while the second mutation predicts a substitution of threonine by arginine (c.983C > Gp.Thr309Arg).

The objectives of this study were to describe and characterize the phenotypic characteristics of individuals from southern Spain who share the same mutation in F12 gene, p.Thr309Lys, and to investigate new genetic variants that will provide us with information to explain the phenotypic differences we find with individuals who present the same mutation, even if they are members of the same family.

**Materials and methods:** This was a descriptive, prospective and observational study conducted at the Angioedema Reference Unit of the Virgen del Rocío University Hospital in Seville, southwestern Spain. We studied 10 not related families.

After an initial clinical evaluation, patients were assigned to three phenotype groups: asymptomatic carriers, paucisymptomatic patients (those who had experienced a single episode or sporadic episodes of HAE) and symptomatic patients. We evaluate the association between these clinical phenotypes and genetic characterization based on the presence of different variants in relation to genes possibly involved in the pathogenesis of the angioedema and/or clinical phenotype.

A customized NGS panel was designed using the Scientific Designer Ion AmpliSeq Thermo Fisher, in order to analyze 45 genes (all coding regions and exon–intron junctions) possibly involved in the pathogenesis of angioedema and/or the clinical phenotype.

**Results:** All the patients (n37) were positive for the F12 gene mutation p.Thr328Lys( heterozygous). 64,9% (n24) of the patients were symptomatic. We detected 9 variants in 8 of the studied 45 genes with very low prevalence.

In 60% of the families there is inheritance only for the F12 mutation, not for the rest of the variants.

In those where there is segregation, it will be necessary to investigate in the future whether this has repercussions on the phenotypic differences that we can find between the different patients with F12 mutation.

**Conclusions:** No clear correlation between phenotype and genotype was found in our group of patients with Hereditary Angioedema with normal C1-inhibitor due to F12 mutation.

## P-31 Case report: Off-label long-term prophylaxis with C1-esterase-inhibitor (C1-INH) s.c. in a patient with acquired C1-INH-deficiency (C1-INH-AAE)

### Eva-Vanessa Ebert, Felix Johnson, Amir Bolooki, Anne Ruck, Michael Krokenberger, Barbara Wollenberg, Susanne Trainotti

#### Department of Otorhinolaryngology, Klinikum rechts der Isar, Technical University Munich, Germany

*Allergy, Asthma & Clinical Immunology* 2023, **19**(Suppl 1):P-31

**Objective:** Hereditary Angioedema (HAE) is a rare genetic disease characterised by recurrent swelling of the skin, mucosa and abdomen. Much rarer than HAE are angioedemas caused by increased consumption of C1-INH, e.g. in the context of lymphatic or autoimmune diseases, called acquired C1-INH-deficiency (C1-INH-AAE). There is currently no approved treatment for C1-INH-AAE, so off-label treatment is based on HAE therapy.

We present the successful off-label use of C1-INH s.c. for long-term prophylaxis (LTP) in a patient with C1-INH-AAE.

**Methods:** The frequency and localisation of attacks were recorded using the attack diary for 6 months during on-demand therapy (ODT) with Icatibant 30 mg s.c. (M0), as well as 1 and 6 months (M1, M6) after initiation of LTP with C1-INH s.c.. In addition, treatment control and quality of life were assessed using the validated questionnaires AECT and AE-QoL at M0, M1 and M6.

**Results:** A 68-year-old female patient suffered from recurrent swellings since 2014. Blood tests showed decreased C1-INH concentration and activity and C1q. The initial diagnosis of C1-INH-AEE was made in 2015 and a mutation in the *SERPING1* gene was excluded. Repeated (haemato-)oncological investigations showed no abnormalities. Since 2014, an ODT with Icatibant 30 mg s.c. was established. In 2021, the attack frequency had increased significantly to 5 attacks/month. The patient showed a highly impaired quality of life (AE-QoL 98.53) and low therapy control (AECT 2), so LTP was indicated. Because of concomitant medication with tricyclic antidepressants, therapy with C1-INH 3000 IU s.c. twice a week was initiated. In the following 6 months (M6) only 2 attacks occured. At M1 AECT showed complete disease control (16) and AE-QoL showed an improved quality of life (61.7). Due to a therapy break after M6, the number of attacks increased, AE-QoL of 85.29 and AECT of 8 worsened, so LTP was resumed.

**Discussion:** This case clearly shows that even in the off-label setting of C1-INH-AAE, treatment with C1-INH-LTP can effectively reduce the attack frequency and achieve good disease control and improve quality of life. Due to the small number of cases in this rare clinical condition, the inclusion of these patients in OLE or observational studies would be desirable.

All patients gave explicit permission for their information to be published.

## P-32 Specific anti-spike IgG subclasses in patients with C1-INH-HAE (C1-esterase inhibitor deficiency Hereditary Angioedema) after different types of COVID-19 vaccines

### Petra Kiszel^1^, Pál Sík^2^, Erika Kajdácsi^1,2^, György Sinkovits^2^, Henriette Farkas^2^, László Cervenak^2^, Zoltán Prohászka^1,2^

#### ^1^Research Group for Immunology and Hematology, Semmelweis University-Eötvös Loránd Research Network (Office for Supported Research Groups), Budapest, Hungary; ^2^Department of Internal Medicine and Hematology, Semmelweis University, Budapest, Hungary

*Allergy, Asthma & Clinical Immunology* 2023, **19**(Suppl 1):P-32

Vaccination is the most effective preventive procedure to reduce the hospitalizations and the associated complications with severe COVID-19 disease. To monitor the specific anti-spike IgG subclasses after third COVID-19 vaccination in patients at risk of angioedema formation will provide additional details about specific long term immune memory and vaccination efficacies.

Our study aims to show the levels of specific anti-spike IgG subclasses and their contributions to all spike specific IgGs in three patients with C1-INH-HAE. Herein, we report three cases of C1-INH-HAE patients. One of them received three Pfizer BioNTech mRNA vaccines, who was also naturally infected by SARS-CoV-2 at 37 days before sampling timepoints. Two of our patients received two Sputnik V Gam-COVID-Vac vector-based pimary immunizations and one Pfizer BioNTech booster vaccination. Their serum samplings were taken after booster shot at median days 141. The quantitative determination of specific anti-spike IgG subclasses was assessed by in-house ELISAs and total not specific IgG subclasses were determined by nephelometry.

The dominance of spike specific IgG1 was found in patients who received vector-based primary immunizations. The appearance of anti-spike IgG2 and IgG4 antibodies were only characteristic in mRNA vaccinated patient, who were also infected after their basic immunizations. The contributions of spike specific IgG subclasses to all spike specific IgGs showed the same pattern in C1-INH-HAE patients than in our healthy controls. The concentrations of total not specific IgG subclasses showed also normal values.

Taken together, similar spike specific IgG subclass distributions were found in three patients with C1-INH-HAE than in healthy Hungarian cohort. Further studies are needed whether the high contribution of spike specific IgG4 antibody response after mRNA vaccination may affect the antibody-dependent complement activation in patients with C1-INH deficiency at a breakthrough infection.

## P-33 Patients with Hereditary Angioedema comply with regular immunization? Pilot study

### Mylena Menezes da Silva^1^, Anete Sevciovic Grumach^2^

#### ^1^University Center FMABC, Santo Andre, SP, Brazil; ^2^Clinical Immunology, Department of Clinical Medicine, University Center Faculty of Medicine ABC, Santo Andre, SP, Brazil

*Allergy, Asthma & Clinical Immunology* 2023, **19**(Suppl 1):P-33

**Introduction:** Vaccination in public health is considered one of the most effective methods of health prevention. For patients with Hereditary Angioedema (HAE) as well as the population, this preventive action is essential. However, due to a lack of knowledge about the risks and benefits of immunization, outdated vaccination may occur. With that in mind, the **aim** of this study was to evaluate the vaccination coverage of HAE patients and their fears.

**Methods:** patients with confirmed HAE diagnosis followed in our outpatient clinic responded to a questionnaire which included clinical characteristics of the disease and registry of vaccines received.

**Results:** the data were collected from 25 patients, aged between 15 and 68 years; 88% [22/25] were female and 12% [3/25] were male; 60% [15/25] of HAE type 1 and 40% [10/25] of HAE with normal C1-INH. Vaccination coverage was defined according to the complete immunization recommendations of the SBIm (Brazilian Society of Immunizations) and compared with official Brazilian registry (BR). The vaccine coverage was against: BCG [12/24; 50% vs BR 52%]; Inactivated Poliovirus [14/25; 56% vs BR 51%]; Oral poliovirus [11/25; 44% vs BR 51%]; Diphtheria, pertussis and tetanus [13/25; 52% vs BR 39%]; Meningococcus ACWY [1/25; 4% vs BR 22%]; Meningococcus B [2/25; 8% vs BR 47%, approximately]; Meningococcus C [2/25; 8% vs BR 53%]. As for pneumococcal vaccine, the coverage against PCV10, PCV13, and PPSV23 was 0% for complete immunization [vs BR 89%, approximately]. In addition, the vaccine coverage against measles, mumps, rubella was [7/25; 28% vs BR 57%], HPV [3/25; 12% vs BR 35%], hepatitis B [16/25; 64% vs BR 55%]; no one received hepatitis A [vs BR 52%] and Covid-19 was the most prominent coverage [20/25; 80% vs BR 50%]. Anxiety during vaccination was reported by 8% [2/25].

**Conclusion:** For patients in this study, fear of vaccination was not a major obstacle to immunize. Although vaccination against hepatitis A and B is recommended by all guidelines, there was no coverage for hepatitis A and 1/3 of the patient did not receive the vaccine for hepatitis B. The coverage of HAE population is comparable to the Brazilian population, reflecting the restricted access to vaccines not offered by public helath system. Higher compliance to COVID-19 vaccination probably reflected in the inclusion of HAE as an immunodeficiency.

## P-34 C1-INH complexes as markers for classical and lectin pathway activation and complex levels in angioedema patients

### Lisa Hurler^1^, Erika Kajdácsi^1^, Erik J.M. Toonen^2^, Bregje van Bree^2^, György Sinkovits^1^, László Cervenak^1^, Reinhard Würzner^3^, Zoltán Prohászka^1,4^

#### ^1^Department of Internal Medicine and Hematology, Semmelweis University, Budapest, Hungary; ^2^Research and Development Department, Hycult Biotech, Uden, The Netherlands; ^3^Institute of Hygiene and Medical Microbiology, Medical University of Innsbruck, Innsbruck, Austria; ^4^Research Group for Immunology and Hematology, Semmelweis University—Eötvös Loránd Research Network (Office for Supported Research Groups), Budapest, Hungary

*Allergy, Asthma & Clinical Immunology* 2023, **19**(Suppl 1):P-34

C1-inhibitor (C1-INH) complexes have been identified as potential markers for the activation of the complement system and form when C1-INH binds to activated serine proteases, such as C1r and C1s of the classical pathway and MASP-1 and -2 of the lectin pathway.

Since no validated markers were available commercially to differ between early classical and early lectin pathway activation, we aimed to develop and validate immunoassays measuring C1s/C1-INH complex and MASP-1/C1-INH complex as biomarkers for specific early classical and early lectin pathway activation, respectively. In addition, we utilized those assays to measure C1-INH complex concentrations in healthy individuals and pathological samples.

Immunoassays were successfully developed and commercialized by Hycult Biotech. Activation experiments in vitro showed pathway-specific C1s/C1-INH or MASP-1/C1-INH complex formation in human serum. Next to that, a first reference range in healthy adults was determined and C1-INH complex levels were altered in pathological samples of individuals with complement-mediated diseases.

The experiments showed that measuring C1s/C1-INH and MASP-1/C1-INH complex levels provides additional information about specific activation of the classical or lectin pathway. In the future, those immunoassays will also be utilized in a first study in angioedema patients.

## P-35 Organization and patient activity

### Elena Bezbozhnaya

#### Society of Patients with Hereditary Angioedema, Chairman of the Management Board patient organization, Moscow, Russia

*Allergy, Asthma & Clinical Immunology* 2023, **19**(Suppl 1):P-35

What is the most important thing for any patient with Hereditary Angioedema?—For the fullness of life, first of all, he needs modern medicines, which he must have with him at any time. The availability of improved treatments and disease management over the past decade makes complete control of HAE a real possibility for most patients. But this does not happen automatically.

The main problem Russian HAE patients face is limited access to innovative treatment including long-term prophylaxis treatment which is extremely important for HAE nosology. To address an identified issue HAE Society Russia represented by Elena Bezbozhnaya implements a complex approach aimed at awareness raising, patients’ interests advocation and targeted crisis assistance provision. HAE Society Russia has already managed to ensure sustainable funding of HAE children via Circle of Kindness Foundation and now focuses on improvement of access to treatment of adult patients and their navigation after attainment of majority.

The general trend for countries with developed systems of medicine should be to increase the participation of non-profit patient organizations, as controllers of the quality of medical care, in the discussion and development of legislation related to ensuring the rights of patients. Moreover, HAE Society Russia increases its patients’ awareness on regular basis via holding education events and elaborating information materials as well as promotes patient positive experience via creating patient videos. Furthermore, HAE Society also provides its patients with legal and psychological assistance if needed. The assessment of HAE Society performance might be premature, however, preliminary results proved to effectively address patients’ unmet needs.

Patient pools can be considered a new kind of pressure group with sociological point of view. They have a unique opportunity to voice needs and interests patients from the first person at the local, national and global levels.

## P-36 Hereditary Angioedema with normal C1-inbibitor and cutis laxa: an unusual association

### Regis de Albuquerque Campos^1^, Jéssica Branquinho^2^, Clarissa Azevedo Bittencourt^2^, Joice Trigo da Fonseca^3^, Joanemile Pacheco de Figueiredo^1^, João Bosco Pesquero^2^

#### ^1^Department of Internal Medicine, Federal University of Bahia, Salvador-BA, Brazil; ^2^Department of Biophysics, Federal University of São Paulo, São Paulo, Brazil; ^3^Bahia Medical School, Federal University of Bahia, Salvador-BA, Brazil

*Allergy, Asthma & Clinical Immunology* 2023, **19**(Suppl 1):P-36

**Background:** Hereditary Angioedema (HAE), is an autosomal dominantly inherited disease, leading to unpredictable swelling episodes due to increased vascular permeability in skin, respiratory, gastrointestinal systems. HAE classified into HAE with C1-inhibitor (C1-INH) deficiency and HAE with normal C1-inhibitor (HAE-nC1-INH) that has six different genotypes recognized. Cutis laxa syndromes are a group of heterogeneous disorders clinically characterized by loose, redundant skin folds. Excessive skin folds are a consequence of dermal elastic fiber fragmentation, the histological hallmark of cutis laxa. Congenital cutis laxa results from monogenic defects that impair elastic fiber assembly while acquired cutis laxa results from proneness to elastic fiber degradation.

**Case Report:** We describe a patient from Brazil, female, 57 years old. In June 2015, she noticed progressive sagging of the skin on the chest, abdomen and especially the face, with excessive wrinkling. After 2 months, she developed facial angioedema after Zika virus infection and after 1 year, she started to present hives associated with skin swellings with poor improvement with H1-antihistamines treatment. In 2017, she had a facial plastic surgery to improve her sagging skin, but without efficacy. The following year, she was diagnosed with cutis laxa and chronic spontaneous urticaria after skin biopsy, but even at fourfold doses of H1 antihistamines there was no improvement. In 2020, she started the treatment with omalizumab with resolution of the hives but no change in frequency of angioedema which affected mostly the face. Thus, after 6 months, the omalizumab administration interval was reduced from four to two weeks, but the treatment was discontinued due to the still recurrent facial swelling. In 2021, whole-exome sequencing was performed which identified a rare heterozygous missense variant (p.Thr200Ala) in exon 6 of the plasminogen gene (PLG). The presence of this variant was confirmed by Sanger sequencing and was also detected in her two asymptomatic sons and her twin sister, who have been affected with less frequent attacks of swelling, but did not have urticaria or clinical findings of cutis laxa. The patient was treated with tranexamic acid with no response and started with lanadelumab treatment for long-term prophylaxis and icatibant for acute attacks with a better outcome.

**Conclusion:** Through the clinical history of the patient and evidence in the literature regarding HAE-nC1-INH, we believe that this variant in PLG could lead to clinical angioedema phenotype found. Probably the association of HAE with cutis laxa might explain the recurrent attacks of swelling.

All patients gave explicit permission for their information to be published.

## P-37 Development of HAE awareness in the Czech Republic

### Camelia Isaic^1,*^, Anežka Dašková^2^

#### ^1^HAE Junior patient organization, Czech Republic; ^2^First Faculty of Medicine, Charles University, Prague, Czech Republic

##### *Corresppondence: cisaic@haejunior.cz

*Allergy, Asthma & Clinical Immunology* 2023, **19**(Suppl 1):P-37

**Introduction:** Low levels of rare disease awareness can lead to diagnosis delay, misdiagnosis and/or negatively impact the quality of everyday life of patients living with such diseases. The need to raise awareness about Hereditary Angioedema (HAE), a rare genetic disease, has been identified as prioritary in HAE Junior’s patient survey conducted in early 2020 [1] in the Czech Republic. Since then multiple HAE awareness initiatives took place, leading to potential changes compared to 2020.

**Objective:** The objective of this study is to assess the development of HAE awareness in the Czech Republic from a patient/ carer perspective.

**Materials and methods:** In January 2023, HAE Junior patient organization conducted an online patient survey on the topic of *Development of awareness of HAE in the Czech Republic*. A total of 15 HAE patients/ carers participated in the survey, representing 4 men and 11 women. Additional social media analytics, media monitoring statistics and third party research sources were evaluated to complement the HAE patient perspective.

**Results:** According to the survey, 10 respondents reported that the level of HAE awareness/ knowledge has improved among their own family members, while 8 respondents reported higher levels among healthcare and school personnel from their local communities.

A total of 9 respondents indicated that their quality of life has improved also thanks to a higher level of HAE awareness/ knowledge in their community.

The survey participants were also asked what lead/ could lead to improvement of HAE awareness/knowledge in their own community. Most respondents (11 out of 15) indicated social media influencers’ support, while 10 out of 15 indicated the promotion of patient stories.

**Conclusions:** To conclude, the survey indicates an improved level of HAE awareness and quality of life from a patient/ carer perspective in the Czech republic. These findings are encouraging, especially that these patient/career perceived improvements occurred during times of crisis (covid-19 pandemic, war), when other topics preoccupied most of the population.

**References**

1. Isaic, poster HAE Junior: A patient organization with a holistic approach presented at 12^th^ C1-inhibitor Deficiency and Angioedema Workshop, Budapest, June 2021

## P-38 Efficacy of the oral bradykinin B2 receptor antagonist PHVS416 by attack symptom in the RAPIDe-1 phase 2 clinical trial for treatment of Hereditary Angioedema attacks

### Anna Valerieva^1^, Giorgio Giannattasio^2^

#### ^1^Department of Allergology, Medical University of Sofia, Sofia, Bulgaria; ^2^Pharvaris GmbH, Zug, Switzerland

*Allergy, Asthma & Clinical Immunology* 2023, **19**(Suppl 1):P-38

No abstract has been submitted.

## P-39 First report of FXII mutation in a Mexican family with Hereditary Angioedema

### Francisco Alberto Contreras-Verduzco^1^, Gabriel E. Arce-Estrada^2^, Sandra A. Nieto-Martínez^3^, Joao Bosco Pesquero^4^, Anete S. Grumach^5^

#### ^1^Médico Adscrito al Servicio de Alergia del Instituto Nacional de Pediatría, Ciudad de México, México; ^2^Programa de Maestría y Doctorado en Ciencias Médicas y Odontológicas de la Universidad Nacional Autónoma de México (UNAM), Instituto Nacional de Pediatría, Ciudad de México, México; ^3^Unidad de Genética de la Nutrición, Instituto Nacional de Pediatría, Ciudad de México, México; ^4^Center for Research and Molecular Diagnostic of Genetic Diseases, Department of Biophysics, Federal University of São Paulo, São Paulo, Brazil; ^5^Faculdade de Medicina ABC, University Center FMABC, São Paulo, Brazil

*Allergy, Asthma & Clinical Immunology* 2023, **19**(Suppl 1):P-39

**Background:** Hereditary Angioedema (HAE) is a rare genetic disease with either a quantitative or qualitative deficiency in C1-inhibitor (C1-INH) or normal C1-INH [1]. Characterize by recurrent localized edema in various organs, which can be potentially fatal, like laryngeal attacks. In patients with HAE with nC1-INH 6 pathogenic variants have been identified in factor XII (HAE-FXII), angiopoietin-1 (HAE-ANGPT1), plasminogen (HAE-PLG), kininogen 1 (HAE-KNG1), myoferlin (HAE-MYOF), and heparan sulfate-glucosamine 3-O-sulfotransferase 6 (HAE-HS3ST6) [2]. Approximately 30% of these cases are due to factor XII pathogenic variants. Point mutation (Thr328Lys or Thr328Arg), a large deletion (deletion of 72 base pairs: c.971_1018 + 24del72*) or an 18-bp duplication in the factor XII gene are detected in HAE-FXII [3].

**Case report:** A 47 years old woman, started with edema from the age of 20 on the lips, eyelids and the all face lasting 3 days, in addition to laryngeal edema at least 1 per year treated with steroids and antihistaminic with mild improvement. The attacks increase after the placement of a contraceptive subdermal implant. In September 2020, she presented laryngeal attack, cardiorespiratory arrest that required advanced resuscitation maneuvers and ventilatory assistance for 72 h. The patient currently has neurological sequelae such as blindness, limited speech, gait disturbance and mild ataxia. Due to continuing with the edemas despite normal complement values, she went to the consultation for a new assessment and during the consultation, she reports that his mother, sisters and nephews have edema attacks also.

**Methods:** Die blood spots on filter paper (Guthrie cards) were collected from each of the symptomatic and suspected relatives of the index case family. Quantitative and functional C4, C1-INH (normal) values were taken from the patient history. Genetic test for the detection of pathogenic variants were performed to analyze exon 9, its borders and splice sites.

**Results:** The presence of the most common pathogenic variant in the FXII gene was evidenced for 12 patients (8 women and 4 men) from 18 samples, see the family pedigree. Figure 1. c.983C > A heterozygous (p.Thr328Lys). A man still without manifestations. There are still relatives to study.

**Figure 1 (abstract P-39):**
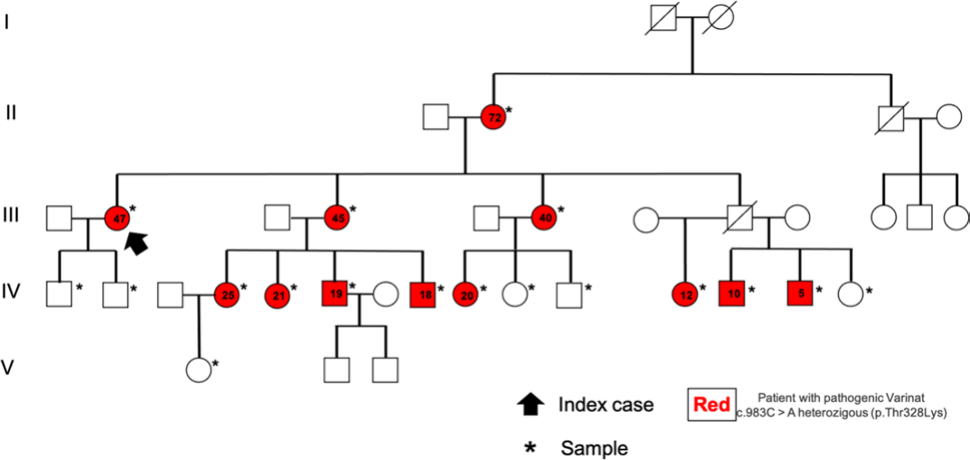
Pedigree of the HAE-FXII family

**Conclusions:** We describe the first Mexican family with HAE-nC1-INH with a pathogenic variant in the FXII gene and according to the evolution of the patients, the attacks are related to hormone levels in women and stress in men. Due to the lack of timely diagnosis, the index patient has severe neurological sequelae secondary to laryngeal attack.

All patients gave explicit permission for their information to be published.

**References**

1. Bork K, Diagnosis and treatment of Hereditary Angioedema with normal C1-inhibitor. All Asth Clin Immun 6, 15 (2010).
2. Bork K, Wulff K, Möhl BS, Steinmüller-Magin L, Witzke G, Hardt J, Meinke P. Novel Hereditary Angioedema linked with a heparan sulfate 3-O-sulfotransferase 6 gene mutation. J Allergy Clin Immunol. 2021 Jan 25:S0091-6749(21)00094-4.
3. Bork K, Wulff K, Hardt J, et al. Hereditary Angioedema caused by missense mutations in the factor XII gene: clinical features, trigger factors, and therapy. J Allergy Clin Immunol. 2009;124(1):129-134.

## P-40 Hereditary Angioedema with a mutation in the plasminogen gene

### Signe Purina^1^, Adine Kanepa^2,3^, Guna Ziedone^1,3^, Dmitrijs Rots^3^, Linda Gailite^3^, Natalja Kurjane^2,3^

#### ^1^Center for the Diagnostics and Treatment of Allergic Diseases, Riga, Latvia; ^2^Pauls Stradiņš Clinical University Hospital, Latvia; ^3^Riga Stradiņš University, Latvia

*Allergy, Asthma & Clinical Immunology* 2023, **19**(Suppl 1):P-40

**Background:** In most cases Hereditary Angioedema (HAE) is associated with a deficiency of C1 esterase inhibitor (C1-INH) caused by a pathogenic variant in the *SERPING1*. There is difficulty in making an accurate diagnosis when C1-INH level and activity are normal (n-C1-INH HAE). The aim of this case report was to demonstrate the first n-C1-INH HAE patient from Latvia with identified pathogenic variant in the *PLG* gene.

**Case report:** A 40-years-old female at the age of eight began experiencing rare, irregular mild episodes of swelling in the extremities lasting several days and recurrent abdominal pain for no apparent reason. After 30 years of age she had mild episodes of edema during pregnancy and while taking oral contraceptives. No family history. In June 2022, she was admitted to the hospital with acute attacks of swelling of the throat and tongue, which were explained by a food allergy. The patient had a proven allergy to citrus fruits and sweet peppers, which were excluded from the diet. Therapy with antihistamines and glucocorticoids had a minimal effect. In 2022, at the age of 39, C4 level, C1-INH level and activity were performed. All determined parameters were within normal ranges. DNA analysis were performed for *SERPING1* gene and pathogenic variant hotspots in *ANGPT1*, *F12* and *PLG.* Pathogenic variant in heterozygous state were identified in *PLG* gene (NM_000301.5:c.988A > G p.(Lys330Glu) was detected)—molecularly confirming the diagnosis of n-C1-INH HAE.

**Conclusions:** This case demonstrates a delay in the diagnosis of n-C1-INH HAE as it manifests with mild attacks, normal C1-INH level and activity, no family history, and symptoms overlapping with allergy.

All patients gave explicit permission for their information to be published.

## P-41 Experience in the use of pathogenetic therapy for arresting Hereditary Angioedema (HAE) attacks in pediatric patients

### Ekaterina A. Viktorova, Natalya B. Kuzmenko, Yuliya Rodina, Anna A. Mukhina, Iurii A. Shifrin, Anna Shcherbina

#### Dmitry Rogachev National Medical Research Center Of Pediatric Hematology, Oncology and Immunology, Moscow, Russian Federation

*Allergy, Asthma & Clinical Immunology* 2023, **19**(Suppl 1):P-41

**Background:** Children with Hereditary Angioedema due to C1-esterase inhibitor deficiency are a diverse cohort of patients, with special physiological and psychological characteristics. Recurrent episodes of edema often represent life-threatening states. Currently, a number of drugs to combat attacks are widely available. The most urgent question for a treating physician is a choice of a drug from the available range, taking into account mechanism of action, route of administration and other parameters.

**Method:** The objective of this retrospective study was to assess the equivalence and safety of Icatibant and C1-INH concentrate in treatment of HAE attacks. The inclusion criteria were the age of patients 0–18 years, the confirmed HAE diagnosis, at least one attack during the study period, administration a study drug within 24 h of the onset of the first symptoms of edema. The study includes 34 patients (33 with C1-INH deficiency and one patient with its functional defect). The age at inclusion in the study varied from 3 to 17 years (Me—12 years). The observation time was 1 to 5 years (Me 3.5 years). Patients were given Icatibant subcutaneously or C1-INH concentrate intravenously.

**Results:** All 34 patients (16 children and 18 juveniles) received Icatibant treatment, 27 patients (14 children and 13 juveniles) received C1-INH concentrate. A total of 302 episodes of attacks were analyzed, of which 225 (74.5%) were treated with Icatibant, 77 (25.5%)—with C1-INH concentrate. The edema reduction after 30 min of a drug administration was equivalent for both drugs. Time to edema abatement was shorter for C1-INH concentrate than Icatibant drug.

**Conclusion:** Based on research and considering the availability of drugs in all age groups as well as many similar efficiency and safety results, when choosing drugs for treatment of edema, it is necessary to take into account the satisfactory venous access, especially considering the age of patients, ensuring the most rapid introduction of drugs, from the onset of edema, taking into account the distance of patients from medical organizations as well as the preferences of patients and/or their parents.

## P-42 Prevailing swelling of the abdominal cavity, as an atypical course of HAE

### Liudmyla Zabrodska

#### SI Institute of Otolaryngology n.a. prof. O.S. Kolomiychenko of NAMS of Ukraine, Kyiv, The Ukraine

*Allergy, Asthma & Clinical Immunology* 2023, **19(Suppl 1): **P-42

**Background:** HAE can appear in childhood, or, more often, in adolescence, or in an already adult patient. Either case, HAE will accompany people to end of their lives. The disease may become more intense or appear less often. As one of the symptoms of HAE, children have pain only in the abdominal cavity, and it is a frequent phenomenon for children but not for adults.

**Results:** 27 people were surveyed and according to that survey, edema of the abdominal cavity is about 45% of all swellings. 16 out of 27 patients experienced swelling of the abdominal cavity during a 3 month period (Fig. 1).

**Figure 1 (abstract P-42):**
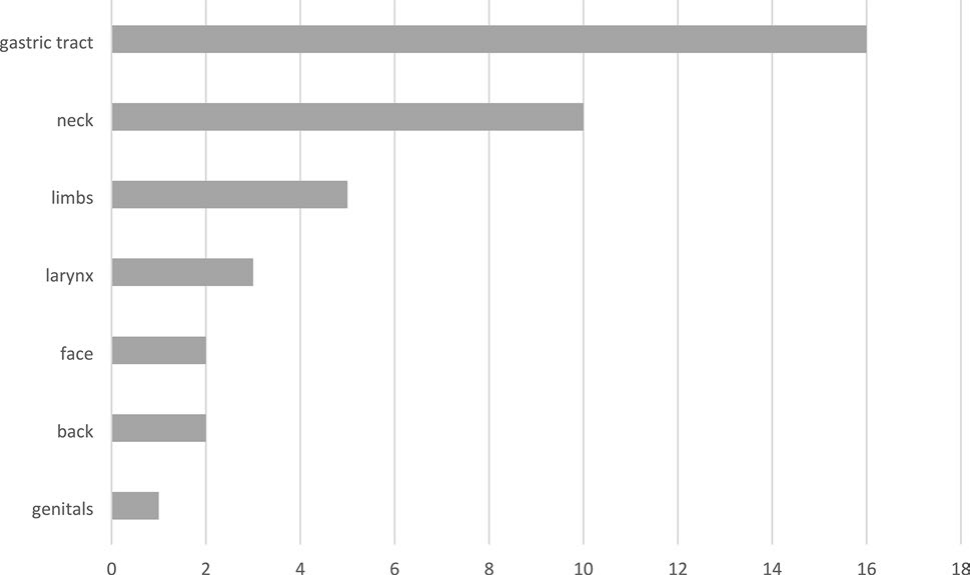
Edema of the abdominal cavity, according to a survey of 27 patients with HAE

The presented clinical case is distinguished by atypical HAE symptoms A patient did not seek help because of the swelling of the limbs, face, and even larynx. The "visible" swelling of the skin in the patient, a 40-year-old man, was extreme occasionally, 1–2 swellings per year, after physical work, overfatigue, in the background SARS or acute tonsillitis. Yet they did seek the medical help because of periodic pains gastrointestinal tract, cutting, intense, often were accompanied by vomiting and nausea, flatulence, deforming wall of the abdominal cavity and bothered him since puberty (13 years). Such pains bothered the patient 2–4 times a month, so the patient had to seek help from a gastroenterologist. Taking painkillers, antihistamines, antispasmodics was not effective. It was discovered and treated during the follow-up examination.

Helicobacter pylori, but it almost did not affect the course and intensity of pain syndrome. During the event FGDS was detected during the "pain syndrome" in the stomach edema of the mucous membrane, decreased peristalsis and a significant amount liquid in the stomach—500 ml, with normal fasting—50–100 ml. The obtained results confirmed type I. Comparing the localization and frequency of edema throughout the year, we see a significant difference. The abdominal cavity prevails, 85% in this presenteed case, which accompany a severe course of HAE. Accordingly, swelling limbs—hands 5%, legs—5%, face—5%, larynx—5%. Therefore, establishing a diagnosis was challenging, yet extremely important.

**Conclusion:** Clinicians need to be included in the differential diagnosis of recurrent abdominal pain syndrome HAE cavity.

All patients gave explicit permission for their information to be published.

## P-43 A patient who was diagnosed with Hereditary Angioedema at two different times

### Beáta Visy^1,2^, Hanga Réka Horváth^2^, Dóra Zsófia Lukács^2^, Lilian Varga^2^, Henriette Farkas^2^

#### ^1^Heim Pál National Institute of Pediatrics, Budapest, Hungary; ^2^Hungarian Angioedema Center of Reference and Excellence, Semmelweis University, Budapest, Hungary

*Allergy, Asthma & Clinical Immunology* 2023, **19**(Suppl 1):P-43

A 58-year-old female patient was diagnosed with type 1 Hereditary Angioedema due to C1-inhibitor deficiency (C1-INH-HAE) during family screening.

Her clinical symptoms began at the age of 25 with facial and laryngeal oedema following tooth extraction. At that time, the symptoms were considered as an allergic reaction to the local anaesthetic drug used during the procedure. Subsequently, she experienced facial and laryngeal oedema after each tooth extraction. She reported limb oedema, crampy abdominal pain, vomiting and nausea 1–2 times a year.

At the age of seventeen, she was diagnosed and treated with discoid lupus erythematosus (DLE). During regular follow-up examinations, several complement tests were performed to follow up with the activity of DLE. Reduced C4 level was measured. The laboratory expert completed the complement study with the measurement of C1-INH antigenic level and functional activity. Both values were decreased, and the laboratory diagnosis was C1-INH HAE type 1. The dermatologist focused on the C4-level and neglected the C1-INH level.

When the patient was 56 years old, her 14-year-old grandson experienced limb oedema on three occasions. On one occasion, the hand oedema was preceded by trauma and surgical exploration was performed. At the age of fifteen, abdominal ultrasound scan was performed and showed free abdominal fluid during crampy abdominal pain episode. After three episodes of limb oedema and two episodes of crampy abdominal pain, the child was referred to our centre by a dermatologist. At our centre, C1-INH HAE was diagnosed by complement testing.

We confirmed HAE in the boy's mother, his grandmother with DLE and his sister by family screening. All of them experienced angioedema symptoms for several years, which were misdiagnosed as an allergic disease.

In case of the grandmother, C1-INH-HAE was diagnosed for the second time.

If the dermatologist had taken the initial laboratory diagnosis of the lupus patient into account, invasive surgery could have been avoided. In this case study, we would like to highlight the appropriate dialogue between laboratory experts and clinicians.

All patients (or their guardians) gave explicit permission for their information to be published.

## P-44 The patient journey: from mesenteric panniculitis and angioedema to Acquired Angioedema

### Ricardo Zwiener^1,^*, Juan Gómez^1^, José Trucco^2^

#### ^1^Allergy and Immunology Department, Hospital Universitario Austral, Pilar, Argenina; ^2^Hematology department, Hospital Universitario Austral, Pilar, Argenina

##### *Correspondence: ricardozwiener@hotmail.com

*Allergy, Asthma & Clinical Immunology* 2023, **19**(Suppl 1):P-44

Acquired Angioedema due to C1-inhibitor deficiency (C1-INH-AAE) is a rare disease compared to Hereditary Angioedema due to C1-inhibitor deficiency (C1-INH-HAE). Both conditions have similar clinical manifestations, but onset of symptoms is later in C1-INH-AAE, and there is no family history of angioedema and facial edema is more frequent in C1-INH-AAE [1].

We present the case of a 72-year-old male patient who complained of recurrent acute abdominal pain associated with vomiting. A CT scan revealed mesenteric panniculitis, which was treated with multiple antibiotics. The patient also experienced an episode of tongue edema without wheals, refractory to treatment with corticoids and antihistamines. He had no relevant medical history or regular medication, and no family history of angioedema. Bradykinin-mediated angioedema was suspected and lab test was performed.

**Materials and methods:** Serum, citrated plasma, and EDTA-blood were collected from the patient, and lab tests confirmed Acquired Angioedema (C1-INH 8,88 mg/dl, Functional C1-INH 40% (154%). C1q 24 mg/dl).

Hematology evaluation revealed atypical lymphocytes and a clonal B lymphoid population (CD 45 +  + , HLA +  + , CD19 +  + , CD20 + +) in flow cytometry. Neck, chest, and abdomen CT scans showed no enlarged lymph nodes, no bone lesions, and a normal spleen. Bone marrow examination revealed a B lymphoid clonal population with the same immunophenotypic pattern, consistent with a marginal zone lymphoma.

**Results:** The patient had recurrent episodes of peripheral angioedema (facial, hands, and genital) with fast response to Icatibant on-demand treatment.

After the diagnosis of Acquired Angioedema secondary to lymphoma, we treated the underlying condition with Rituximab, despite the patient not meeting the classic criteria for treating his lymphoma. Treating the underlying lymphoma would improve and reduce the frequency and severity of angioedema attacks. Despite persistent low levels of Antigenic and C1q after one year of treatment, there were no more episodes of edema.

**Conclusions:** Acquired Angioedema is a rare condition that is classified into two subtypes: Type I, which is associated with lymphoproliferative disorders, and Type II, which is linked with autoantibodies against C1-esterase inhibitor (C1-INH) [2]. Treatment focuses on symptom control with therapies that regulate bradykinin activity and treatment of any underlying conditions. Rituximab has been used successfully to treat C1-INH-AAE [3]. This case highlights the importance of addressing the underlying cause of Acquired Angioedema, as it can significantly improve the patient's quality of life and prevent future attacks. To our knowledge, this is the first report in the literature of an AEA presenting as recurrent episodes of mesenteric panniculitis.

All patients gave explicit permission for their information to be published.

We appreciate Lionel Alfie support.

**References**

1. Andrea Zanichelli et al. Diagnosis, Course, and Management of Angioedema in Patients With Acquired C1-inhibitor Deficiency. JACI PRACT 2017. Vol 5. N5 1307-1313.
2. Seth Ilias Breitbart 1, Leonard Bielory. Acquired Angioedema: Autoantibody associations and C1q utility as a diagnostic tool. 2010 Sep-Oct;31(5):428-34. http://dx.doi.org/10.2500/aap.2010.31.3361I
3. Iris M. Otani, MDa, Aleena Banerji, MD. Acquired C1-inhibitor Deficiency. Immunol Allergy Clin N Am - (2017) http://dx.doi.org/10.1016/j.iac.2017.03.002

## P-45 An uncommon case of postpartum venous thrombosis in a patient with C1-INH-HAE

### Francesco Giardino^1,^*, Riccardo Senter^2^, Sergio Neri^1^, Agostino Rizzotto^1^, Pietro Castellino^1^, Paola Triggianese^3^, Andrea Zanichelli^4^, Mauro Cancian^2^

#### ^1^Azienda Ospedaliero-Universitaria Policlinico “G. Rodolico-San Marco”, Catania, Italy; ^2^Azienda Ospedale-Università di Padova, Padova, Italy; ^3^Policlinico Tor Vergata, Roma, Italy; ^4^IRCCS Policlinico San Donato, San Donato Milanese, Italy

##### *Correspondence: f.giardino@policlinico.unict.it

*Allergy, Asthma & Clinical Immunology* 2023, **19**(Suppl 1):P-45

**Background:** Pregnancy in women affected by Hereditary Angioedema (HAE) can modify the course of the disease in an unpredictable manner [1, 2]. C1-inhibitor concentrate is considered the preferred drug for precautionary reasons and it is used both on-demand and, if appropriate, in a prophylactic regimen [3]. Although few case reports of thrombosis ascribed to C1-inhibitor concentrate were published [4], this medication is not commonly considered a prothrombotic factor in clinical practice.

**Case report:** A 30 years old female with an established diagnosis of type 1 HAE had a dramatic increase of frequency and intensity of attacks after discovering being pregnant.

Prophylaxis with intravenous C1-inhibitor was initiated: 1000 units twice/week were administered for two months, later escalated to three times/week due to recrudescence of attacks.

When pregnancy arrived to full-term the patient underwent a caesarian section, few hours after the last administration of C1-inhibitor. The procedure was uneventful, and the newborn was healthy. However, the day after, a sudden pain at the right flank occurred and abdominal ultrasound showed a floating thrombus in inferior cava vein, confirmed by CT scan. The right ovarian vein was involved.

A hypothetical etiologic role of C1-inhibitor concentrate in the generation of the thrombosis was considered; therefore, prophylaxis with C1-inhibitor concentrate was precautionary suspended and icatibant was prescribed as on-demand strategy for angioedema attacks.

Fondaparinux was initiated, followed by an oral direct anticoagulant. A thrombophilic screening yielded negative results. Breastfeeding was interrupted after patient’s request.

In the following months frequency and intensity of attacks rapidly declined; after re-assessment of the suspect adverse event, C1-inhibitor concentrate was deemed not related to the thrombosis and reintroduced as on-demand treatment without further adverse events.

**Conclusion:** A precautionary approach was followed in this patient: a front of a severe unexpected suspected adverse reaction, C1-inhibitor concentrate was temporarily suspended. However, ovarian vein thrombosis is a possible complication of childbirth, occurring in about 2% of the caesarian sections [5]. The thrombus can extend itself proximally and in 15% of the cases pulmonary embolism occurs, with potential fatal outcome.

Since the characteristic of the thrombosis pointed to a parturition-related complication and most of thrombosis observed with C1-inhibitor administration seems strictly linked to the presence of a central vein access, C1-inhibitor concentrate was deemed not related to the occurrence of thrombosis.

All patients gave explicit permission for their information to be published.

**References**

1. Triggianese P, Senter R, Petraroli A, et al. Pregnancy in women with Hereditary Angioedema due to C1-inhibitor deficiency: Results from the ITACA cohort study on outcome of mothers and children with in utero exposure to plasma-derived C1-inhibitor. Front Med (Lausanne). 2022 Sep 14;9:930403.
2. Czaller I, Visy B, Csuka D, Fust G, Toth F, Farkas H. The natural history of hereditary an gioedema and the impact of treatment with human C1-inhibitor concentrate during pregnancy: a long-term survey. Eur J Obstet Gynecol Reprod Biol. 2010;152(1):44-49.
3. Caballero T., H. Farkas, L. Bouillet, et al. International consensus and practical guidelines on the gynecologic and obstetric management of female patients with Hereditary Angioedema caused by C1-inhibitor deficiency. J.Allergy clin Immunol. 2012 Feb; 129(2):308-20.
4. Seungjong M Yoo, David A Khan. Implantable venous access device associated complications in patients with Hereditary Angioedema. J Allergy Clin Immunol Pract. 2013 Sep-Oct; 1(5):524-5.
5. N.Riva, J. Calleja-Agius Ovarian Vein Thrombosis: A Narrative Review. Hamostaseologie. 2021 Aug; 41 (4):257-266.

## P-46 A young woman with C1-INH deficiency refractory to various treatments: the relevance of having all therapeutic strategies available

### Beatrice Piazza^1^, Riccardo Senter^2^, Cinetto Francesco^1^, Marcello Rattazzi^1^, Mauro Cancian^2^

#### ^1^Department of Medicine, University of Padua, Treviso, Italy; ^2^Azienda Ospedale-Università di Padova, Padova, Italy

*Allergy, Asthma & Clinical Immunology* 2023, **19**(Suppl 1):P-46

**Background:** It is well known that abdominal symptoms of Hereditary Angioedema (HAE) can mimic acute abdomen, sometimes leading to urgent laparotomy [1]. On the other side, it is uncommon that HAE presents itself with chronic abdominal disorders in clinical practice, even if sub-continuous abdominal attacks could theoretically result in this clinical scenario. New effective treatments have been approved in the last years and have made possible to manage very complicated cases, also when apparently refractory.

**Case report:** A 14-years old female patient, with no family history of angioedema, presented with acute abdomen to the emergency room in November 2018. The clinical history was also significant for intermittent abdominal pain and sporadic cutaneous and/or sub-cutaneous angioedema without urticaria. A CT scan revealed extensive peritoneal fluid collection and the patient underwent explorative laparoscopy, where a tubaric cyst was removed. After gynecologic consultation she was discharged with estro-progestinic medications. Her following years were characterized by sub-continuous abdominal pain, severe stypsis with laxative-dependence and nervous anorexia, which was treated for several months in a specialized structure as an inpatient. At the age of 17 an episode of subcutaneous angioedema, involving hands, finally prompted to the assessment of C1-inhibitor and C4, and a diagnosis of type 1 HAE was made. After the withhold of estrogens, stypsis and chronic abdominal pain receded, but abdominal attacks were still frequent and poorly controlled by on-demand treatment with both Icatibant and/or intravenous C1-inhibitor concentrate (at dose of 20 units/kg). Therefore, subcutaneous treatment with Lanadelumab 300 mg every 14 days was initiated, but not effective. The prophylactic therapy was changed to intravenous C1-inhibitor concentrate twice a week, without achieving an adequate control of attacks. Moreover, the patient started to have very poor venous accesses. The correctness of the diagnosis was questioned; however, an abdominal ultrasound, performed during acute attack, supported the diagnosis showing free peritoneal fluid, that disappeared after symptoms improvement. Subcutaneous C1-inhibitor was then implemented, with excellent results.

**Conclusion:** The abdominal involvement in HAE is often recognized by the response to on-demand therapy. When abdominal attacks show a sub-continuous course and appear refractory to the treatment, the diagnosis of HAE could be questioned; in this setting abdominal ultrasound can help. All available treatments must be considered when patients show a refractory or atypical presentation.

All patients (or their guardians) gave explicit permission for their information to be published.

**References**

1. Maurer M, Magerl M, Betschel S, et al. The international WAO/EAACI guideline for the management of Hereditary Angioedema—The 2021 revision and update. World Allergy Organ J. 2022;15(3):100627.

## P-47 Safety of COVID-19 vaccines in patients with angioedema with C1-inhibitor deficiency: data from Italian Network for Hereditary and Acquired Angioedema (ITACA)

### Roberta Parente^1^, Silvio Sartorio^2^, Luisa Brussino^3^, Stefano Pucci^4^, Oliviero Rossi^5^, Giuseppe Spadaro^6^, Davide Firinu^7^, Riccardo Senter^8^, Massimo Triggiani^1^, Mauro Cancian^8^, Andrea Zanichelli^9^

#### ^1^University of Salerno, Salerno, Italy; ^2^University of Milan, Milan, Italy; ^3^University of Turin, Turin, Italy; ^4^Civitanova Marche Hospital, Italy; ^5^University of Florence, Florence, Italy; ^6^University of Naples, Naples, Italy; ^7^University of Cagliari, Cagliari, Italy; ^8^University of Padua, Padua, Italy; ^9^IRCSS San Donato Polyclinic, Italy

*Allergy, Asthma & Clinical Immunology* 2023, **19**(Suppl 1):P-47

**Background:** Angioedema with C1-inhibitor deficiency (C1-INH-AE) is a rare disease characterized by recurrent and unpredictable attacks of angioedema without hives, with a heterogeneous phenotypes in terms of severity and site of attacks. The genetic form is a rare autosomal dominant disorder due to C1-inhibitor deficiency (type I) or dysfunction (type II).

In patients with C1-INH-AE, the inadequate control of the contact system causes excessive bradykinin formation with localized and transient increase in vascular permeability, resulting in angioedema attacks. Eliciting factors of the attacks include trauma, emotional factors, medical procedures and infections. Certain studies suggest that mRNA vaccines potentially represent an eliciting factors of angioedema attacks. However, only few data were reported about the safety of COVID-19 vaccines in patients with C1-INH-AE.

**Method:** In this study we collected the data about a population of 208 adult patients with C1-INH-AE (107 females) followed by 11 reference centers in Italy (Milan, Florence, Padua, Turin, Civitanova Marche, Salerno, Naples, Aosta, Ancona, Genoa, Messina). Of those, the majority of patients (89%) had a diagnosis of hereditary C1-INH-AE and 23 patients were diagnosed as acquired C1-INH-AE. In this cohort the mean attack rate was 0.89/months. Long term prophylaxis (LTP) was prescribed in 80 patients with hereditary C1-INH-AE and in 9 patients with acquired C1-INH-AE. The primary aim of this observational study was to collect data on the onset of acute attacks in the 72 h following the COVID-19 vaccination.

**Results:** Between December 2021 and June 2022, 203 patients with C1-INH-AE received Covid 19 vaccines in a controlled medical setting in reference centers. Four hundred and five doses were administered as a part of primary vaccination cycle and 124 doses were given as booster doses. The majority of patients received mRNA vaccines. About 5% of patients received vaccines made using adenovirus vector; in particular ChAdOx1 nCoV-19 vaccines (Astra-Zeneca) and Ad26.COV.2.S (Jassen/Johnson & Johnson). A total of 48 attacks of angioedema occurred within 72 h after the vaccine administration were registered. The majority of them (50%) were abdominal attacks; extremities were involved in 20% and 39% of cases respectively after primary vaccination cycle and booster doses; combined (cutaneous and abdominal) attacks were occurred respectively in 20% and 11% cases. Three patient reported a laringeal attacks after the administration of first dose of Pfizer (in two cases) and of second dose (in one case). However, no hospitalization was required. In all cases, the attacks were successfully treated with icatibant or plasma derived C1-inhibitor. Interestingly, there is no difference in the rate of attacks occurred after vaccines between patients on LTP regimen and those using on demand treatment.

**Conclusion:** Our data suggest that Covid 19 vaccines are safe and tolerable in patients with C1-INH-AE. These patients could be vaccinated with these novel vaccines in a controlled medical setting.

## P-48 Garadacimab for Hereditary Angioedema prophylaxis in adolescents: efficacy and safety from the VANGUARD Phase 3 and 3b open-label extension trial (first interim analysis)

### Markus Magerl^1,^*, Inmaculada Martinez-Saguer^2^, Joshua S. Jacobs^3^, H. Henry Li^4^, Jonathan A. Bernstein^5^, Connie Hsu^6^, Karl V. Sitz^7^, Ingo Pragst^8^, Henrike Feuersenger^8^, Lolis Wieman^9^, Maressa Pollen^9^, Avner Reshef^10^

#### ^1^Institute of Allergology, Charité–Universitätsmedizin Berlin, Corporate Member of Freie Universität Berlin and Humboldt-Universität zu Berlin, Berlin, and Frauhofer Institute for Translational Medicine and Pharmacology ITMP, Immunology and Allergology, Berlin, Germany; ^2^HZRM Haemophilia Center Rhein Main, Mörfelden-Walldorf, Germany; ^3^Allergy & Asthma Clinical Research, Walnut Creek, CA, USA; ^4^Institute for Asthma and Allergy, Chevy Chase, MD, USA; ^5^University of Cincinnati, Department of Internal Medicine Division of Rheumatology, Allergy and Immunology and the Bernstein Clinical Research Center Cincinnati, Cincinnati, OH, USA; ^6^Research Solutions of Arizona, PC, Litchfield Park, AZ, USA; ^7^Little Rock Allergy and Asthma Clinic, Little Rock, AR, USA; ^8^CSL Behring Innovation GmbH, Marburg, Germany; ^9^CSL Behring, King of Prussia, PA, USA; ^10^Allergy, Immunology & Angioedema Center, Barzilai University Hospital, Ashkelon, Israel

##### *Correspondence: markus.magerl@charite.de

*Allergy, Asthma & Clinical Immunology* 2023, **19**(Suppl 1):P-48

**Background:** Efficacy and safety of garadacimab (fully human, monoclonal antibody targeting activated factor XII) for Hereditary Angioedema (HAE) attack prophylaxis were demonstrated in Phase 2 (adults only) [1] and pivotal Phase 3 (VANGUARD) studies [2]. Here, adolescent safety and efficacy data from the pivotal Phase 3 study and open-label extension (OLE, first planned analysis) (NCT04739059) are reported for the first time.

**Methods:** In the pivotal Phase 3 study, after ≥ 1-month run-in, six adolescents (aged 12– ≤ 17 years, HAE type I/II) were randomised (3:2) to receive once-monthly subcutaneous garadacimab 200 mg (n = 4) or placebo (n = 2), respectively, for 6 months after initial garadacimab 400 mg loading dose or volume-matched placebo. All six adolescents from the pivotal Phase 3 study and five additional garadacimab-naïve adolescents entered OLE at same dose regimen (n = 10; one aged 18 years in OLE was analysed thereafter as an adult). Time-normalised monthly attack rate during treatment period was compared against run-in.

**Results:** At OLE first planned analysis, garadacimab exposure ranged from 3.3–15.2 months (inclusive of pivotal Phase 3 study). No serious treatment-emergent adverse events (TEAEs), TEAEs leading to discontinuation, TEAEs of special interest (abnormal bleeding events, thromboembolic events, severe hypersensitivity including anaphylaxis), treatment-related TEAEs, or injection-site reactions were reported in either pivotal Phase 3 or OLE studies. In the pivotal Phase 3 study, 4/6 adolescents (66.7%; 2 treated, 2 placebo) experienced ≥ 1 TEAE (11 TEAEs reported; all were mild [10/11, 90.9%] or moderate [1/11, 9.1%]). In OLE, 6/10 adolescents (60.0%) experienced ≥ 1 TEAE (20 TEAEs reported; all were mild [16/20, 80.0%] or moderate [4/20, 20.0%]).

In the pivotal Phase 3 study, 2/4 garadacimab-treated adolescents (50.0%) were attack-free for the entire 6-month treatment period and 1 adolescent (25.0%) achieved 92.3% attack rate reduction vs run-in; subsequently, all three maintained or achieved attack-free status in OLE. One garadacimab-treated adolescent who initially experienced a 5.8% increase in attack rate vs run-in during the pivotal Phase 3 study, achieved a 45.5% reduction vs run-in in OLE and has been attack-free since July 2022. All 5 garadacimab-naïve adolescents in OLE were responders: 3 adolescents were attack-free (range of exposure 3.3 − 12.5 months), one achieved 93.8% reduction over 15.2 months and one achieved 65.7% reduction over 3.3 months vs run-in.

**Conclusions:** Consistent with adult data from the pivotal Phase 3 study and OLE, once-monthly garadacimab demonstrated a favourable safety profile and had sustained efficacy as routine prophylaxis to prevent HAE attacks in adolescents.

**References**

1. Craig T, Magerl M, Levy DS, et al. Prophylactic use of an anti-activated factor XII monoclonal antibody, garadacimab, for patients with C1-esterase inhibitor-deficient Hereditary Angioedema: a randomised, double-blind, placebo-controlled, phase 2 trial. Lancet. 2022; 399:945–955.
2. Craig T, Reshef A, Li H, et al. Efficacy and safety of garadacimab, a factor XIIa inhibitor for Hereditary Angioedema prevention (VANGUARD): a global, multicentre, randomised, double-blind, placebo-controlled, phase 3 trial. Lancet. 2023. Published online February 28, 2023, DOI: https://doi.org/10.1016/S0140-6736(23)00350-1.

## P-49 Transmission patterns in C1-INH deficiency Hereditary Angioedema favours a wild-type male offspring: our experience at Chandigarh, India

### Ankur Kumar Jindal^1,^*, Sanghamitra Machhua^1^, Sanchi Chawla^1^, Anit Kaur^1^, Rahul Tyagi^1^, Ishita Jangra^1^, Supreet Basu^1^, Prabal Barman^1^, Archan Sil^1^, Reva Tyagi^1^, Sendhil M. Kumaran^2^, Sunil Dogra^2^, Manpreet Dhaliwal^1^, Saniya Sharma^1^ Amit Rawat^1^, Surjit Singh^1^

#### ^1^Allergy Immunology Unit, Department of Pediatrics, Advanced Pediatrics Centre, PGIMER, Chandigarh, India; ^2^Department of Dermatology, Venereology and Leprology, PGIMER, Chandigarh, India

##### *Correspondence: ankurjindal11@gmail.com

*Allergy, Asthma & Clinical Immunology* 2023, **19**(Suppl 1):P-49 

**Background:** Deficiency of C1-INH protein (caused by pathogenic variants in the *SERPING1* gene) is the commonest pathophysiological abnormality (in ~ 95% cases) in patients with Hereditary Angioedema (HAE) [1, 2]. C1-INH protein provides negative control over kallikrein–kinin system (KKS). Although the inheritance of the C1-INH-HAE is autosomal dominant, female predominance has often been observed in patients with HAE [3, 4]. However, there is a paucity of literature on transmission discordance between male and female offspring in patients with HAE.

**Methods:** Pedigree charts of 41 families with a confirmed diagnosis of HAE-C1-INH and a pathogenic variant in the *SERPING1* gene were analysed. Patients with HAE who had had at least one child were included for analyses to assess the risk of transmission from the father or mother to their offspring.

Results: Overall, 49.1% (172/350) of all offspring inherited the genetic defect. In the subgroup analyses, 53.9% (82/152) female offspring and 45.4% (90/198) male offspring inherited the genetic defect. This difference was not statistically significant. Fathers with *SERPING1* gene mutation had a statistically significant skewed transmission favouring wild allele to male offspring (41.8%, 41/98; p < 0.02). There was no statistically significant difference when a father transmitted the mutation to a female offspring or a female transmitted the mutation to either male or female offspring (Table 1).

**Table 1 (abstract P-49):** Inheritance pattern of HAE-C1-INH with SERPING1 mutation

Total offspring			Total male offspring			Total female offspring		
Mutant	Wild type	P value	mutant	Wild type	P	mutant	Wild type	p
172 (49.1%)	178 (50.8%)	0.6	90 (45.4%)	108 (54.5%)	0.07	82 (53.9%)	70 (46%)	0.1

Paternal Inheritance						Maternal Inheritance					
Male offspring			Female offspring			Male offspring			Female offspring		
Mutant	Wild type	p	Mutant	Wild type	p	Mutant	Wild type	p	Mutant	Wild type	p
41 (41.8%)	57 (58.1%)	**0.02**	39 (52.7%)	35 (47.3%)	0.5	49 (49%)	51 (51%)	0.7	43 (55.1%)	35 (44.8%)	0.2

**Conclusion:** Results of the study suggest that the transmission pattern of *SERPING1* gene mutation favours the transmission of wild-type alleles in males, especially when the father is the carrier. This could be because of a selection of wild-type male sperms during spermatogenesis. KLK system has been reported to play a crucial role in the regulation of spermatogenesis. Further research is needed to explain the discordance in the inheritance pattern of HAE.

**References**

1. Jindal AK, Rawat A, Kaur A, Sharma D, Suri D, Gupta A, Garg R, Dogra S, Saikia B, Minz RW, Singh S. Novel SERPING1 gene mutations and clinical experience of type 1 Hereditary Angioedema from North India. Pediatr Allergy Immunol. 2021 Apr;32(3):599-611.
2. Förster TM, Magerl M, Maurer M, Zülbahar S, Zielke S, Inhaber N, et al. HAE patient self-sampling for biomarker establishment. Orphanet J Rare Dis. 2021;16(1):1–9.
3. Agostoni A, Cicardi M. Hereditary and acquired C1-inhibitor deficiency: biological and clinical characteristics in 235 patients. Medicine. 1992;71:206–215
4. Bork K, Meng G, Staubach P, Hardt J. Hereditary Angioedema: new findings concerning symptoms, affected organs, and course. Am J Med. 2006 Mar;119(3):267-74.

## P-50 Profile of Hereditary Angioedema from Nepal—A speck of imprint on Everest

### Dharmagat Bhattarai^1^, Aaqib Zaffar Banday^2^, Apar Pokharel^3^

#### ^1^Advanced Centre for Immunology & Rheumatology, Kathmandu, Nepal; ^2^Government Medical College, Srinagar, Kashmir, India; ^3^College of Medical Sciences, Chitwan, Nepal

*Allergy, Asthma & Clinical Immunology* 2023, **19**(Suppl 1):P-50

**Rationale:** Hereditary Angioedema (HAE) is an autosomal dominant immunodeficiency disorder with recurrent episodes of non-pruritic oedema of various tissues of body. Many cases of HAE are missed or mistreated in resource-limited settings. With availability of single immunologist, there is paradigm shift in diagnosis and management of recurrent angioedema in Nepal. We describe the profile of patients with HAE at a tertiary care centre from Nepal.

**Methods:** Case records of patients diagnosed as HAE at the tertiary private care centre in Kathmandu during Aug 2021-January 2023 were analysed. The lead author (DB) collated data from all patients. Diagnosis and treatments were based on internationally acclaimed guidelines. 

**Results:** Our cohort of 16 patients with recurrent angioedema consists of 6 patients with HAE. Five are type 1 HAE with low C4 and C1-esterase inhibitor (C1-INH). One patient had normal C1-INH. Median age of onset of symptoms and diagnosis were 7.5 and 17 years, respectively. Two patients had pathogenic *SERPING1* gene mutation. One patient with normal C1-INH was found to have kininogen (KNG1) mutation. Report of genetic analysis of two patients are pending. Remaining could not perform tests due to financial constraints and unavailability of genetic tests in country. All patients were kept on long term prophylaxis with tranexamic acid or attenuated androgens. Patients in intensive care were also treated with fresh-frozen plasma infusions. One patient is recently listed for C1-INH therapy.

**Conclusion:** We present first Nepalese cohort with proven cases of HAE. Lack of awareness and diagnostic facilities coupled with socioeconomic limitations have resulted in misdiagnosis, inappropriate treatment, and poor outcome in HAE in resource-limited settings.

